# An Antimicrobial Peptide Induces *FIG1*-Dependent Cell Death During Cell Cycle Arrest in Yeast

**DOI:** 10.3389/fmicb.2018.01240

**Published:** 2018-06-14

**Authors:** Vladimir J. Arellano, Paula Martinell García, Jonathan G. Rodríguez Plaza, Maria T. Lara Ortiz, Gabriele Schreiber, Rudolf Volkmer, Edda Klipp, Gabriel Del Rio

**Affiliations:** ^1^Departamento de Bioquímica y Biología Estructural, Instituto de Fisiología Celular, Universidad Nacional Autónoma de México, Mexico City, Mexico; ^2^Theoretische Biophysik, Humboldt-Universität zu Berlin, Berlin, Germany; ^3^Institut für Medizinische Immunologie, Charité – Universitätsmedizin Berlin, Berlin, Germany; ^4^Leibniz-Institut für Molekulare Pharmakologie, Berlin, Germany

**Keywords:** antimicrobial peptide, cell cycle arrest, cell death, *Saccharomyces cerevisiae*, cytometry, microscopy, gene deletions, pheromone pathway

## Abstract

Although most antibiotics act on cells that are actively dividing and non-dividing cells such as in microbe sporulation or cancer stem cells represent a new paradigm for the control of disease. In addition to their relevance to health, such antibiotics may promote our understanding of the relationship between the cell cycle and cell death. No antibiotic specifically acting on microbial cells arrested in their cell cycle has been identified until the present time. In this study we used an antimicrobial peptide derived from α-pheromone, IP-1, targeted against MATa *Saccharomyces cerevisiae* cells in order to assess its dependence on cell cycle arrest to kill cells. Analysis by flow cytometry and fluorescence microscopy of various null mutations of genes involved in biological processes activated by the pheromone pathway (the mitogen-activated protein kinase pathway, cell cycle arrest, cell proliferation, autophagy, calcium influx) showed that IP-1 requires arrest in G_0_/G_1_ in order to kill yeast cells. Isolating cells in different cell cycle phases by elutriation provided further evidence that entry into cell cycle arrest, and not into G_1_ phase, is necessary if our peptide is to kill yeast cells. We also describe a variant of IP-1 that does not activate the pheromone pathway and consequently does not kill yeast cells that express the pheromone’s receptor; the use of this variant peptide in combination with different cell cycle inhibitors that induce cell cycle arrest independently of the pheromone pathway confirmed that it is cell cycle arrest that is required for the cell death induced by this peptide in yeast. We show that the cell death induced by IP-1 differs from that induced by α-pheromone and depends on *FIG1* in a way independent of the cell cycle arrest induced by the pheromone. Thus, IP-1 is the first molecule described that specifically kills microbial cells during cell cycle arrest, a subject of interest beyond the process of mating in yeast cells. The experimental system described in this study should be useful in the study of the mechanisms at play in the communication between cell cycle arrest and cell death on other organisms, hence promoting the development of new antibiotics.

## Introduction

Programmed cell death (PCD) occurs in both multicellular and unicellular organisms and constitutes a homeostatic mechanism in cell populations (Andersen and Kornbluth, 2013). There is significant evidence supporting the existence in *Saccharomyces cerevisiae* of genetic programs for induction of cell death (Munoz et al., 2012). In recent decades, it was shown that *S. cerevisiae* features PCD during sexual mating (Severin and Hyman, 2002). In such mating processes, haploid MATα (mating type α) cells produce α-pheromone as a signal to induce the mating response in MATa (mating type a) cells and vice versa; this response involves multiple intracellular signaling events that start with the activation of the α-pheromone receptor (Ste2p), which upregulates the mitogen-activated protein kinase (MAPK) pathway, which in turn leads to cell cycle arrest (G_0_/G_1_) and morphological changes collectively described as the shmoo phenotype (Dohlman and Thorner, 2001). After this primary signal, the MATa cells make a decision: to mate with a MATα cell, to recover from the arrest, or to activate a cell death program. Even under normal mating conditions, 6% of cells will fail to find a mating partner and die through an apoptosis-like mechanism; alternatively, when the pheromone concentration is above physiological concentrations, up to 25% of cells die as a consequence of three independent waves of non-apoptotic cell death (Zhang et al., 2006). Thus, cell death can take place during cell cycle arrest in *S. cerevisiae*. Whether or not this cell death depends on cell cycle arrest is still unknown, but there is evidence for a relationship between these two molecular processes. For instance, the induction of random death by α-pheromone as a consequence of transcriptional squelching has been excluded in consideration of the uniformity of cell cycle arrest induced by α-pheromone (Dolan, 1996); chitin synthesis is required for cell cycle progression and viability, and its production is induced by α-pheromone (Shaw et al., 1991); slow and fast cell death induced by α-pheromone were inhibited by null mutants of genes that regulate cell cycle arrest (*FUS3* and *FAR1*) (Zhang et al., 2006). Thus, mating involves cell cycle arrest and PCD, and while both processes may be induced by the pheromone, it is not known whether or not the PCD induced by the pheromone takes place during the cell cycle arrest. In that sense, *S. cerevisiae* and its mating process constitute a convenient experimental system to study the antibiotic action of a molecule during cell cycle arrest. Furthermore, the identification of antibiotic molecules that induce PCD in cells arrested in their cell cycle will be relevant for the development of new classes of antibiotics; such antibiotics have not been described in the literature, only cell cycle disruptors (Shapiro and Harper, 1999; Errington, 2010; Sass and Brötz-Oesterhelt, 2013; Senese et al., 2014).

**FIGURE 1:**
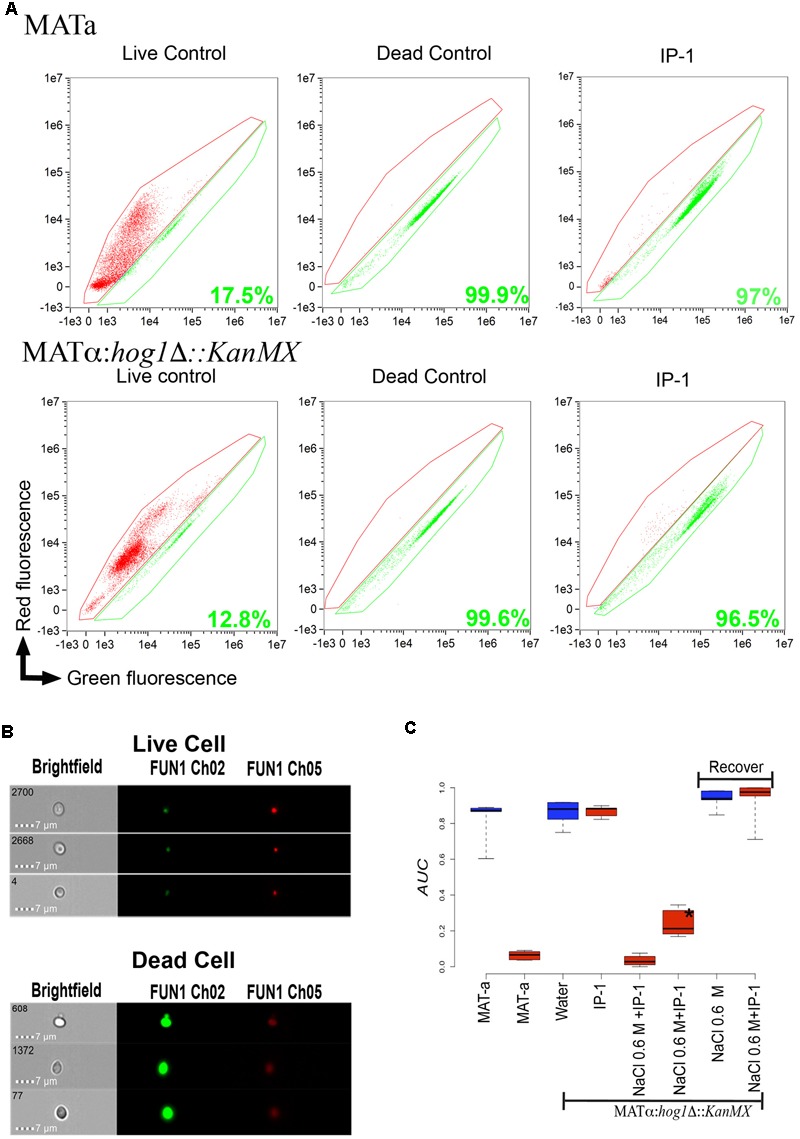
Quantification of cell death induced by IP-1. **(A)** Dot plot, fluorescence intensity of dying cells (red fluorescence) vs. fluorescence intensity of living cells (green fluorescence); the areas marked in red and green identify the dead and live cells, respectively; the green number in each plot corresponds to the percentage of dead cells found in each treatment for MATa and MATα:*Δhog1* cells. **(B)** The panels show the images of cells recorded by the Amnis instrument (see section “Materials and Methods”) at a magnification of 64× in bright field and red or green fluorescence; in the top panel an example of live cells (cells not exposed to IP-1) is presented and the lower panel shows an example of dead cells (cells heated at 90°C for 1 h). **(C)** Box plot of the relative area under growth curves (AUCs) of cells (MATa, MATα:*hog1Δ::kanMX*) exposed to IP-1 (10 μM; red boxes) or water (blue boxes) as control; MATα:*hog1Δ::kanMX* cells are represented as *Δhog1* for abbreviation. The relative AUCs of MATα:*hog1Δ::kanMX* cells that were arrested by exposure to NaCl and then allowed to recover from this arrest are indicated by the “Recover” label. The figure summarizes the results obtained from three independent experiments.

Previously, we described a family of antimicrobial peptides derived from α-pheromone (Rodriguez Plaza et al., 2012), referred to as Iztli peptides (IPs). These peptides include the 13 amino acid residues of the α-pheromone sequence, together with a six amino acid residue addition at the N-terminus of this sequence; the addition of these six residues gives the peptide sequences the same physicochemical properties of known antimicrobial peptides and consequently these peptides were expected to show antimicrobial activity. α-pheromone was included in these peptides in order to target the peptides’ antimicrobial action against *S. cerevisiae* cells that express the α-pheromone receptor (e.g., MATa cells). Our initial characterization of one of these peptides, IP-1, showed that IP-1 maintained pheromone-like activity (e.g., MATa cells exposed to IP-1 exhibited the shmoo phenotype) and inhibited the growth of *S. cerevisiae* cells only if the latter expressed the α-pheromone’s receptor; such inhibition of growth was more effective than that achieved by α-pheromone. In the present work, we show that IP-1 induces cell death in MATa or MATα cells upon the induction of cell cycle arrest in G_0_/G_1_ whether through the α-pheromone receptor or otherwise. Our results show that entry into cell cycle arrest, but not entry into G_1_ phase or maintenance of cell cycle arrest, is required for IP-1-induced cell death in MATa cells. This cell death depends not only on cell cycle arrest, but also on *FIG1*, a gene that codes for an integral membrane protein required for cell death induced by the pheromone at non-physiological concentrations. The potential uses of IP-1 in basic and applied research are analyzed in light of these results.

## Materials and Methods

All biological and chemical materials were handled in accordance with Official Mexican Standards (NOM-087- SEMARNAT-SSA12002).

### Strains

The *S. cerevisiae* strains used in this work are listed in **Table 1**. The null mutant strains were acquired from Open Biosystems and the strains carrying the CDC28-as1 mutant were kindly provided by Prof. Alejandro Colman Lerner.

**Table 1:** Yeast strains used in this study.

Strain	Genotype	Source
BY4741	*HIS3Δ1 LEU2Δ0 MET15Δ0 URA3Δ0*	Open Biosystems, yeast parental strain collection, glycerol stocks
BY4742	*HIS3Δ1 LEU2Δ0 LYS2Δ0 URA3Δ0*	Open Biosystems, yeast parental strain collection, glycerol stocks
BY4741 null mutants	The following genes were replaced with the *KanMX4* marker: *ATG11, AYT1, BRE2, CMS1, COX17, CYC3, DEP1, DNM1, DRS2, ELM1, ERV46, FRT2, FUN14, GAT3, ISA2, MHT1, NTG1, NUP60, OYE2, PEP4, POR1, PSR2, SAW1, SEO1, SIR4, SNC1, SNF7, SPO7, SPO75, STE20, STE4, SWC3, SWD1, SYN8, UBR2, UIP3, UTH1, ATG3, ATG19, TOR1, MEH1, SLM4, GTR2, VAC8, NVJ1, PEP4, AUP1, UTH1, GEA1, IRS4, VTC1, VTC2, VTC3, VTC4, VPS51, VPS52, VPS53, VPS54, YPT6, PBS1, PHO81, RIM15, FIG1, CCH1, MID1, LRG1, RVS161, PEA1, PEA2, STE5, STE7, STE11, STE50, FUS3, KSS1, BEM1, BNI1, KAR4, RME1, HAL9, XBP1, HMS1, SKN7, PUT3, MSN4, MGA1, FKH2*	Open Biosystems, YKO MATa strain collection, glycerol stocks
ACLY394	W303 (MATa): *BAR1Δ CDC28-as1 ΔPRM1::PPRM1-YFP::HIS5+*	Shokat et al., 2000; glycerol stock
ACLY387	W303 (MATa): *BAR1Δ ΔPRM1::PPRM1-YFP::HIS5+*	Shokat et al., 2000; glycerol stock
BY4741 ρ0	*HIS3Δ1 LEU2Δ0 MET15Δ0 URA3Δ0*	This study; glycerol stock

### Peptides

α-Pheromone peptide was obtained from Sigma-Aldrich (T6901) at ≥93% purity. IP-1 was synthesized by AnaSpec, Inc. (United States) at >95% purity. Prof. Rudolf Volkmer at the Charité in Berlin synthesized IP-1-CO-NH_2_ and obtained >95% purity. For each peptide, an initial 1 mg/mL solution of peptide in distilled water was prepared and the actual concentration was determined at 280 nm using a NanoDrop device (Thermo Fisher Scientific, United States); this stock solution was kept at -80°C. The sequences and molecular weights of each peptide used in this study are presented in **Table 2**.

**Table 2:** Peptide sequences used in this study.

Name	Sequence	Molecular weight (g/mol)
IP-1	KFLNRFWHWLQLKPGQPMY	2490
PI-1-CO-NH_2_	KFLNRFWHWLQLKPGQPMY-CO-NH_2_	2490
α-Pheromone	WHWLQLKPGQPMY	1684

### Measuring the Inhibition of Growth Induced by Peptides

An early stationary phase inoculum was obtained from a single colony growing overnight at 30°C in YPD medium (yeast extract 1%, peptone 2%, glucose 2%) of all of the strains used in this study. These inocula were diluted to reach an optical density of 0.12 at 600 nm in a total volume of 100 μL YPD medium and treated with the peptides listed in **Table 2** at 10 μM final concentration, except in the case of pheromone and IP-1-CO-NH_2_, where concentrations of 1, 5, 20, and 40 μM were used when specified. These mixtures were grown in sterile 96-well half-area plates (Corning Costar, United States) and incubated in a Synergy MX Microplate Reader (BioTek Instruments, Inc.) for 24 h at 30°C at a constant orbital shaking (300 rpm) and cell density was estimated by recording the absorbance of the culture at 600 nm every hour. The area under this growth curve (AUC) was obtained by adding the optical density measured each hour. Comparison between the AUCs observed for cells with no treatment and the AUCs exposed to different peptides defined the level of growth inhibition exerted by the peptides. Every null mutant associated with the MAPK pathway was validated by PCR; the oligonucleotides used in these experiments are shown in **Table 3**.

**Table 3:** Oligonucleotides used in this study to verify the proper insertion of the *KanMX4* cassette to generate null mutants.

Oligonucleotide	Sequence (5′ → 3′)
KanC Fwd	TGATTTTGATGACGAGCGTAAT
NatC Fwd	TACCAACAAATACAAGCCTACA
Ste11D Rvs	ATCTTACTTGATTTTATTCCAGGGG
Ste50D Rvs	CATTATCCAAACATGAAAATAAGGC
Ste7D Rvs	TGGTTGTGGCATAAAAATAAAGAAT
Ste5D Rvs	GAATGAAAAGCAATATACGCAAGAT
Fus3D Rvs	AATCACTACTTTGGTAGTTTGACGC
Kss1D Rvs	GTGTTGATATCGCCTCTTTGATTAC
Far1D Rvs	TGCTACAACCATGTTGGTATAATTG
Fig1D Rvs	AAATTTCTGGAGCTTTGTTACATTG
Bem1D Rvs	CATGCATTATGATTGAGTGGAAATA
Tec1D Rvs	GATGTGTATTGGCTGGTTTACTTCT
Flo1D Rvs	CCAATACTACCGGTACTTGTTCTTG
Phd1D Rvs	ATGTTTCAAAAAGGCATCATATTGT

*Oligonucleotide sequences used to verify the proper insertion of the corresponding antibiotic resistance cassette in the deletion mutant strains. KanC Fwd oligonucleotide was the forward primer used to verify the presence of the *KanMX4* cassette while NatC Fwd oligonucleotide was used for the NatMX4 cassette. These were used in conjunction with a reverse primer (Rvs) homologous to a region downstream of the deleted gene.*

### FUN1 Viability Assay

Molecular probes for the LIVE/DEAD Yeast Viability Kit (Invitrogen, United States) were used. Cells were grown in 4 mL minimal synthetic media [for every 100 mL of media, we added 0.67 g of yeast nitrogen base without amino acids (Difco), 0.1 g of potassium phosphate monobasic (J.T. Baker), 2.0 g of glucose (Sigma), and 0.079 g of mixture of amino acids (Formedium)] for 16 h. The cells were diluted to a final optical density of 0.12 at 600 nm. To determine the number of live/dead cells in any condition, 20 μL of these cultured cells were recovered and centrifuged at 13,000 rpm for 10 s, then washed twice with distilled water and finally diluted in 20 μL glucose-HEPES media [2% D-(+)-glucose and 10 mM Na-HEPES, final pH adjusted at 7.2]. The samples were treated with 5 μM FUN1 for 30 min at 30°C and 250 rpm in constant agitation. Fifteen microliters of the stained cultures were analyzed in an Amnis^®^ imaging flow cytometer (ImageStream X Mark II).

### Cell Cycle Arrest of *MATα:hog1Δ::KanMX* Cells by Hyperosmotic Shock

An early stationary phase inoculum was obtained from a single colony growing overnight at 30°C in YPD medium, cells were diluted to an optical density of 0.12 at 600 nm and culture was incubated in a total volume of 100 μL supplemented with YPD medium and 0.6 M sodium chloride for 36 h. IP-1 was added to this culture at a final concentration of 10 μM. As control, cells were treated with the equivalent volume of distilled water.

### Effect of IP-1 on Cells in Different Cell Cycle Stages

BY4741 and BY4742 strains were grown overnight in YPD; the cell density of these cultures was adjusted to OD_600_ = 0.8–1.3 in a photometer (Kinetik). G1 (petit) cells were separated from the other cell cycle phases (G2, S, M) by elutriation with a Beckman Coulter JE-5.0 elutriation system. Cells were centrifuged and resuspended in PBS to stop cell growth. After elutriation, cells were kept on ice and the cell number was determined (CasyCounter). Next, the YPD was inoculated with 2 × 10^6^ cells/mL and these cells were exposed to α-pheromone, IP-1, or water as a control. These cultures were grown for 20 h at 30°C and the optical density was recorded every hour at 600 nm in a FLUOstar OPTIMA plate reader (BMG Labtech).

### Chemically Induced Cell Cycle Arrest

Cells were grown in 4 mL YPD at 30°C at 250 rpm in constant agitation for 16 h; they were then diluted with YPD to an optical density of 0.12 at 600 nm. The culture was grown to an optical density of 0.4 and then treated with 15 μg/mL nocodazole for 2 h. Cells treated with nocodazole were arrested in G_0_/G_1_ phase using 5 μM TBT (tributyltin) for 2 h; it has been reported that under such conditions over 90% of the population was in G_1_/G_0_ phase (Sekito et al., 2014). Samples were taken for analysis of cell cycle and viability as described in Sections “FUN1 Viability Assay” and “Cell Cycle Analysis.”

### Cell Cycle Analysis

Cells with different treatments were diluted to a final optical density of 0.4 at 600 nm, washed and fixed in 70% EtOH/30% distilled water overnight. These fixed cells were then treated with 0.1 mg/mL RNase A (Sigma-Aldrich) at 37°C for 45 min; to stain their DNA, fixed cells were exposed to 5 nM propidium iodide (PI; Molecular Probes, Eugene) and analyzed within the following 6–12 h at 493 nm excitation and 632 nm emission. Samples were analyzed by an ImageStream imaging flow cytometer (Amnis Corporation, Seattle, WA, United States) using INSPIRE acquisition software; 15,000 cells per sample were recorded in bright field and the corresponding fluorescence channel. PI fluorescence and the corresponding bright field images were collected in Channel 4 (595–660 nm) and Channel 1 (420–480 nm), respectively. Images were analyzed using IDEAS v3.0 image analysis software to identify in-focus images (RMS value >50). The measurements generated from the images (aspect ratio vs. area of the bright field channel) were plotted to select single and double cells; single cells had an aspect ratio of 1 and double cells of 0.5. Three regions were selected for further analysis. Cells in G_1_, S, or G_2_/M were visually identified using three morphological criteria: single nuclei and no bud (G_1_), single round nuclei and a visible bud (S-phase), or elongated or divided nuclei and a large bud (G_2_/M).

### Generation of ρ0 Strains of *Saccharomyces cerevisiae*

A colony of MATa *S. cerevisiae* was grown in 2 mL of Complete Supplement Mix (CSM) medium with 5 μL of ethidium bromide (stock solution: 10 mg/mL, sterilized by filtration) at 30°C for 2 days. After this treatment, a sample of 10 μL of this cell solution was added to 2 mL of new CSM medium with 5 μL of ethidium bromide and incubated at 30°C for 2 days. Ten microliters of the cell solution was streaked on a YPD plate and incubated at 30°C for 36 h. A colony was streaked in a new YPD plate and incubated at 30°C for 36 h. To verify the lack of the mitochondrial genome, several colonies from this plate were replicated in YPD and YPEG (3% glycerol, 3% ethanol) plates.

### Effect of IP-1 on Cell Growth in Respiratory and Fermenting Media

From all strains of interest a single colony was grown overnight in YPD medium (yeast extract 1%, peptone 2%, glucose 2%) or YPLac (yeast extract 1%, peptone 2%, lactic acid 2%, ammonium sulfate 0.12%, potassium phosphate monobasic 0.1%) pH 5.5. These cultures were used to inoculate fresh YPD or YPLac media to reach 0.4 optical density (600 nm) and then diluted to 0.03 O.D. in a total volume of 200 μL. BY4741 and W303 strains were tested against IP-1 in a 96-well plate, each containing 200 μL, incubated at 30°C and agitated using a Synergy MX (BioTek Instruments, Inc.). Cell growth was estimated by measuring optical density at 600 nm every hour for 24 h.

### Pheromone Signaling in the Presence of IP-1-CO-NH_2_ in *S. cerevisiae*

Strain BY4741 expressing the *FUS1-GFP* fusion was grown in 5 mL of synthetic complete media lacking histidine (SC-his) (yeast nitrogen base w/o amino acids 0.67%, potassium phosphate monobasic 0.1%, glucose 2%) containing 0.077% Complete Supplement Mixture (CSM-His) for 16 h at 30°C and agitation at 250 rpm. These cells were diluted with fresh medium to a final optical density of 0.4 at 600 nm and then 200 μL of cells was treated with either 10 μM α-pheromone for 2 h, 10 μM IP-1 for 2 h, or 40 μM IP-1-CO-NH_2_ for 8 h. The cells were then recovered and washed three times with sterile distilled water. Pellets were diluted in 20 μL of fresh medium and placed on a slide with 10 μL low melting point agarose and observed with a multiphotonic microscope (Olympus FluoView FV1000) at 60× magnification and an excitation wavelength of 480 nm and an emission wavelength of 510 nm. The images were reconstructed using the FV viewer software provided by the manufacturer of the microscope.

## Results

### Is Cell Cycle Arrest Associated With IP-1 Cytotoxicity in MATa Cells?

**FIGURE 2:**
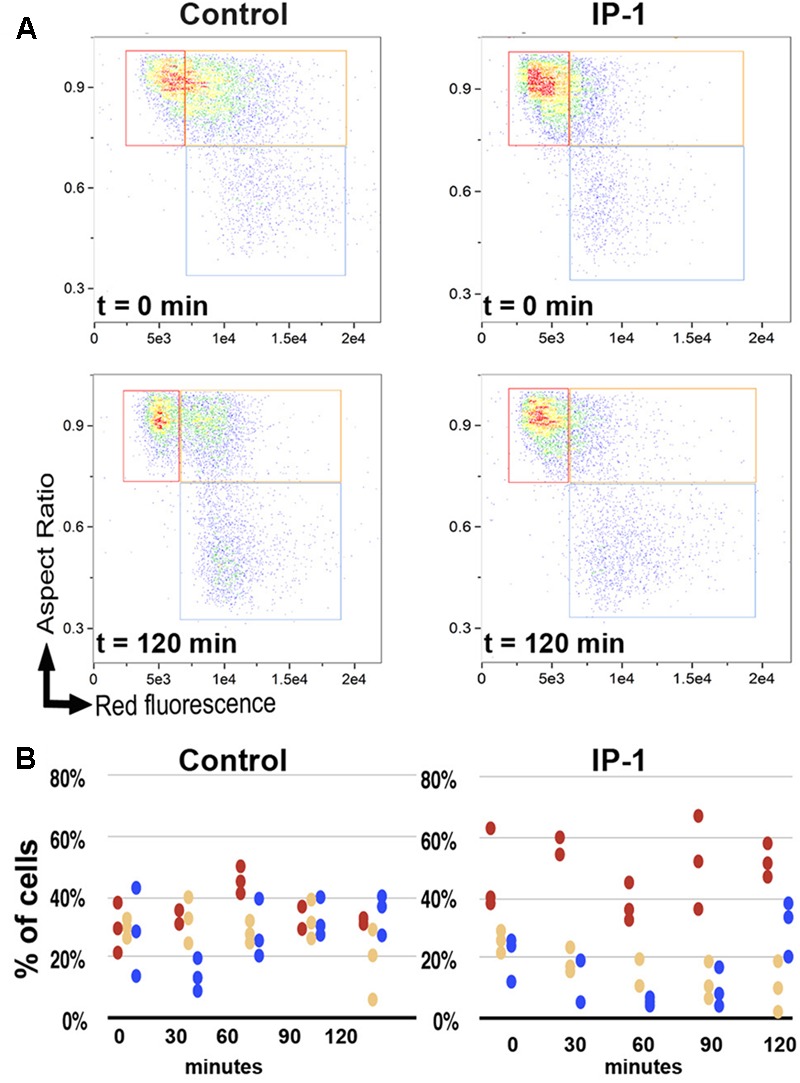
Cell cycle phases during IP-1-induced cell death. The cell cycle phase in BY4741 (MATa) cells in the exponential phase was analyzed in the presence of 10 μM IP-1 or distilled water as a control. Samples were taken every 30 min for 2 h. **(A)** Dot plot, aspect ratio of cells vs. fluorescence intensity of dead cells (see section “Materials and Methods”); this panel shows the distribution of cells in G_0_/G_1_ (red box), S (orange box), or G_2_/M (blue box) phases in one experiment in which cells were either exposed to IP-1 or not (control) at two different times (*t* = 0 min and *t* = 120 min). **(B)** Dispersion plot, a quantification (percentage, %) of cells observed in different cell cycle phases when exposed to IP-1 or not (control): red circles represent G_0_/G_1_ phase, yellow circles S phase, and blue circles G_2_/M phase; the panel presents the results of three independent experiments and each experiment recorded 15,000 events.

In a previous study, we showed that IP-1 alters mitochondrial function in isolated mitochondria, but we also noted that such activity alone or in combination with pheromone activity did not explain the cytotoxicity of IP-1 in MATa cells (Rodriguez Plaza et al., 2012). Here we have extended those observations and also measured the effect that IP-1 produces on MATa cell growth in respiratory and/or fermenting media. IP-1 inhibits the growth of cells in both respiratory and fermenting media (BY4741 cells grown in YPD media), yet cells grown in respiratory media are more resistant to IP-1 cytotoxicity (W303 cells grown in YPLac media) than those grown in fermenting media (BY4741ρ0 cells grown in YPD media) (see Supplementary Figure S1). Yeast cells grown in rich media in batch conditions tend to switch from respiratory to fermenting metabolism as a result of oxygen deprivation; hence, these cells are under conditions similar to those of cells grown in fermenting media. How are cells growing in fermenting media more susceptible to the cytotoxicity of IP-1? One possibility is that fermenting cells are prompted to enter into a state similar to that induced by nutrient deprivation, which leads to cell cycle arrest (Broach, 2012). This possibility is consistent with our previous results regarding the requirement of the STE2 receptor for IP-1 to inhibit cell growth (Rodriguez Plaza et al., 2012). That is, Ste2p signaling induces multiple responses in the cell, but relevant to this study is the arrest of the cell cycle in G_0_/G_1_, which is ultimately required for cells to mate (Torres et al., 2011). We will explore the possibility that IP-1 requires a cell cycle arrest to kill cells, but first we established a procedure to quantify cell death and cell cycle stage using cell cytometry coupled to fluorescence microscopy. First, to quantify the cell death achieved by IP-1 on MATa cells, we performed a viability assay using the FUN1 fluorophore (see Supplementary Figure S2). We observed that cells exposed to distilled water (the diluent for all peptides tested in this study) displayed defined red vacuolar structures, indicating that FUN1 was being metabolized and that consequently the cells were living. However, FUN1 was not metabolized in cells treated with IP-1 and a diffuse orange fluorescence was observed in the cytoplasm, indicating that these were dead cells. We observed a significant difference in the number of viable cells after 1 h of treatment with IP-1 compared to cells not exposed to this peptide (**Figure 1**); notably, the number of dead cells treated with IP-1 was similar to the number of dead cells when the cells were boiled (positive control for dead cells) confirming the effectiveness of IP-1 in the induction of cell death in MATa cells. Furthermore, we determined the cell cycle phase of the MATa cells treated with IP-1 at different times, and observed that these cells tended to accumulate in G_1_ phase (see **Figure 2**).

### Which Cellular Responses Activated by Pheromone Signaling Are Required for IP-1 to Kill MATa Cells?

**FIGURE 3:**
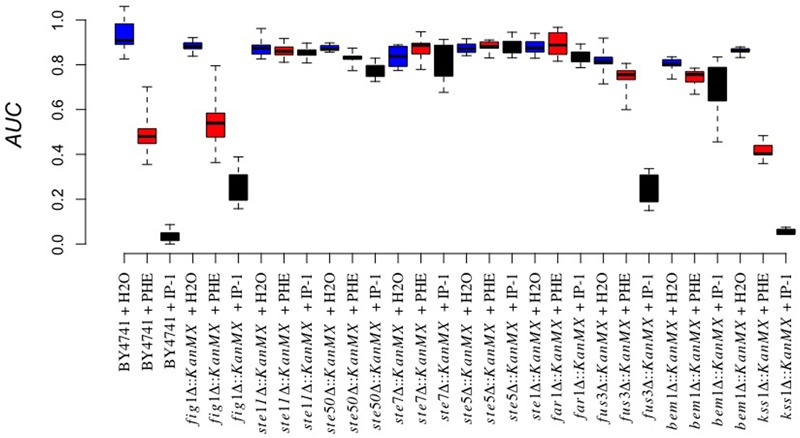
Involvement of the MAPK pathway in IP-1-induced growth inhibition. The cell growth of different BY4741 strains carrying a null mutation in genes involved in the MAPK pathway exposed or not to IP-1 was analyzed by recording the relative area under the growth curve (AUC); IP-1 was tested at 10 μM (black bars), using distilled water (blue bars), and α-pheromone 10 μM (red bars) as controls. Every gene substitution was performed in a BY4741 strain, and it is for this reason that the name of the strain is not indicated on the X-axis, to facilitate its visualization. The figure summarizes the results obtained from at least 12 experiments.

As noted above, Ste2 signaling involves multiple cellular responses. To discard the participation of cellular processes not involved in cell cycle arrest in the cell death induced by IP-1 on MATa cells, we analyzed the effect on cell growth that IP-1 has on MATa strains carrying a null mutant of genes involved in different cellular processes activated by pheromone signaling (e.g., autophagy, filamentous growth, calcium influx) and we found that none was relevant to IP-1-induced cell death (see Supplementary Figure S3). On the contrary, the null mutants of every gene involved in the MAPK pathway activated by Ste2p prevented IP-1-induced cell death (see **Figure 3**); this signaling pathway leads cells to a cell cycle arrest. Our results show that the strains carrying null mutants of genes *STE5, STE7, STE11*, or *STE50* are fully protected against the cytotoxicity of IP-1 and the cell growth arrest induced by the pheromone. The *FUS3* null mutant, which is downstream of the previous MAPKs, showed moderate resistance against IP-1 cytotoxicity and was resistant to the pheromone-induced arrest. The *FAR1* null mutant, a kinase downstream of Fus3p, was also resistant to both the IP-1 cytotoxicity and the growth arrest induced by the pheromone; hence, genes affecting the cell cycle arrest mediated by the MAPK became resistant to the cytotoxicity of IP-1. Other kinases associated with the MAPK pathway (*FIG1, BEM1, KSS1*) were tested and only *BEM1* protected against the cell growth inhibition induced by either IP-1 or α-pheromone, indicating that the lack of *BEM1* protects against IP-1 cytotoxicty by preventing the cell cycle arrest. Alternatively, the *FIG1* null mutant did show protection against IP-1 growth inhibition similar to that observed by the *FUS3* null mutant; however, the *FIG1* null mutant did not protect against the growth arrest induced by α-pheromone. Thus, *FIG1* is the only gene that protects against the cytotoxicity of IP-1 by a mechanism not related to the prevention of cell cycle arrest.

### Is Entry Into the G_1_ Cell Cycle Phase Sufficient for IP-1 Cytotoxicity?

**FIGURE 4:**
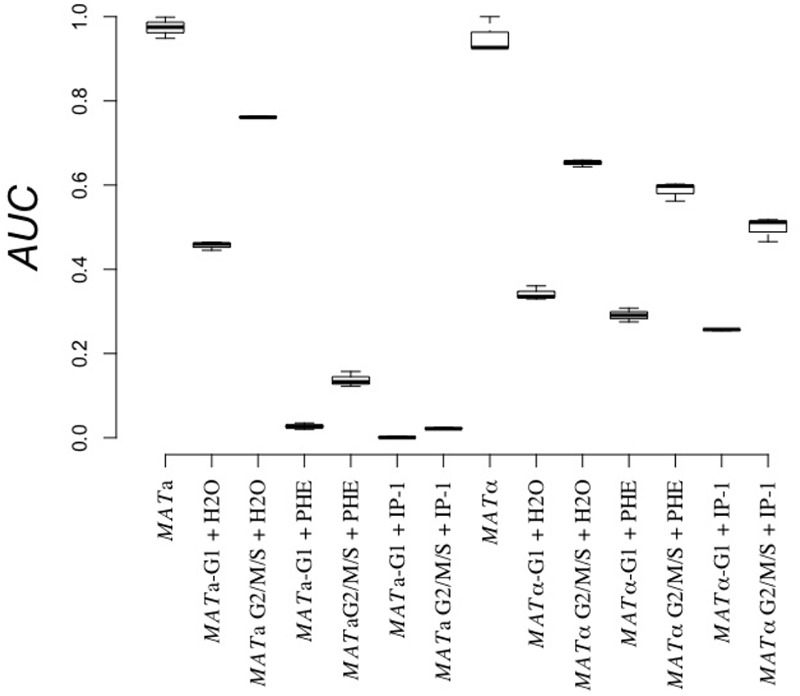
Cell growth effect of IP-1 on cells in G_1_ vs. other cell cycle phases. BY4741 (MATa) and BY4742 (MATα) cells were elutriated to separate cells in G_1_ phase from the rest (G2/M/S), then treated separately with 10 μM IP-1 (+IP-1), α-pheromone (+PHE), or distilled water as a control (+H_2_O). The relative area under the growth curve (AUC) after 24 h of growth is reported. The figure summarizes the results obtained from at least six experiments.

**FIGURE 5:**
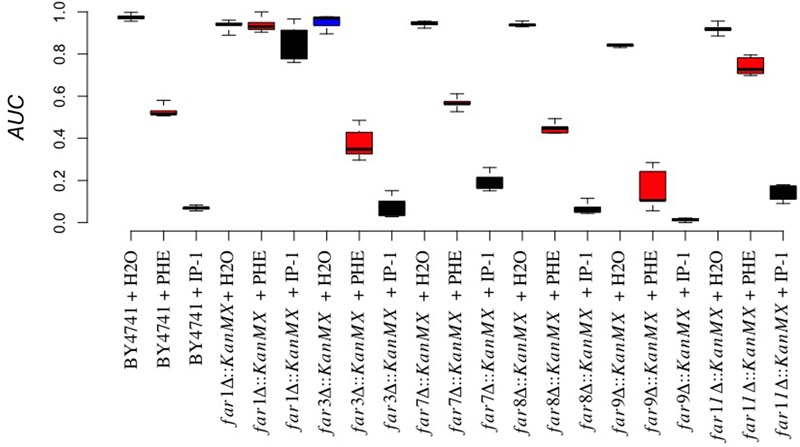
FAR null mutant effect on IP-1-induced growth inhibition. The cell growth of different strains carrying a null mutation in FAR genes (*FAR1, FAR3, FAR7, FAR8, FAR9, FAR11*) exposed or not exposed to IP-1 was analyzed by recording the relative area under the growth curve (AUC); IP-1 was tested at 10 μM (black bars), using distilled water (blue bars), and 10 μM α-pheromone (red bars) as controls. The figure summarizes the results obtained from at least 18 experiments.

To test whether or not entry into the G_1_ cell cycle phase (as opposed to arrest in G_0_/G_1_) is relevant in IP-1 cytotoxicity, we separated MATα cells in G_1_ phase from those in any other cell cycle phase (G_2_/M/S) by elutriation, and each fraction was tested against IP-1; MATa cells used as a positive control for IP-1 cytotoxicity did not grow when exposed to IP-1 independently of the cell cycle phase (see **Figure 4**). MATα cells (cells not expressing Ste2p) at different cell cycle phases treated with IP-1 were resistant to the IP-1-induced cell growth inhibition, hence showing that is not the G_1_ cell cycle phase, but the arrest on G_1_ (G_0_) the one relevant for IP-1 cytotixicty when cells activate the pheromone pathway. It should also be noted that the pheromone inhibited the growth of MATa cells in G_1_, but to a lesser extent than IP-1. Since the MAP kinases that protected against IP-1 cytotoxicity were kinases that act before the cell cycle arrest, we tested if kinases after the cell cycle arrest (*FAR3, FAR7, FAR8, FAR9, FAR11*; Kemp and Sprague, 2003) were also relevant for IP-1 cytotoxicty. All the strains carrying a null mutant on these genes showed cell growth arrest induced by the pheromone and cell growth inhibition induced by IP-1 (see **Figure 5**). Hence, induction of pheromone response leading to cell cycle arrest seems to be relevant in IP-1 cytotoxicity during the pheromone response.

### Can IP-1 Kill MATα Cells Arrested in G_0_/G_1_ Independently of *STE2*?

We previously reported that the pheromone receptor Ste2p is required for cell death induced by IP-1 in MATa cells, but our current results suggest that only cell cycle arrest induced by α-pheromone through Ste2p is relevant in IP-1-induced cell death. To determine whether cell death induced by IP-1 can be achieved independently of Ste2 activity, MATα cells (naturally not expressing *STE2*) carrying a null mutation on the gene coding for the kinase Hog1p (MATα:*hog1Δ::KanMX* which is hereafter referred to as MATα:*Δhog1*) were used to induce cell cycle arrest independently of Ste2p. To arrest the cell cycle of MATα:*Δhog1* cells, sodium chloride (NaCl) was added to the medium (see section “Materials and Methods”). After 30 h in YPD with 0.6 M NaCl, the MATα:*Δhog1* cells did not grow (see Supplementary Figure S4) and were in G_1_ phase (see Supplementary Figure S5). Cells were stained with FUN1 for the live/dead assay; we observed that 97% of the MATα:*Δhog1* cells treated with IP-1 were dead (see **Figure 1**). Furthermore, to test for the relevance of arresting the cell cycle in the IP-1-induced cell death, NaCl was washed away from the media and these cells were grown for 2 h in NaCl-free media. Then, the cells were exposed to IP-1 and no inhibition of growth was observed (see **Figure 1**). These results indicate that the cell cycle arrest independent of the Ste2p receptor was sufficient to kill cells in the presence of IP-1. Nevertheless, it has been reported that crosstalk exists between the stress response and the α-pheromone response at the MAPK pathway at the Ste11 and Ste50 kinases (Babazadeh et al., 2014), so these experiments did not rule out the participation of these MAP kinases in IP-1-induced cell death.

### Can IP-1 Induce Cell Death in Cells Arrested in Their Cell Cycle Independently of the Pheromone’s Pathway?

**FIGURE 6:**
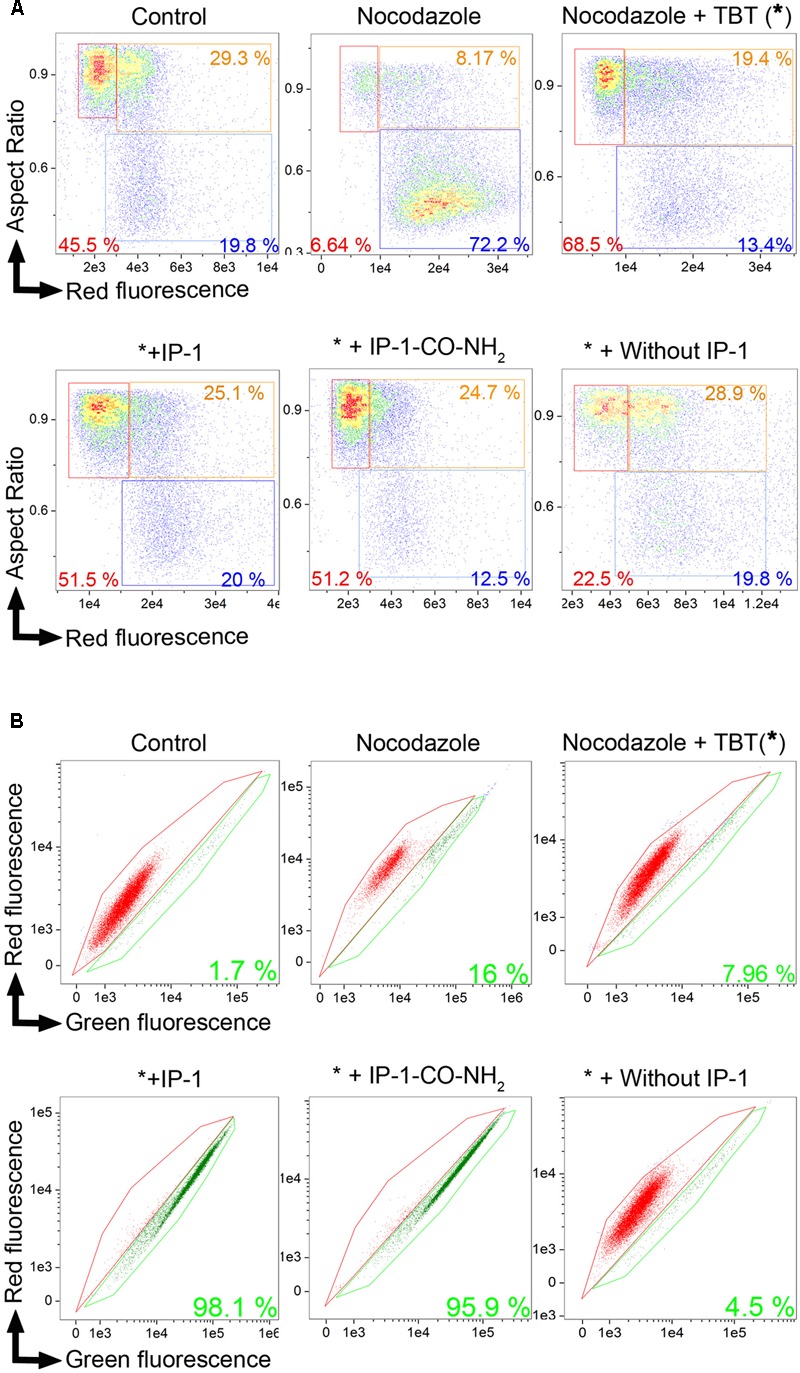
IP-1 induces cell death in MATα cells arrested chemically in G_1_/G_0_ phase. BY4742 (MATα) cells in the exponential phase were synchronized with nocodazole in M phase, then arrested with TBT in G_1_/G_0_ phase, and treated with 10 μM IP-1 or IP-1-CO-NH_2_. Cell cycle analysis and the FUN 1 assay were performed after each treatment. Treatment with nocodazole and TBT is represented by an asterisk (^∗^) for abbreviation. **(A)** Dot plot, cell aspect ratio vs. cell death fluorescence intensity, allowing for identification of cells in G_1_/G_0_ phase (red box), cells in S phase (orange box), or cells in G_2_/M phase (blue box); the number in each box refers to the percentage of each cell phase in 15,000 cells analyzed. **(B)** FUN1 cell viability assay. A bivariate plot of the intensity in red and green channels was generated from the in-focus population. Red area contains live cells while green area contains dead cells. The percentage of live cells among the 15,000 cells analyzed in each condition is indicated in the corresponding color. Only one experimental result is presented in this image, but these results were confirmed by a second independent experiment not shown.

To further study the dependence of the MAPK pathway on IP-1-induced cell death, we induced cell cycle arrest of MATα cells using cell cycle inhibitors that do not target the pheromone pathway, but do activate the MAPK pathway. Specifically, we used nocodazole to synchronize cells in the M phase and activate the MAPK pathway (Hayne et al., 2000) and tributyltin (TBT) to shift the cell cycle arrest induced by nocodazole to G_0_/G_1_ (Sekito et al., 2014) in MATα cells, which do not express the pheromone receptor (see section “Materials and Methods”). We validated the inhibition of growth of MATα cells treated with each cell cycle inhibitor and we included MATa cells in these experiments as positive controls for IP-1 cytotoxicity (see Supplementary Figure S6). The cell cycle phase was determined by flow cytometry coupled to fluorescence microscopy (see **Figure 6A**). MATα cells treated exclusively with nocodazole were not killed by IP-1 (see Supplementary Figure S6); only cells treated with nocodazole and TBT were susceptible to the toxic activity of IP-1 (see **Figure 6B**). These results suggest that arrest in G_0_/G_1_ is relevant in IP-1 cytotoxicity and that the MAPK pathway is only relevant in IP-1-induced cell death due to its participation in cell cycle arrest. Additionally, these experiments provided an explanation for the dependence of *STE2* on IP-1 cytotoxicity; that is, the pheromone pathway activated by *STE2* only seems to be relevant in IP-1-induced cell death due to its ability to arrest cells in their cell cycle.

### Is the Arrest of the Cell Cycle Without Signaling Sufficient for IP-1 to Induce Cell Death?

**FIGURE 7:**
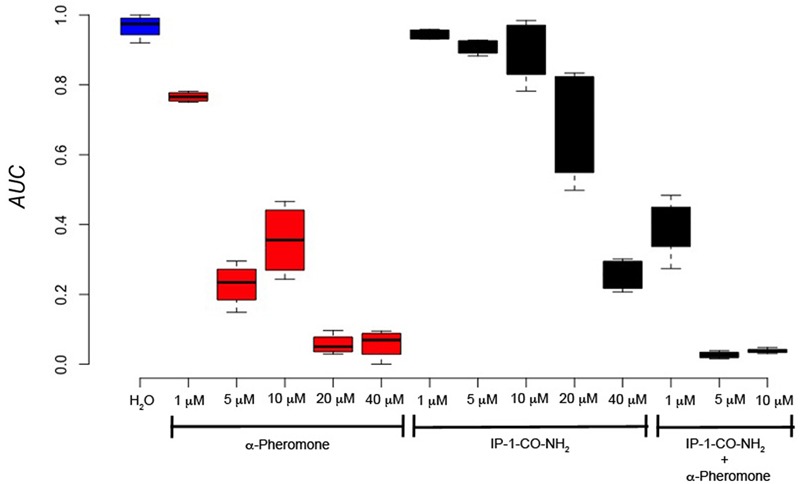
Cell growth of MATa cells is not inhibited by IP-1-CO-NH_2_ unless the pheromone pathway is activated. MATa cells were treated with different concentrations of IP-1-CO-NH_2_, α-pheromone, or an equimolar mix of both peptides. The image presents the relative area under the growth curve (AUC) of these cells after 24 h of growth. The figure summarizes the results obtained from at least four experiments.

**FIGURE 8:**
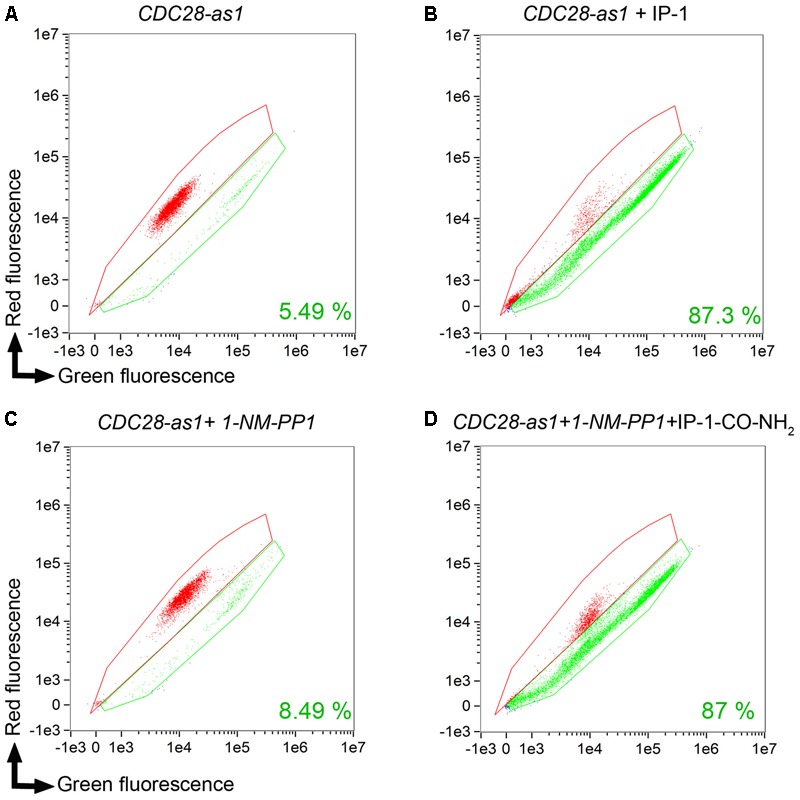
IP-1-CO-NH_2_ induces cell death only when cells are arrested in their cell cycle. The CDC28-as1 strain was used for cell cycle arrest with 10 μM NM-PPI CDC28 permeable inhibitor; staining cells with FUN1 was done in order to quantify the dead cells using an Amnis^®^ microscope-cell-cytometry station (see section “Materials and Methods”). Each panel presents the areas where live (green) and dead (red) cells are located by plotting the fluorescence of living cells (green fluorescence) against the dead cell fluorescence (red fluorescence). **(A)** CDC28-as1 viability control. **(B)** CDC28-as1 in the presence of IP-1 which is used as a control for the expected number of dead cells. **(C)** CDC28-as1 in the presence of CDC28p inhibitor NM-PPI. **(D)** CDC28-as1 in the presence of CDC28p inhibitor NM-PPI and IP-1-CO-NH_2_. Each panel presents the results of 15,000 events. Only one experimental result is shown in this image, but these results were confirmed by a second independent experiment not shown.

To test the idea that pheromone signaling is only relevant for death by IP-1 because of the arrest of the cell cycle, a variant of IP-1 was synthesized. It has been previously observed that the modification of the C-terminal end of α-pheromone significantly reduces its activity (Naider and Becker, 2004). To reproduce this effect in IP-1, a version of IP-1 modified at the C-terminus was synthesized: an amide group was incorporated into its C-terminus; we refer to this peptide as IP-1-CO-NH_2_ (see **Table 2**). We expected that the pheromone activity would be reduced or absent in this variant of the IP-1 peptide. Indeed, MATa cells exposed to 10 μM of IP-1-CO-NH_2_ grew normally, while the same cells treated with α-pheromone showed the typical delay in growth corresponding to a transitory arrest in their cell cycle. However, IP-1-CO-NH_2_ induced partial inhibition of growth of MATa cells at 40 μM (see **Figure 7**). Such inhibition of growth is not mediated by the activation of the pheromone pathway, as inferred from the expression and localization of *FUS1-GFP*, a marker of the pheromone’s pathway (see Supplementary Figure S7). Thus, we have generated a derivative of IP-1 that does not inhibit MATa cell growth at 10 μM as a result of its pheromone activity deficiency. Furthermore, combining IP-1-CO-NH_2_ with α-pheromone inhibited the growth of MATa cells to an extent similar to that seen with IP-1 (see **Figure 7**). This result confirms that IP-1-CO-NH_2_ fails to inhibit the growth of MATa cells due to its lack of pheromone activity and consequently its failure to induce cell cycle arrest. The requirement of cell cycle arrest to kill MATa cells was tested using IP-1-CO-NH_2_ in two different approaches. First, cell cycle arrest was induced with nocodazole and TBT, as previously described above, and we observed that IP-1-CO-NH_2_ killed MATa cells to a similar extent as IP-1 only when cells were arrested in G_0_/G_1_ phase (see **Figure 6**). Second, cell cycle arrest was induced in MATa:CDC28-as1 cells using a CDC28p kinase inhibitor, 1-NM-PP1 (Shokat et al., 2000); yeast cells are permeable to 1-NM-PP1, which inhibits CDC28p-as1, a variant of the catalytic subunit of the cyclin-dependent kinase (CDK) that controls the cell cycle in yeast. Thus, the use of 1-NM-PP1 should lead to an arrest in the cell cycle independently of any signaling. In this study, 1-NM-PP1 alone (10 μM) inhibited cell growth (see Supplementary Figure S8), but did not kill cells (see **Figures 8A,C**). Combining this CDC28p-as1 inhibitor with IP-1-CO-NH_2_ peptide killed as many cells as treatment with IP-1 (see **Figures 8B,D**). The present experiments indicate that cell cycle arrest is sufficient to induce MATa cell death by the pheromone-activity-deficient peptide IP-1-CO-NH_2_.

## Discussion

We previously described a new class of targeted antimicrobial peptides, IPs, for which a peptide ligand (α-pheromone) inserted into the antimicrobial peptide sequence provides specificity to the antimicrobial activity of these peptides (Rodriguez Plaza et al., 2012). Our initial studies showed that IP-1 completely prevented cell growth of MATa cells. Here, using a FUN1 assay, we showed that IP-1 induces cell death in MATa cells (see **Figure 1**). The proportion of cell death achieved by IP-1 (97%) was larger than that reported for cell death induced by the pheromone at physiological concentrations (6%). Live cells metabolize FUN1, but it has been observed that some quiescent cells metabolize this compound even when the colony-forming capacity has been clearly reduced (Allen et al., 2006). In contrast, our results with IP-1-treated MATa cells stained by FUN1 showed a direct correlation with cell viability (measured by the colony-forming assay; Rodriguez Plaza et al., 2012), indicating that FUN1 is an indicator of cell viability and not of quiescent cells in our experiments. The relevance of the cell cycle to this cell death is analyzed here using a combination of genetic and pharmacological approaches.

We previously observed that IP-1 can inhibit oxygen consumption in intact yeast cells as well in isolated mitochondria, but we argued that the anti-mitochondrial activity of IP-1 could not explain its anti-fungal activity since yeast cells are fermentative (Rodriguez Plaza et al., 2012). Here we provide evidence that IP-1 inhibits cell growth more efficiently in cells growing under fermentative conditions, a metabolic condition that facilitates cell cycle arrest (see Supplementary Figure S1). In this regard, the CDK *PHO85*, which regulates cell cycle progression in G_1_ in response to levels of nutrients (Huang et al., 2007), phosphorylates the cell cycle inhibitor Sic1p, contributing to Sic1p degradation and exit from G_1_ (Nishizawa et al., 1998). Sic1p inhibits only cyclin-CDK complexes containing B-type cyclins, which are relevant in the S phase of the cell cycle (Weinreich et al., 2001); thus, Sic1p activity prevents progression to the S phase of the cell cycle and contributes to arrest in the G_1_ phase of the cell cycle. Sic1p is also the target of Hog1p (Escoté et al., 2004), which is activated by different forms of stress. These previous observations indicate that the lack of nutrients (e.g., fermentation conditions) may inhibit Pho85p activity and consequently favor G_1_ cell cycle arrest if a proper external stimulus is present. In the absence of such stimuli, cells may remain in G_1_ by a mechanism other than cell cycle arrest induced by α-pheromone. Our previous results showed that cells lacking the receptor for α-pheromone (MATα cells) are not killed by IP-1 under fermentation conditions (Rodriguez Plaza et al., 2012), suggesting that induction of cell cycle arrest in G_0_/G_1_ is relevant to IP-1-induced cell death.

In line with this idea, we also observed that *STE2*-deficient MATα:*Δhog1* cells arrested in their cell cycle by salt stress were killed by IP-1 (see **Figure 1**). It has been observed that the pheromone pathway is repressed by salt stress in three different ways: it delays the expression of the pheromone-induced genes, it inhibits protein translation by Hog1p-dependent phosphorylation of Rck2p, and it dampens MAPK signaling by Hog1p-dependent phosphorylation of Ste50p (Nagiec and Dohlman, 2012). Since the phosphorylation of Rck2p and Ste50p is mediated by Hog1p, it is likely that in a MATα:*Δhog1* strain, salt stress would activate the MAPK signaling induced by the pheromone. Thus, MATα:*Δhog1* cells exposed to IP-1 under salt stress would die as a consequence of the activation of the MAPK pathway, which would lead to cell cycle arrest. In agreement with the relevance of the MAPK pathway in IP-1-induced cell death, it is our observation that the lack of *BEM1* protects against IP-1 activity. Bem1p is required for shmoo formation and connects the mating signals (interacts with Ste5p and Ste20p) to the machinery involved in promoting actin cytoskeleton changes by interacting with Cdc24p and Cdc42p (Leeuw et al., 1995). Furthermore, the lack of *BEM1* causes cells to lose their sensitivity to the pheromone-induced growth arrest (see **Figure 3**), which supports the notion that cell cycle arrest mediated by the MAPK pathway is relevant to IP-1-induced cell death in yeast cells. In contrast with these results, we observed that cells arrested using nocodazole, which activates the MAPK pathway, were not sensitive to the toxic activity of IP-1 (see Supplementary Figure S6); the shifting of the cell cycle arrest to G_0_/G_1_ using TBT after treatment with nocodazole made cells susceptible to IP-1-induced cell death (see **Figure 6**). These results suggest that the MAPK pathway is relevant in IP-1-induced cell death only because it induces the arrest of the cell cycle.

We describe a variant of IP-1 that is defective in pheromone signaling activity (IP-1-CO-NH_2_); this peptide was able to kill MATa cells that were induced into cell cycle arrest independently of the MAPK pathway using an inhibitor of a Cdc28p variant (see **Figure 8**). Furthermore, IP-1 did not affect cell growth in MATα cells that were induced to cell cycle arrest using nocodazole, further discarding the relevance of the MAPK pathway in IP-1-induced cell death. Intriguingly, MATa or MATα cells that were induced to cell cycle arrest in G_0_/G_1_ using nocodazole followed by TBT were killed by IP-1 or IP-1-CO-NH_2_, respectively. This cell cycle arrest does not depend on the pheromone pathway (because it was not induced by the pheromone), yet we cannot rule out the participation of some of the molecular machinery involved in the pheromone response.

Our previous results showed the non-apoptotic nature of IP-1-induced cell death and correspondingly we argued that IP-1 kills cells through a mechanism different from the apoptosis-like mechanism induced by the pheromone at physiological concentrations (Rodriguez Plaza et al., 2012). In the present study we provide further evidence for this difference by showing that IP-1-induced cell death in MATa cells does not depend on respiratory activity (see Supplementary Figure S1), which contrasts with the respiratory dependency reported for the slow cell death induced by the pheromone at non-physiological concentrations; however, we did observe some protection against the cell growth inhibition induced by IP-1 resulting from the lack of *FIG1* (see **Figure 3**), which also protected against the fast cell growth inhibition induced by the pheromone at non-physiological concentrations (Zhang et al., 2006). The calcium influx activity of *FIG1* is not required for the fast cell death induced by the pheromone and *FIG1* does not participate in the activation of the MAPK pathway; these results have led to the idea that Fig1p and Mpk1p are two independent branches of the pheromone response that are regulated by Fus3p, Far1p, Bni1p, Spa2p, Pea2p, Rvs161p, Fus2p, and Fus1p (Muller et al., 2003). Hence, under the physiological response to the pheromone, the lack of *FUS3* may prevent cell cycle arrest and consequently it would be expected to protect against the toxic activity of IP-1. However, our observation that the lack of *FUS3* protects against IP-1-induced cell death to the same degree as the lack of *FIG1* (see **Figure 3**) suggests that the protection achieved by *FUS3* is relevant for its role in activating *FIG1* and not for its role in promoting cell cycle arrest. The failure to protect against IP-1-induced cell death by the null mutants of genes relevant in the calcium influx activity of *FIG1* (see Supplementary Figure S3) that do not affect cell cycle arrest (*PEA2, RVS161, BNI1*) indicates that the role of *FIG1* in IP-1-induced cell death is not related to its activity on calcium influx. Furthermore, it has been argued that fast cell death mediated by *FIG1* occurs in less than 40% of the cell population exposed to the pheromone because *FIG1* is regulated by mechanisms that control cell wall integrity (Zhang et al., 2006), and consequently that this cell death may reflect the outcome of attempting late steps of the mating process in the absence of a mating partner. Our results show that 70% of cells exposed to the killing activity of IP-1 are rescued by the lack of *FIG1*, suggesting that IP-1 may be inducing cell death through a mechanism that escapes the regulation of the cell wall integrity, and consequently of the mating process. These results further confirm that IP-1 induces a cell death different from that mediated by the pheromone at physiological concentrations, and consequently *FIG1* may have a different activity during this cell death than the observed during the pheromone-induced cell death.

**FIGURE 9:**
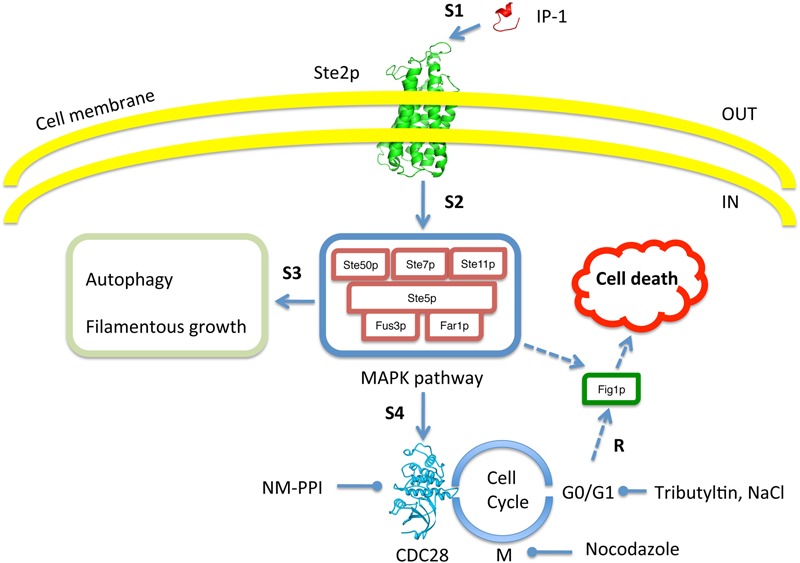
Schematic summary to test for dependence of IP-1 toxicity on cell cycle arrest. The image shows every aspect of the experimental system that was tested in our work. Our experimental results (R) show that IP-1 induces cell death only when cells are arrested in their cell cycle. To test this, the pheromone pathway was affected at different steps (e.g., S1, S2, etc.). For instance, IP-1 (red structure) was altered in its ability to bind Ste2p (green structure) and reduce the pheromone-like activity of IP-1 (indicated by S1). Also, different molecules were used to induce cell cycle arrest in G_0_/G_1_ or M phases: IP-1, α-pheromone, NaCl, NM-PPI, or nocodazole. Gene null mutants in the MAPK pathway (e.g., *STE50, STE7*) and other cellular programs induced by the pheromone (autophagy, filamentous growth) were tested for their participation in IP-1-induced cell death. The image indicates the inside and outside of the cell as well as the cell membrane. Broken arrow lines represent putative relationships of *FIG1* that may be relevant in IP-1-induced cell death.

In short, we analyzed yeast cell death in response to the presence of IP-1 after altering different steps of the pheromone response pathway and of the cell cycle (see **Figure 9**). **Figure 9** places *FIG1* as part of IP-1-induced cell death. *FIG1* codes for a protein with four transmembrane regions that shares a similar motif with the PMP-22/EMP/MP20/Claudin superfamily. The cell death activity of *FIG1* has been functionally related to a member of this superfamily, p53 apoptosis effector related to PMP-22 (PERP), which, like many other PMP-22 tetraspan proteins, upon its over-expression in mammalian cells induces apoptosis (Ihrie and Attardi, 2004); this may explain the loss of PERP in the more aggressive sparsely granulated pituitary adenomas (Kiseljak-Vassiliades et al., 2017) and its transcriptional activation by p53 during apoptosis in mammalian cells (Davies et al., 2009); more recently PERP has been observed to participate in cellular inflammation during *Salmonella* infection (Hallstrom and McCormick, 2016). *FIG1* overexpression also leads to a reduction in cell growth in yeast (Yoshikawa et al., 2011). Further studies would be useful in the elucidation of the role of *FIG1* in cell death induced in cell cycle arrest and the possible conservation of the mechanism for cell death induction in yeast and mammalian cells.

The relationship between cell death and cell cycle arrest has been described elsewhere (Wiman and Zhivotovsky, 2017) and in yeast it has been proposed that there must be cellular programs to induce cell death (Severin and Hyman, 2002), yet it is not clear how cells commit to a cell death program, especially when they are in an arrested stage of the cell cycle that is intended to protect them (Gray et al., 2004; Valcourt et al., 2012). The effectiveness of IP-1 in killing MATa cells (compared to that observed with α-pheromone, for instance), the genetic mutants, and the IP-1 variant described in this work makes this experimental system useful in the study of the mechanisms at play in the communication between cell cycle arrest and cell death. The relevance of this relationship between cell cycle arrest and cell death in yeast may have an impact on other organisms, considering the conservation of the cell cycle mechanism. For instance, cytotoxic molecules targeting cell cycle regulation have been recognized as potential therapeutics for treatment of different cancers (Waldman et al., 1997). A more recent controversy regarding the mechanism leading to the recurrence of cancer highlighted the need for development of new pharmaceuticals targeting dormant (arrested) cells as a method for treating resilient cancers (Wells et al., 2013). In this sense, molecules capable of inducing death of cells in cell cycle arrest show promise in cancer therapy (Gupta et al., 2009; Ehrhardt et al., 2013). Thus, polypharmacological compounds combining both cell cycle arrest and cell death activities may be desirable. Hunter-killer peptides, composed of a ligand peptide (hunter) fused to an antibacterial/antimitochondrial peptide (killer), represent one class of polypharmacological compounds useful in cancer treatment (Arap et al., 1998; Ruoslahti et al., 1999; Ellerby et al., 2008). In this regard, IP-1 is a special type of hunter-killer peptide that combines (1) a hunter peptide sequence that specifically binds to a receptor on the target cells to induce cell cycle arrest, and (2) a killer peptide sequence that is internalized through the receptor of the hunter peptide (Rodriguez Plaza et al., 2014). Our current results show that the signaling of the hunter peptide (pheromone) leading to cell cycle arrest is relevant in the killing induced by the killer peptide. Recognizing that resilient cancer cells (also referred to as metastatic dormant cells) may require the use of drugs that target cell cycle control, it would be convenient to add an activity related to cell cycle control to the design of hunter-killer peptides, as in the case of IP-1.

In summary, we have described peptides capable of killing yeast cells only when the latter enter cell cycle arrest. Such peptides may be useful in the study of the mechanisms relevant to yeast cell death when the yeast cells undergo cell cycle arrest.

## Author Contributions

VA, PMG, JRP, MLO, GS, EK, and GDR conceived and designed the experiments. VA, PMG, JRP, MLO, and GS performed the experiments. VA, PMG, JRP, and GDR analyzed the data. GDR, RV, and EK contributed reagents, materials, and analysis tools. VA, PMG, JRP, GS, RV, EK, and GDR wrote the paper.

## Conflict of Interest Statement

The authors declare that the research was conducted in the absence of any commercial or financial relationships that could be construed as a potential conflict of interest.
