# Supplementary material for: An Antimicrobial Peptide Induces FIG1-Dependent Cell Death During Cell Cycle Arrest in Yeast
Source: Front Microbiol. 2018 Jun 14;9:1240. doi: 10.3389/fmicb.2018.01240 (PMC6010521; doi:10.3389/fmicb.2018.01240)
Supplement: Supplementary file 1 [file Data_Sheet_1.DOC]

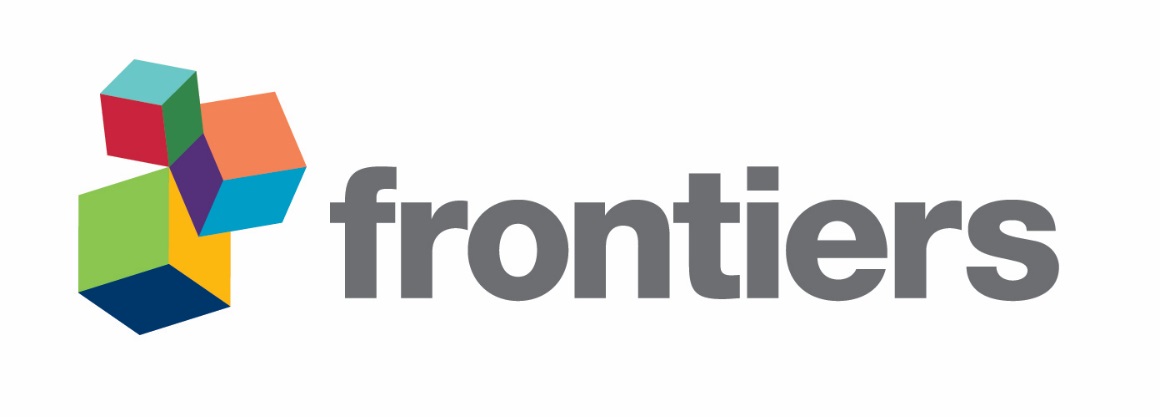

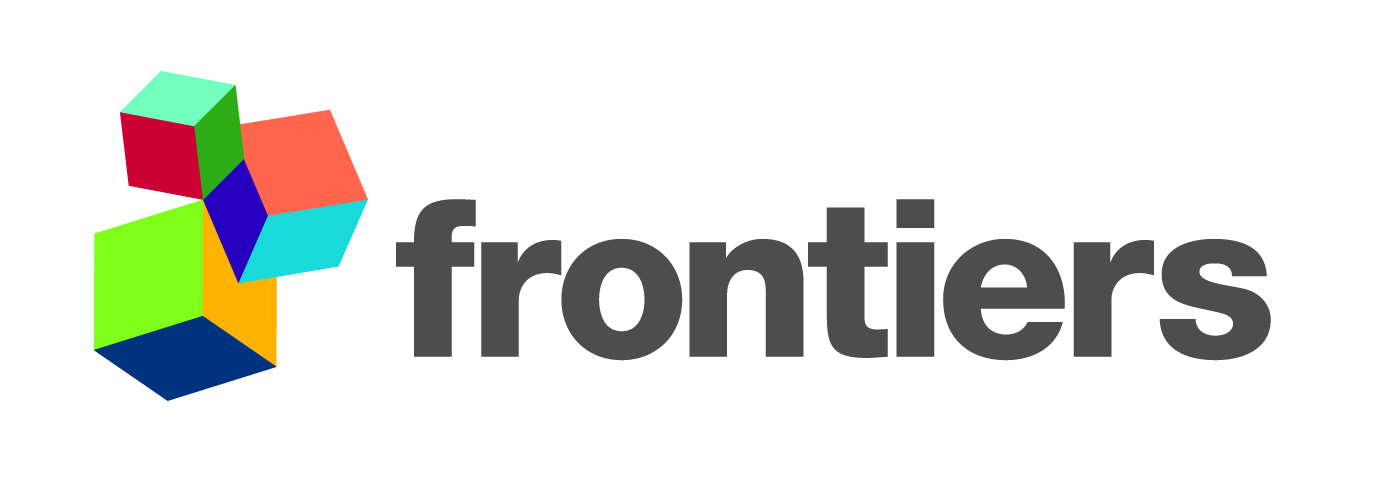
Supplementary Material

An antimicrobial peptide induces cell death dependent on cell-cycle arrest in yeast

Vladimir Juarez Arellano, Paula Martinell García, Jonathan G. Rodriguez Plaza, Maria Teresa Lara Ortiz, Gabriele Schreiber, Rudolf Volkmer, Edda Klipp, Gabriel Del Rio1*

*** Correspondence:** Corresponding Author: gdelrio@ifc.unam.mx

# Supplementary Data

Supplementary Material should be uploaded separately on submission. Please include any supplementary data, figures and/or tables.

Supplementary material is not typeset so please ensure that all information is clearly presented, the appropriate caption is included in the file and not in the manuscript, and that the style conforms to the rest of the article.

# Supplementary Figures and Tables

## Supplementary Figures


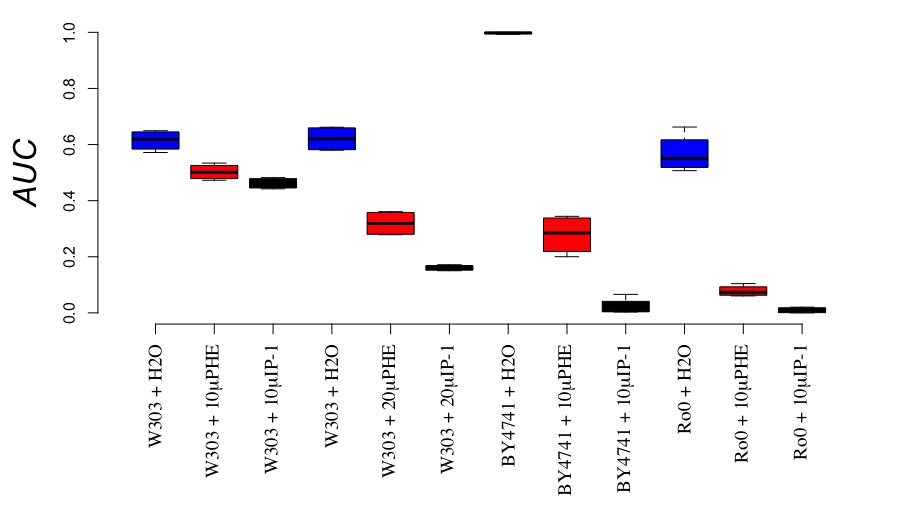


**Supplemental Figure S1. IP-1 effect on cell growth in respiratory/fermenting media.**

The relative area under the growth curve (AUC) of W303 cells grown in YPLac (respiratory metabolism mainly; BY4741 cells did not grow well in this media), BY4741 grown in YPD (fermenting and respiratory metabolism) and BY4741ρ0 (Ro0) cells grown in YPD (fermenting mainly) exposed to the pheromone (PHE; red boxes) or the IP-1 (black boxes) are presented; cells not exposed to any peptide but supplemented with equal volume of water (H2O; blue boxes) are presented as controls. The figure summarizes the results obtained from at least 4 experiments.


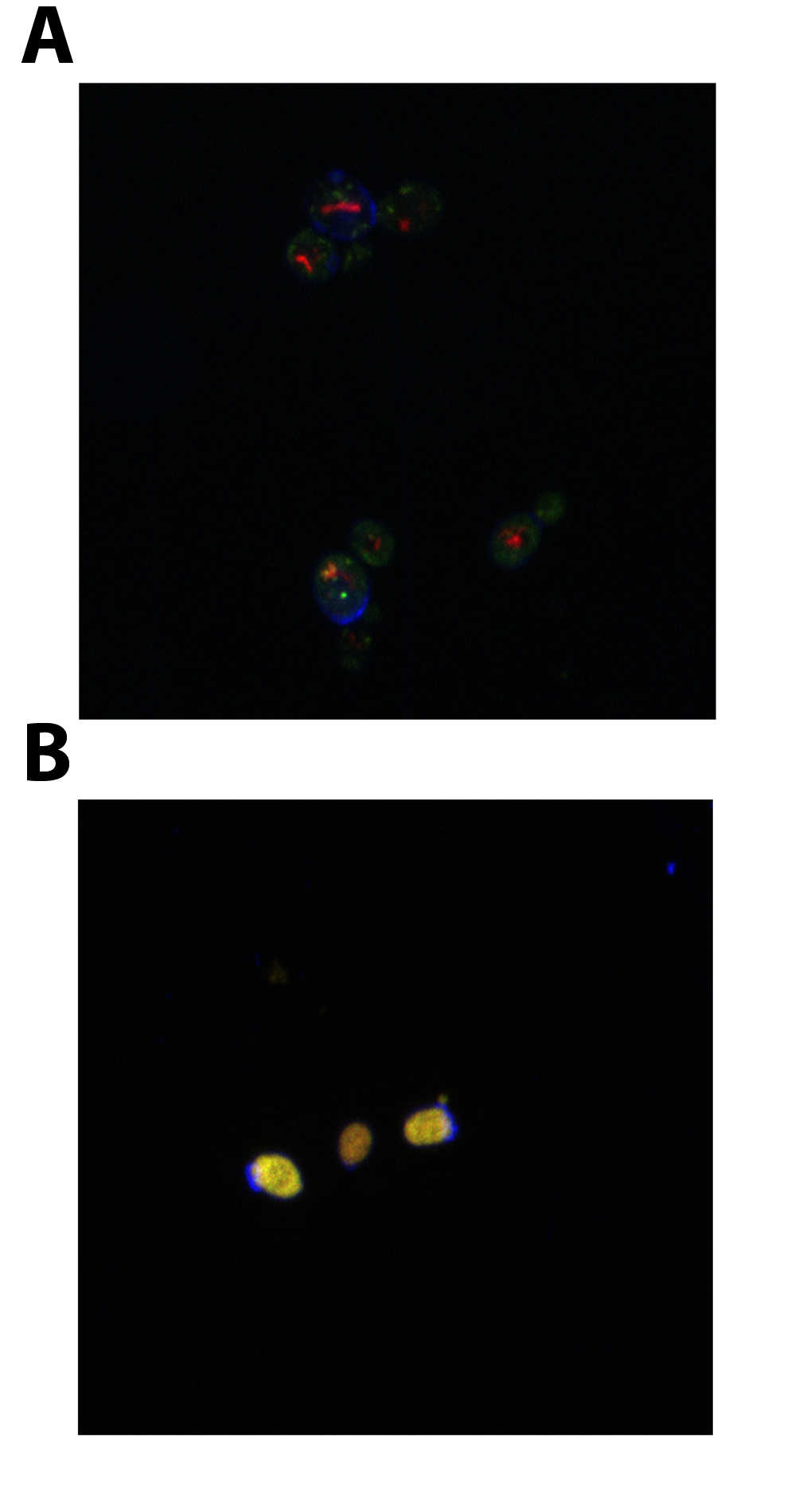


**Supplemental Figure S2. FUN-1 staining of BY4741 treated with IP-1.** BY4741 strain (MATa) cells were observed through fluorescence microscopy following staining with FUN1 (5μM) and Calcoflour White™ (7.5μM) for 30 minutes at 30°C. (**A**) Alive cells are identified by the red fluorescent intravacuolar structures (**B**) Dead cells that were tested at concentration 10μM of IP-1 exhibit extremely bright, diffuse, green-yellow fluorescence. Images were generated using a confocal inverted microscope 3I Marianas with spinning disk using a magnification of 63X/1.4.


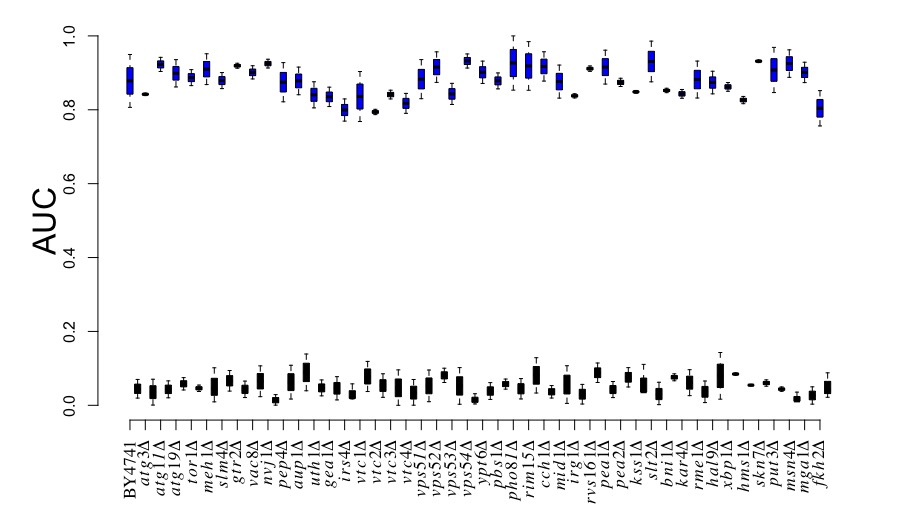


**Supplemental Figure S3. Testing for the dependence of cellular processes activated by the pheromone pathway in the cell growth inhibition induced by IP-1.** The relative area under the growth curve (AUC) of strains exposed to 10 μM of IP-1 (blue box) or not (black box) carrying a null mutant (the gene locus was substituted by *KanMX*) of genes involved in autophagy (*ATG3, ATG11, ATG19, TOR1, MEH1, SLM4, GTR2, VAC8, NVJ1, PEP4, AUP1, UTH1, GEA1, IRS4, VTC1-VTC4,VPS51-VPS54, YPT6, SLT2*), filamentous growth (*PBS1, PHO81, RIM15, CCH1, IRG1, RVS161, PEA1, PEA2, KSS1, BNI1, KAR4, RME1, XBP1, HMS1, SKN7, MGA1*), calcium flux control (*MID1, HAL9*) or other related to cell cycle control (*PUT3, MSN4, FKH2*); for brevity, gene substitutions in X axis are represented with a delta symbol (e.g., *atg3Δ*). The AUCs are shown for 24h. The figure summarizes the results obtained from at least 12 experiments.


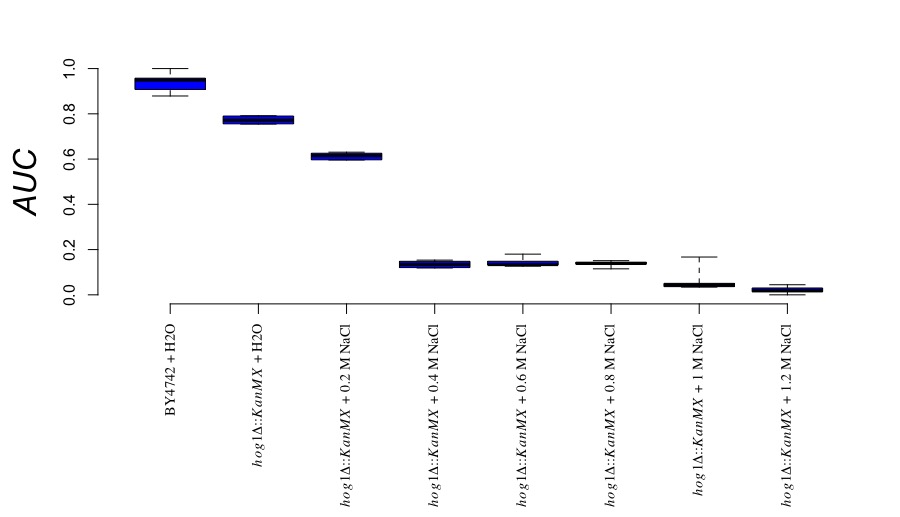


**Supplemental Figure S4. Growth of MATα:*hog1Δ::KanMX* treated with different NaCl concentrations.** The relative area under the growth curve (AUC) of Δhog1 (MATα:*hog1Δ::kanMX* are represented as *Δhog1* to abbreviate) exposed to different concentrations of NaCl (0 M, 0.2 M, 0.4 M, 0.8 M, 1 M, 1.2M) is reported. BY4742 (WT) was used as control. The label H2O is presented to indicate the absence of NaCl in the media. The figure summarizes the results obtained from at least 6 experiments.


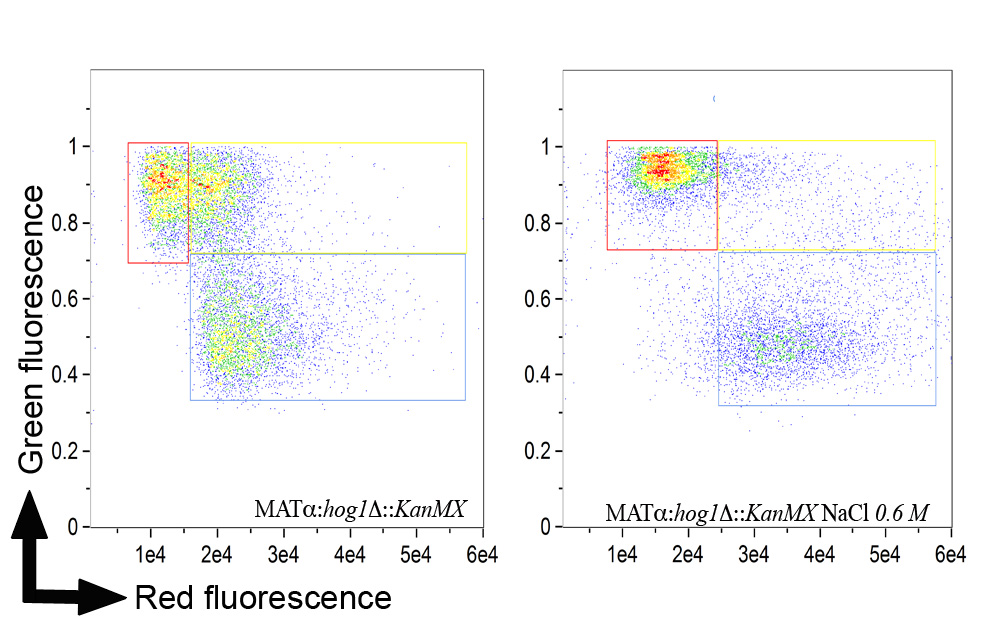


**Supplemental Figure S5. Cell cycle assay in Matα:*hog1Δ::KanMX* arrest in hyperosmotic stress.** Cells were exposed to 0.6 M NaCl (right panel) or not (left panel) and grown for 36 hours in YPD; afterwards, these cells were stained with propidium iodine (PI) and analyzed by fluorescent microscopy coupled to a flow cytometer (AMNIS) to determine the cell cycle stage for 15,000 cells. A bivariate plot of the aspect ratio and fluorescence intensity from the PI fluorecence was generated from the in-focus population. From the gathered images, G1, S or G2/M cells were visually identified. Cells treated with NaCl 0.6 M (left panel) were more frequently found in G0/G1 stage of cell cycle.


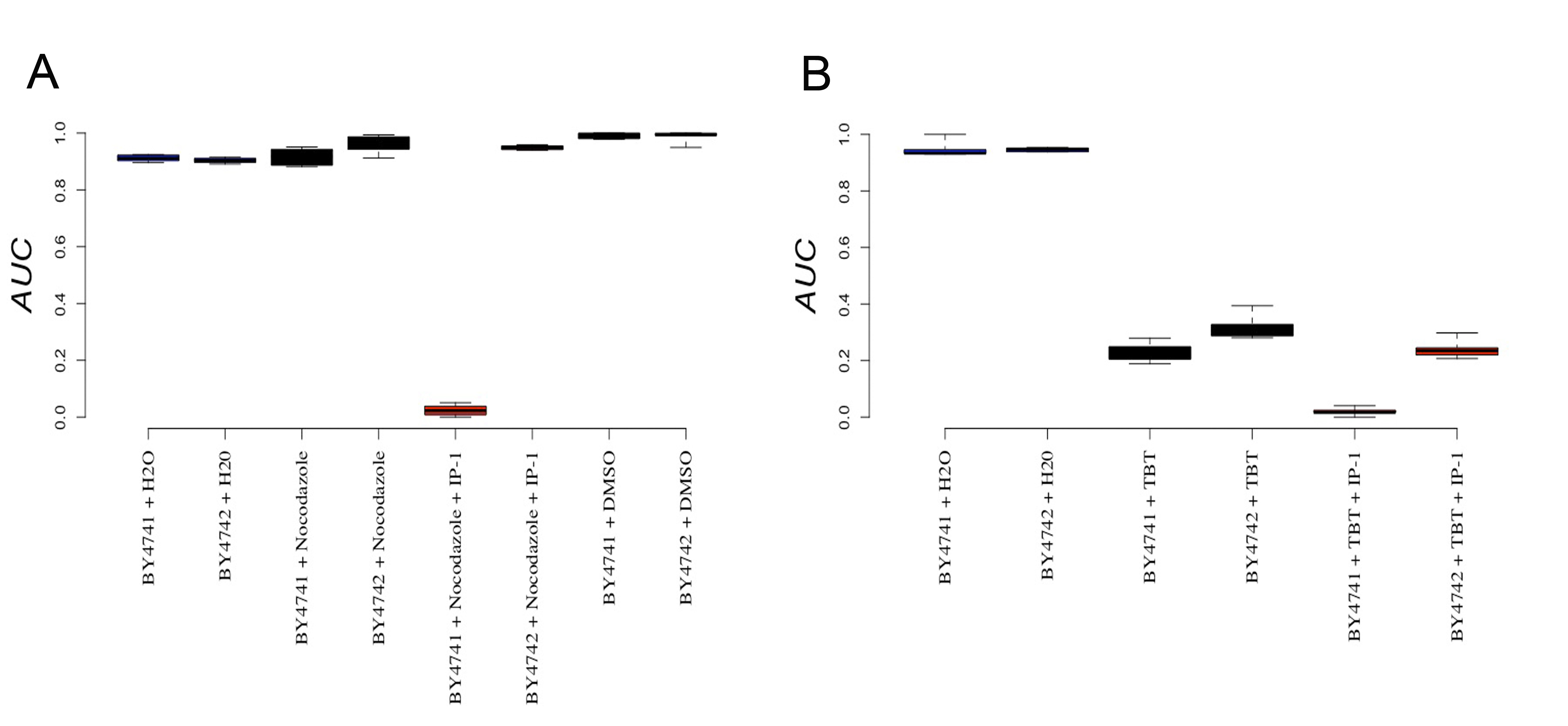


**Supplemental Figure S6. Growth inhibition by IP-1 in cell-cycle arrested cells.** BY4741 (MATa) and BY4742 (MATα) cells in exponential phase were treated with : **A)** nocodazole or **B)** TBT; both cases are represented as black boxes. In any case, cells were then treated with IP-1 (red bars); as controls cell were exposed to distilled water (H2O, blue bars) or DMSO (blue bars) the diluents for IP-1 or nocodozale, respectively. These cultures were grown in rich media (YPD) for 24 h and the relative area under the growth curve (AUC) is presented. For details on the preparation of these samples please review the Methods section.


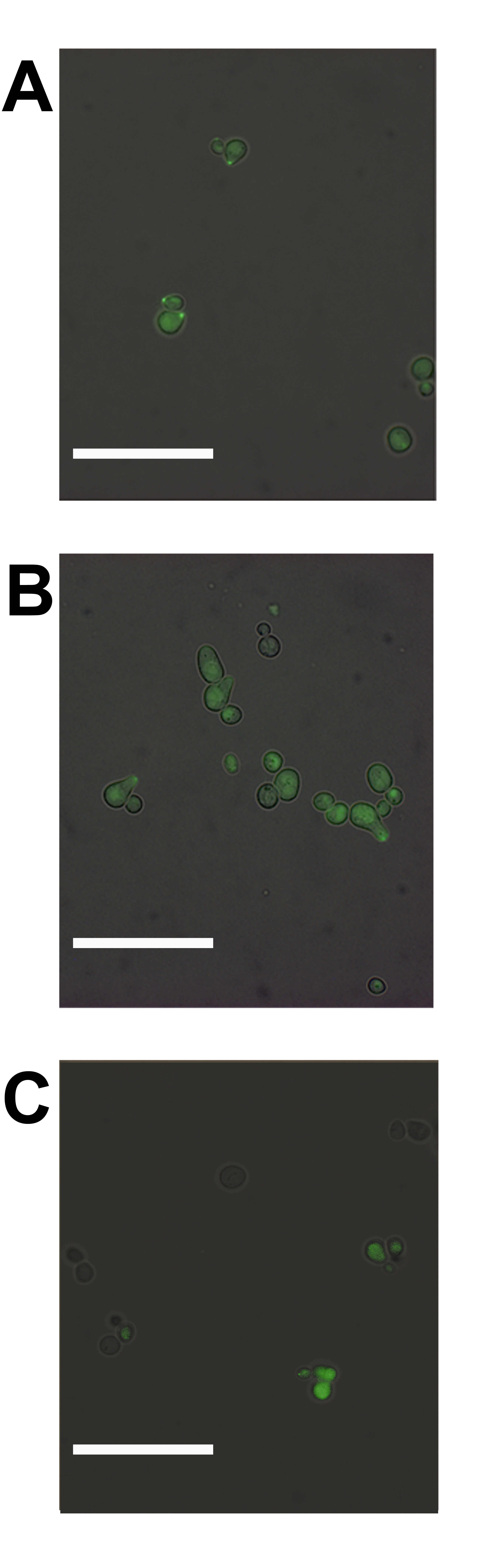
**Supplemental Figure S7. IP-1-CO-NH_2_ does not induce the pheromone pathway.** The activation of the pheromone pathway was detected by the expression of FUS1p; to detect in vivo the expression of FUS1p, its gene was fused to GFP, so that cells activating the pheromone pathway will display a distinct green fluorescence at the budding tip. **A**) MATa cells treated with 10μM α-pheromone show bud formation with FUS1p-GFP at its tip. **B**) MATa cells treated with 10μM IP-1 show budding and FUS1p-GFP fluorescence at their tip. Due to the cell death induced by IP-1, fewer cells present fluorescence at their tip; their anomalous shapes in the image identify death cells. **C**) MATa cells treated with 10 μM IP-1-CO-NH_2_ do not bud nor present the FUS1p-GFP characteristic fluorescence at the bud's tip even after 8 hours of treatment; the few cells displaying fluorescence in this panel most likely are dead cells as inferred from the anomalous shapes of these cells. The white bar's length is 50μm.


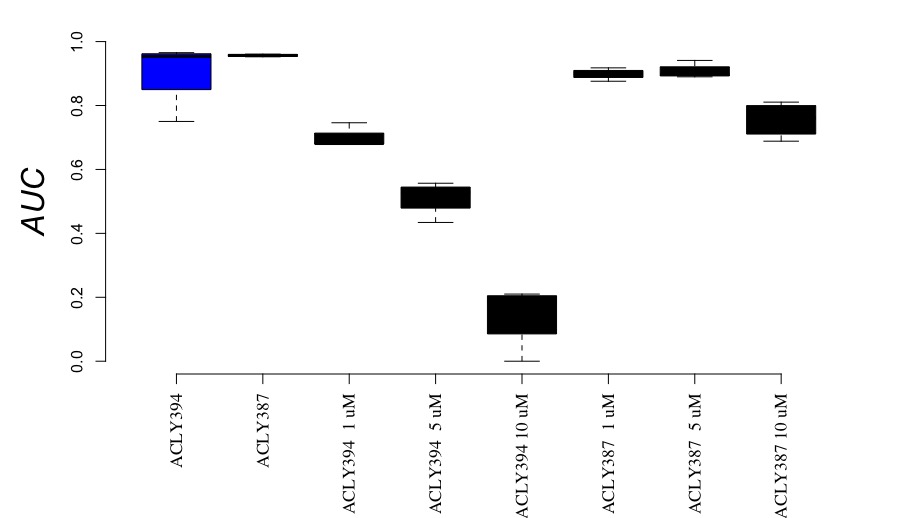


**Supplemental Figure S8. MATa:CDC28-as1 cell growth inhibition induced by NM-PP1.** W303 MATa cells expressing a YFP (ACLY387) was the control strain that contained the wild type copy of CDC28; ACLY394 strain was substituted the CDC28 gene coding for the catalytic MAP kinase domain by a mutant (CDC28-as1) that is inhibited by a permeable chemical compound, NM-PP1, were grown in rich media (YPD) for 24 h and exposed to different concentrations of this inhibitor (1, 5, 10 μM). The relative area under the growth curve (AUC) of these cells is presented as a measure to estimate the extent of cell growth inhibition. The figure summarizes the results of 4 independent experiments.
