# Supplementary figures and images for: An Antimicrobial Peptide Induces FIG1-Dependent Cell Death During Cell Cycle Arrest in Yeast
Source: Front Microbiol. 2018 Jun 14;9:1240. doi: 10.3389/fmicb.2018.01240 (PMC6010521; doi:10.3389/fmicb.2018.01240)

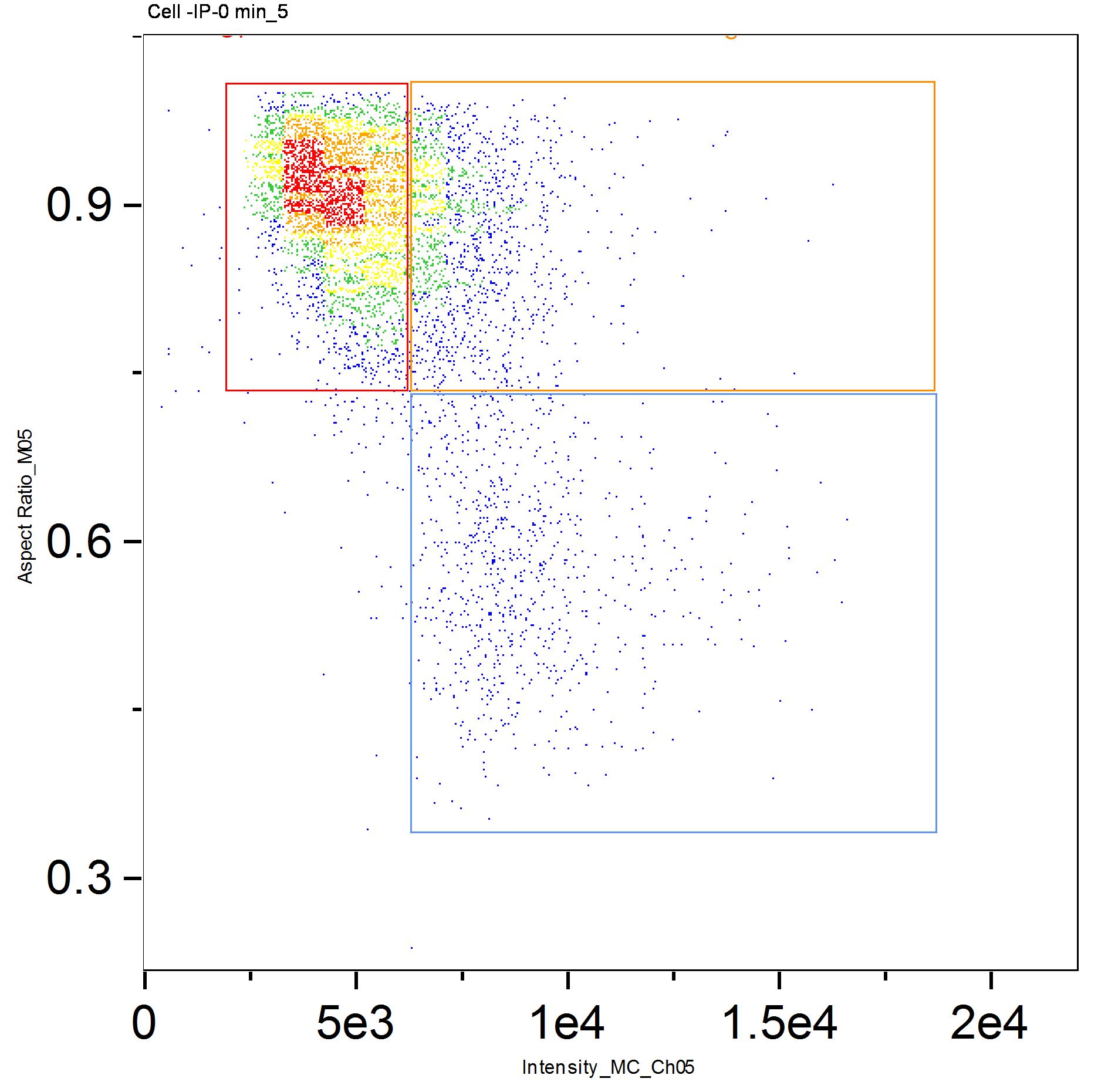

Supplement: Supplementary file 2 [file Data_Sheet_2.ZIP › Original composite images/Figure2/IP-0.png]

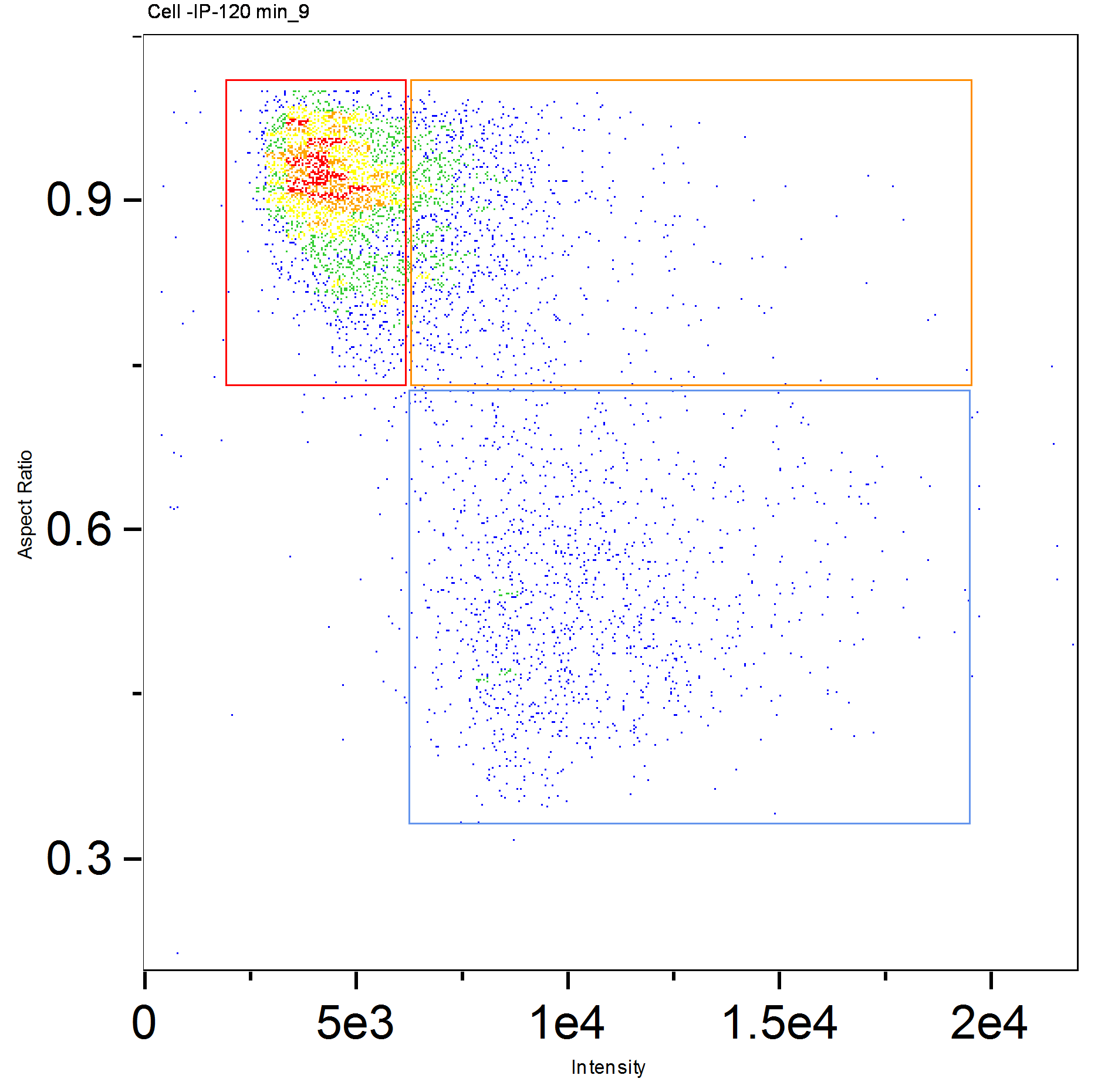

Supplement: Supplementary file 2 [file Data_Sheet_2.ZIP › Original composite images/Figure2/IP-120.png]

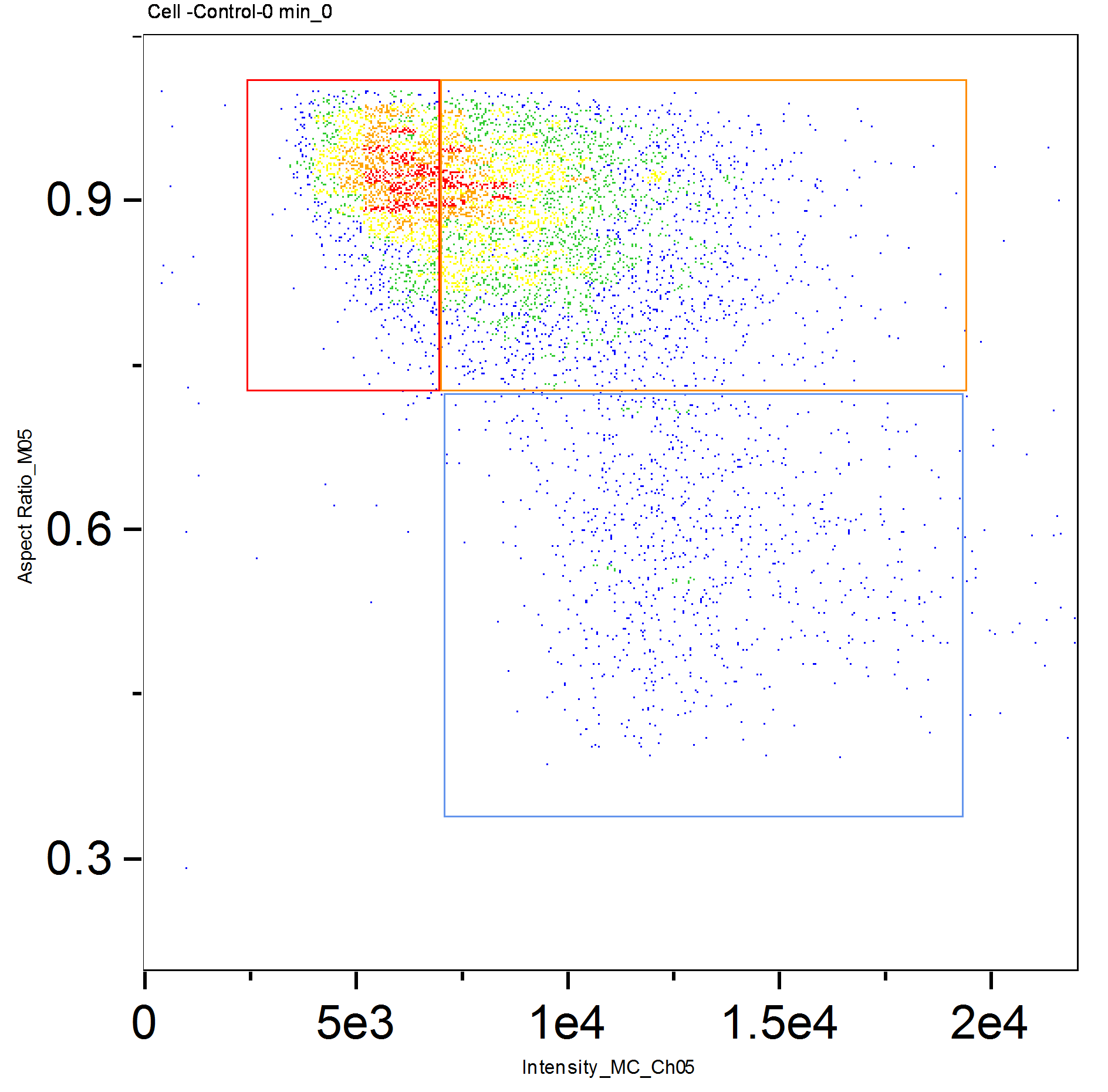

Supplement: Supplementary file 2 [file Data_Sheet_2.ZIP › Original composite images/Figure2/C-0 min.png]

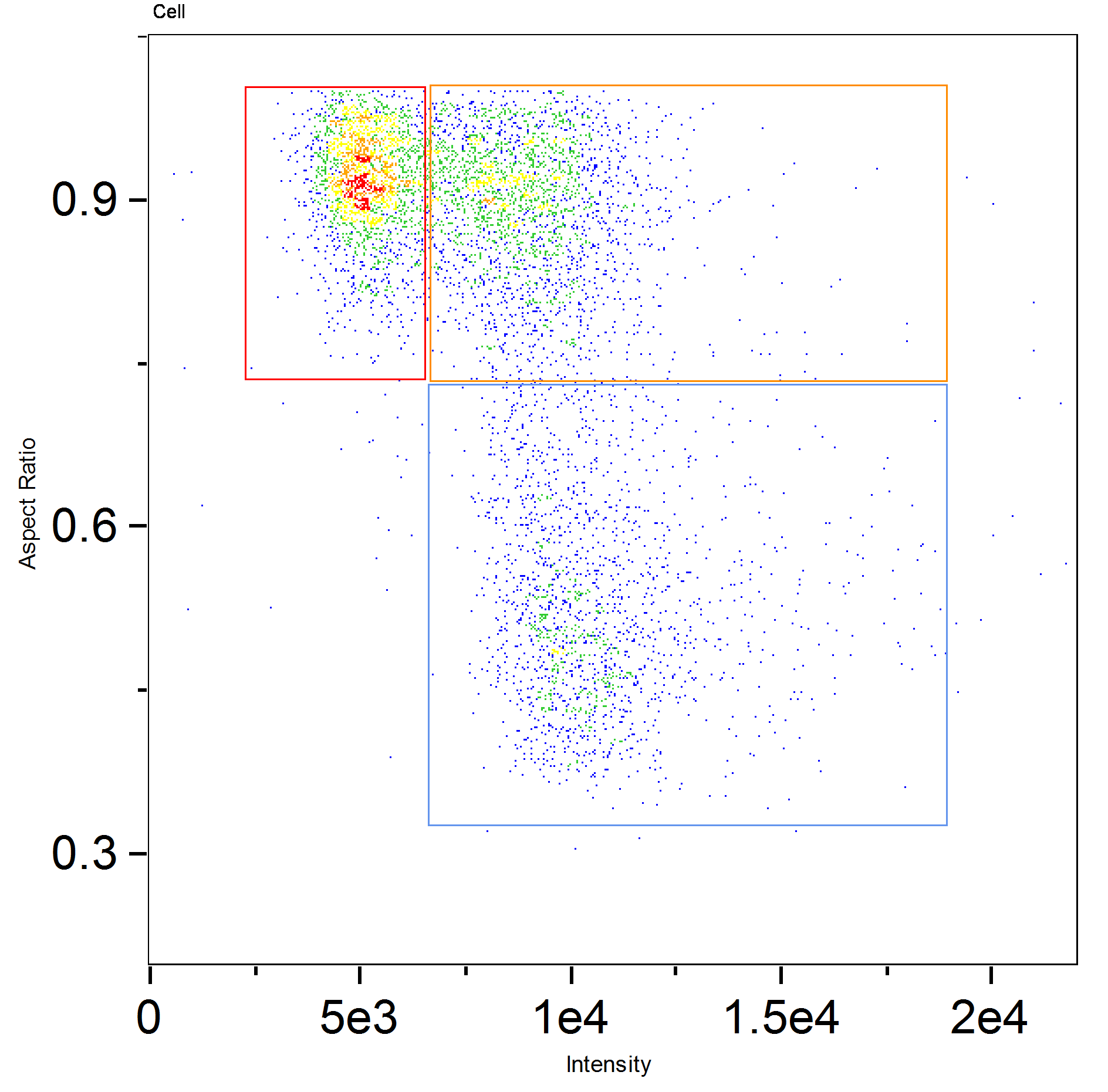

Supplement: Supplementary file 2 [file Data_Sheet_2.ZIP › Original composite images/Figure2/C-120 min.png]

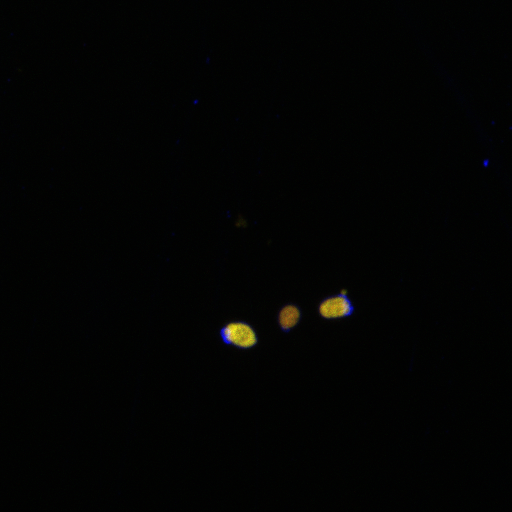

Supplement: Supplementary file 2 [file Data_Sheet_2.ZIP › Original composite images/FigureS2/B) Dead Cell.png]

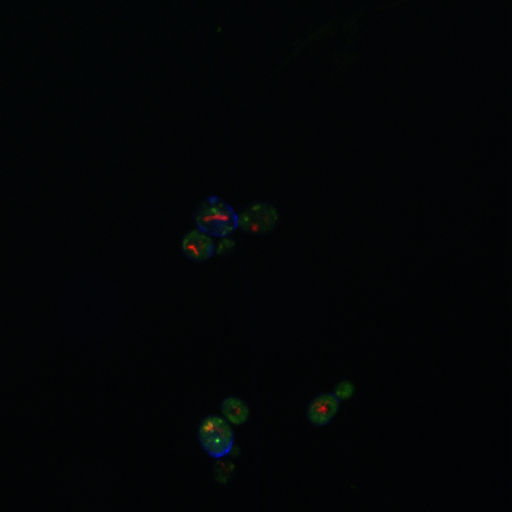

Supplement: Supplementary file 2 [file Data_Sheet_2.ZIP › Original composite images/FigureS2/A) Live Cell.png]

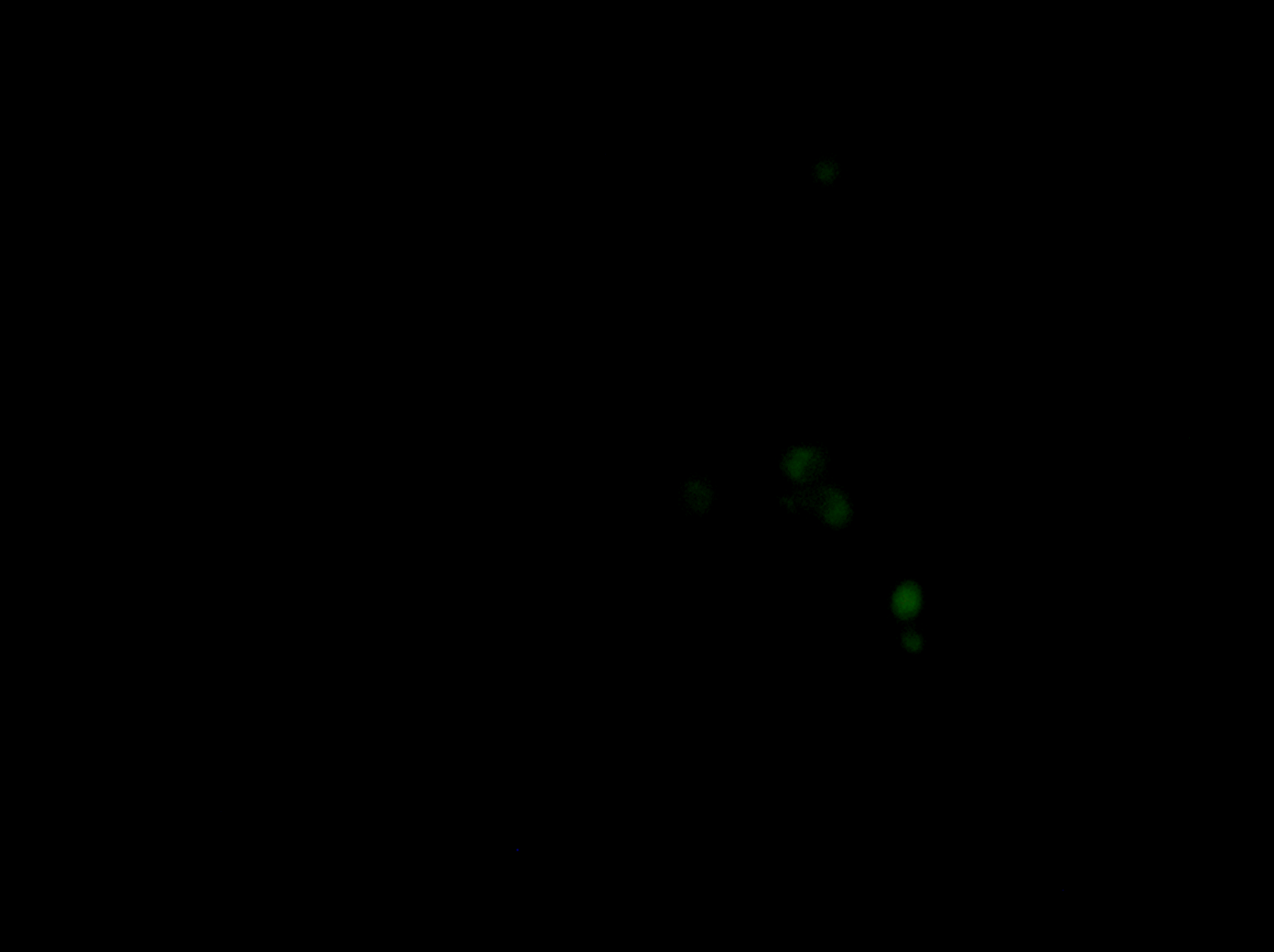

Supplement: Supplementary file 2 [file Data_Sheet_2.ZIP › Original composite images/FigureS5/C) PI-1-CO-NH2.tif]

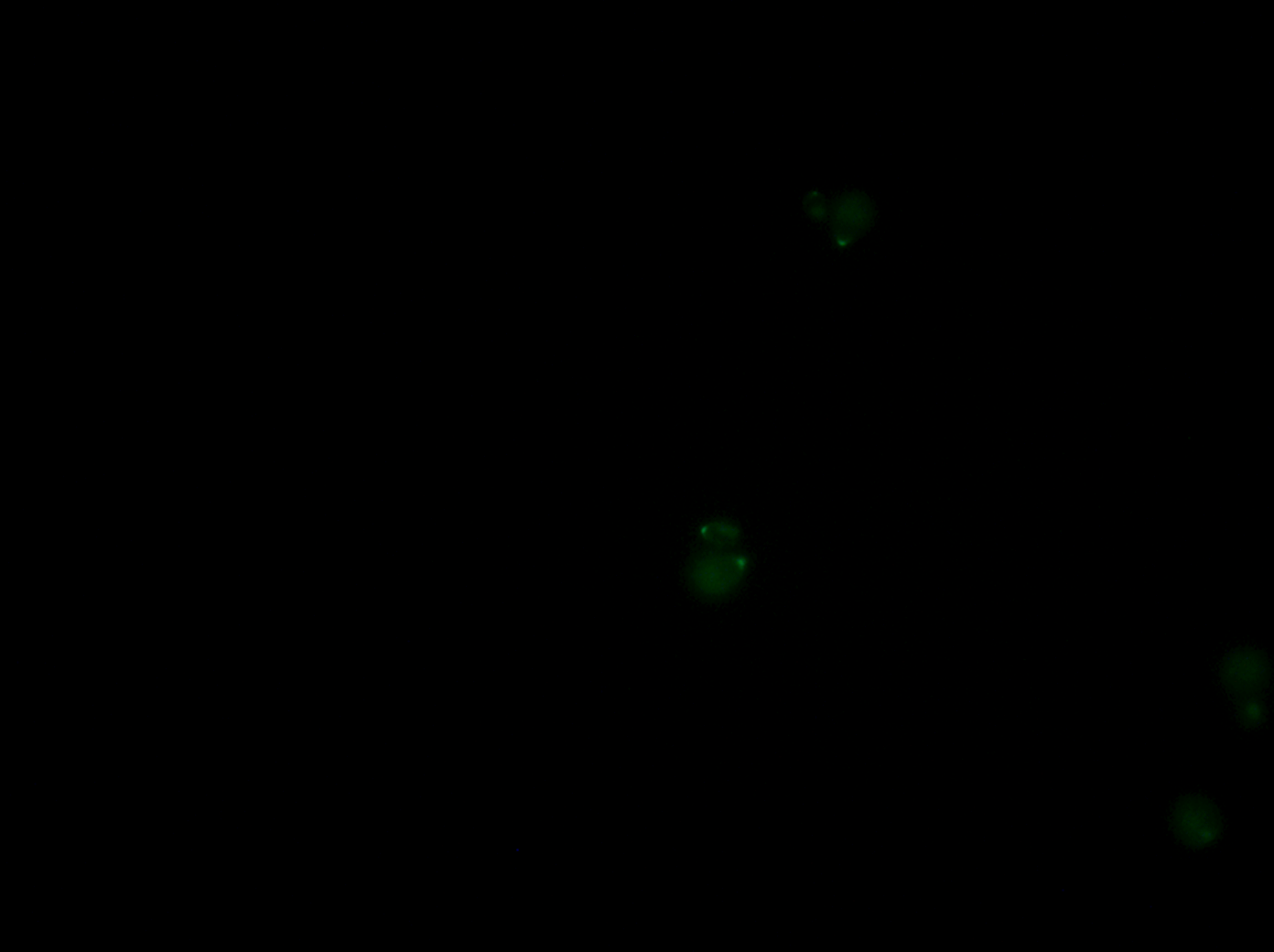

Supplement: Supplementary file 2 [file Data_Sheet_2.ZIP › Original composite images/FigureS5/A) ≡¥¢╝-pheromone.tif]

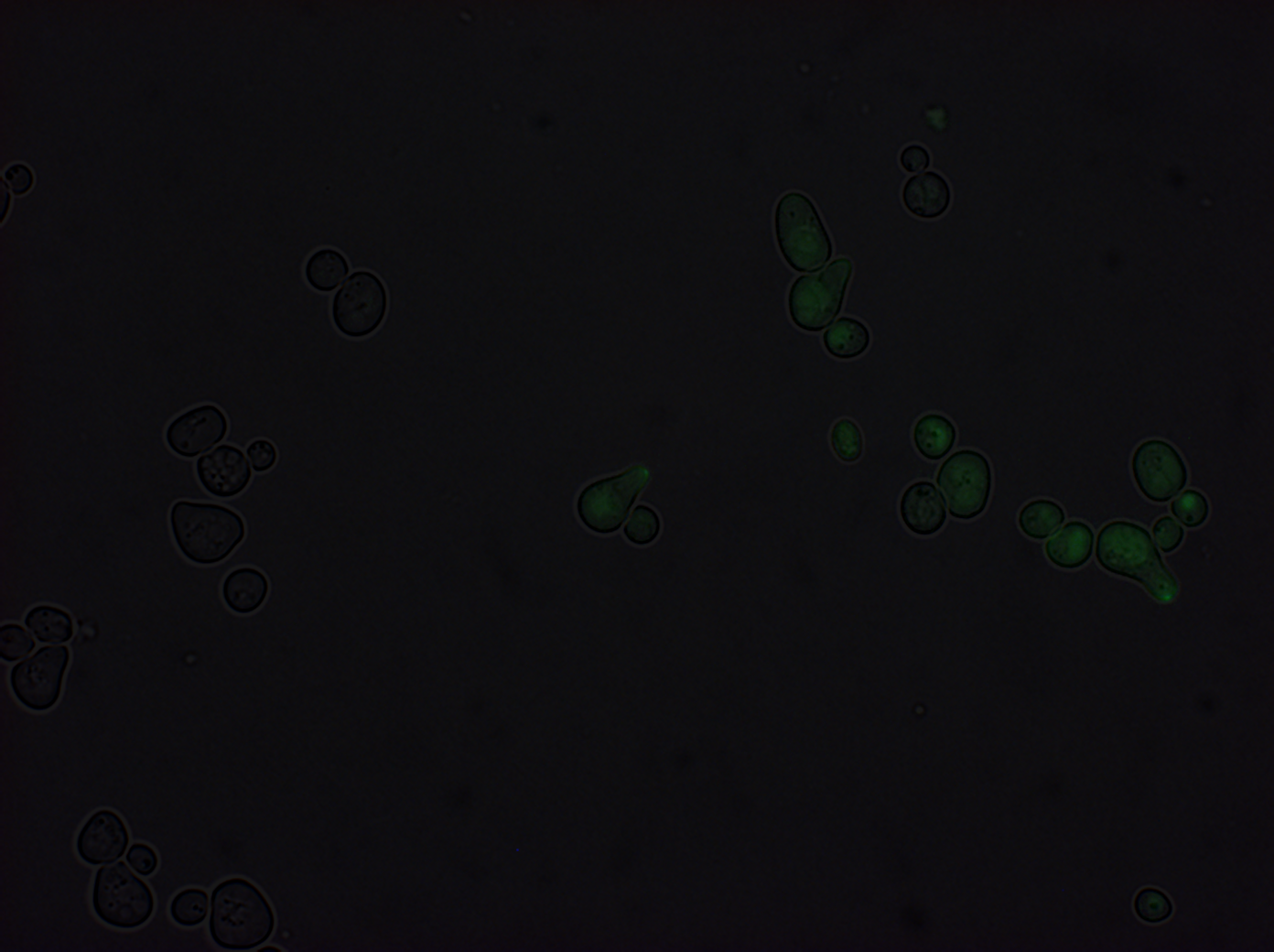

Supplement: Supplementary file 2 [file Data_Sheet_2.ZIP › Original composite images/FigureS5/B) PI-1.tif]

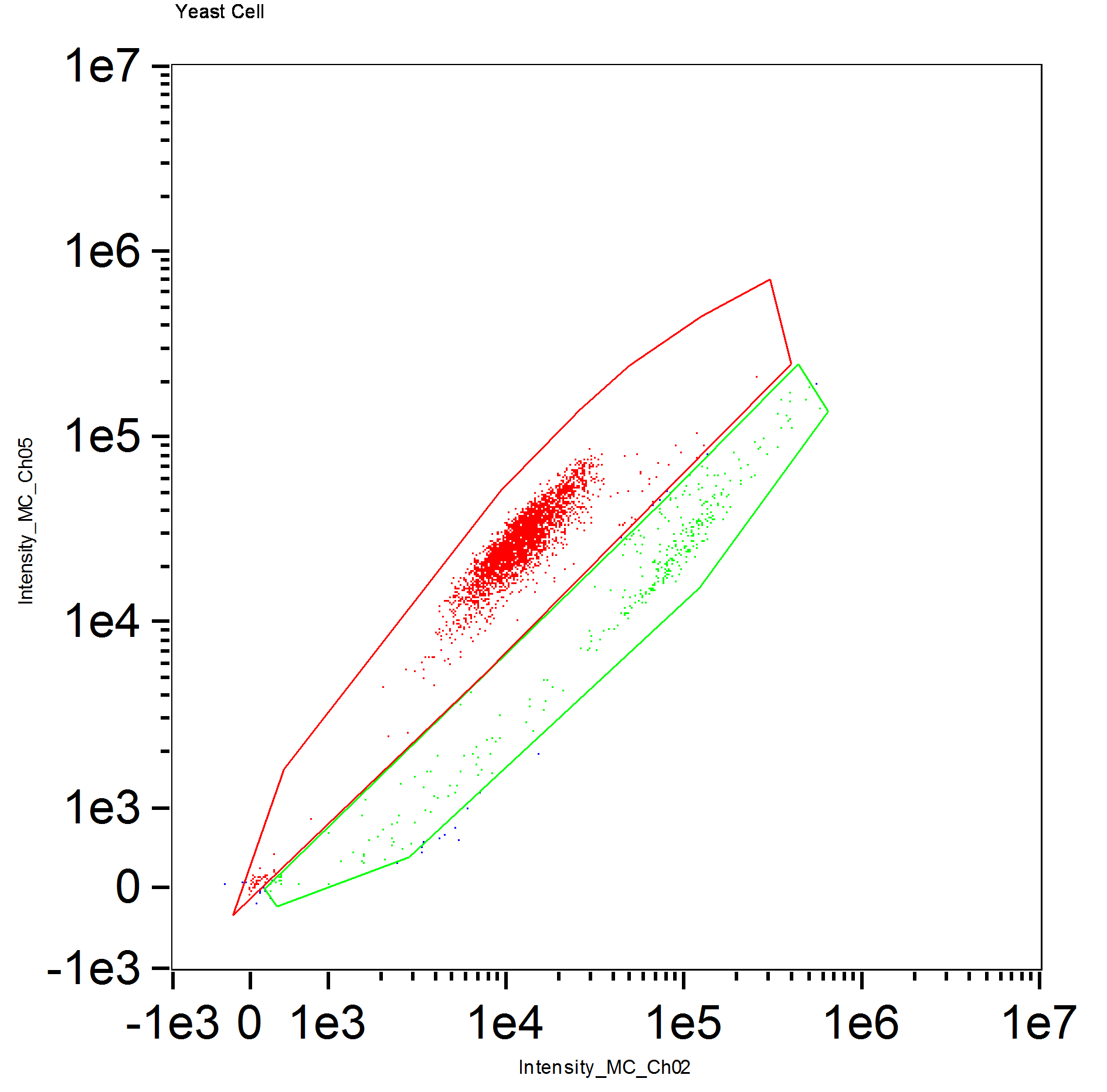

Supplement: Supplementary file 2 [file Data_Sheet_2.ZIP › Original composite images/Figure8/CDC28-as1+1-NM-PP1.png]

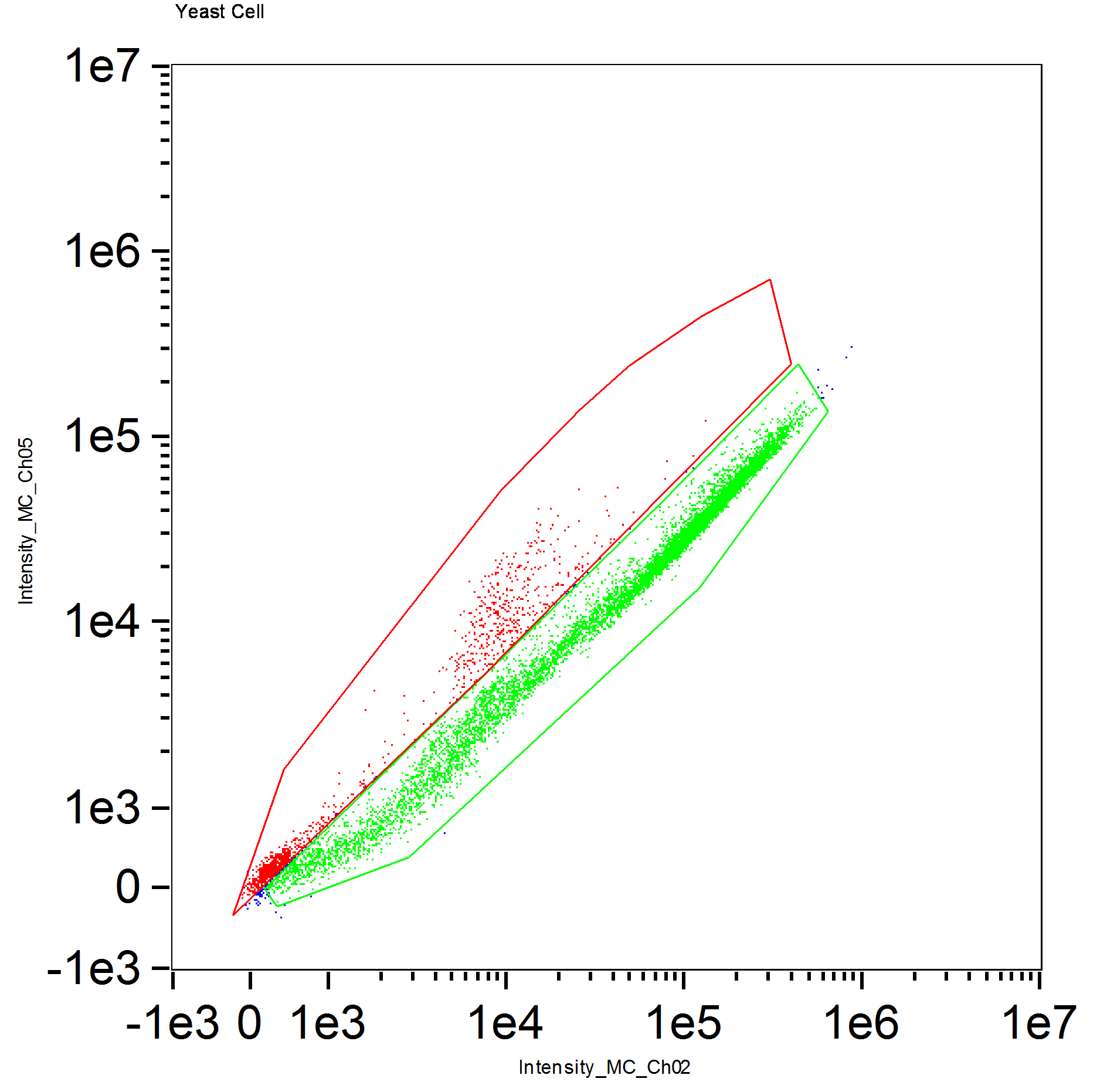

Supplement: Supplementary file 2 [file Data_Sheet_2.ZIP › Original composite images/Figure8/CDC28-as1+IP-1.png]

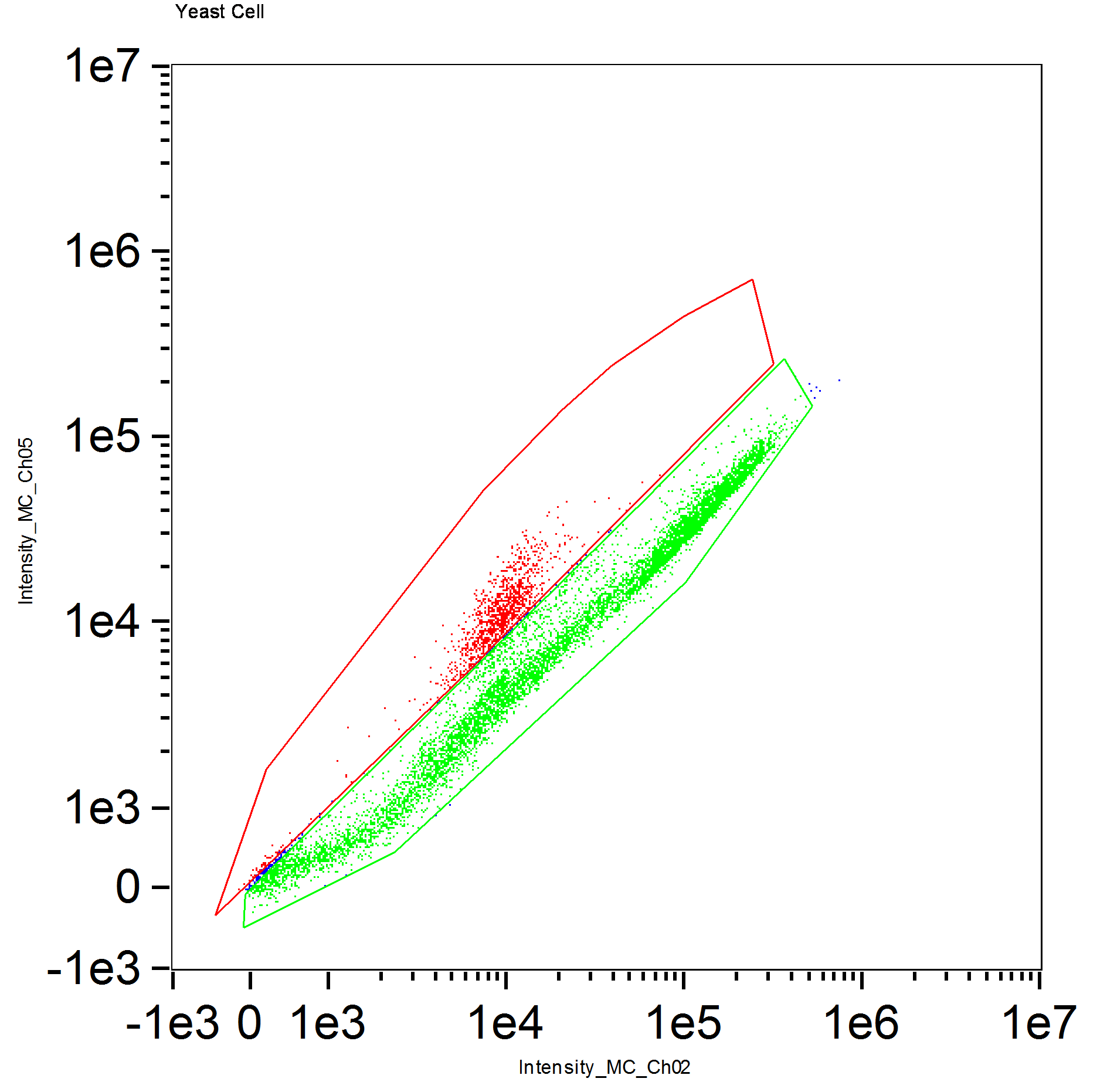

Supplement: Supplementary file 2 [file Data_Sheet_2.ZIP › Original composite images/Figure8/CDC28-as1+1-NM-PP1+IP-1-CO-NH2.png]

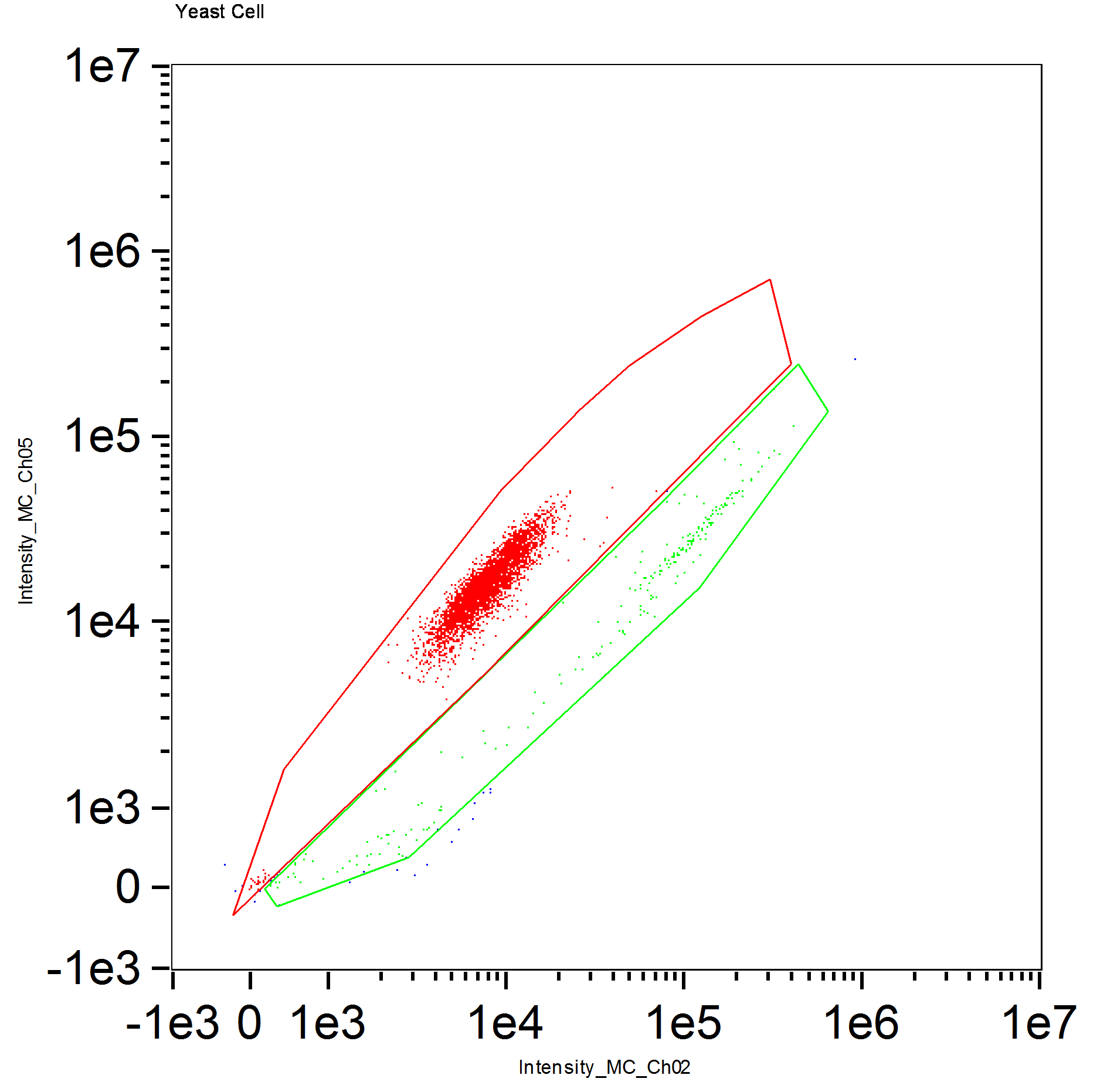

Supplement: Supplementary file 2 [file Data_Sheet_2.ZIP › Original composite images/Figure8/CDC28-as1.png]

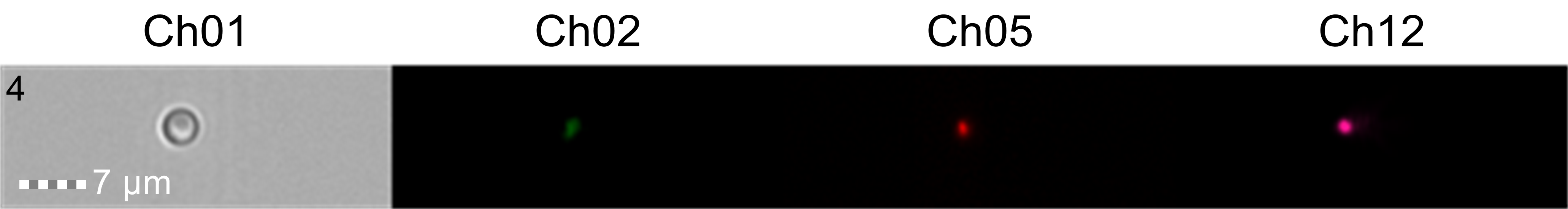

Supplement: Supplementary file 2 [file Data_Sheet_2.ZIP › Original composite images/Figure1/Live 4.png]

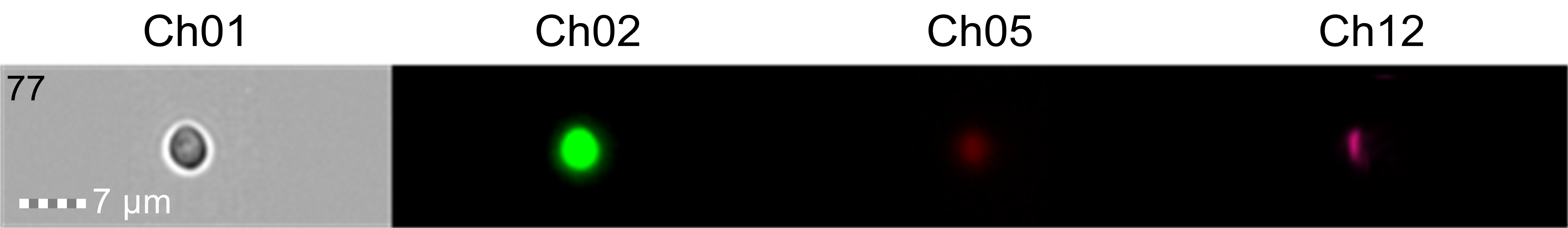

Supplement: Supplementary file 2 [file Data_Sheet_2.ZIP › Original composite images/Figure1/Dead 77.png]

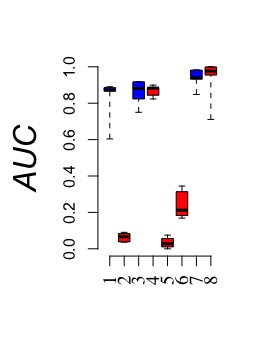

Supplement: Supplementary file 2 [file Data_Sheet_2.ZIP › Original composite images/Figure1/AUC Mat-╬▒hog1╬öKanMX.jpeg]

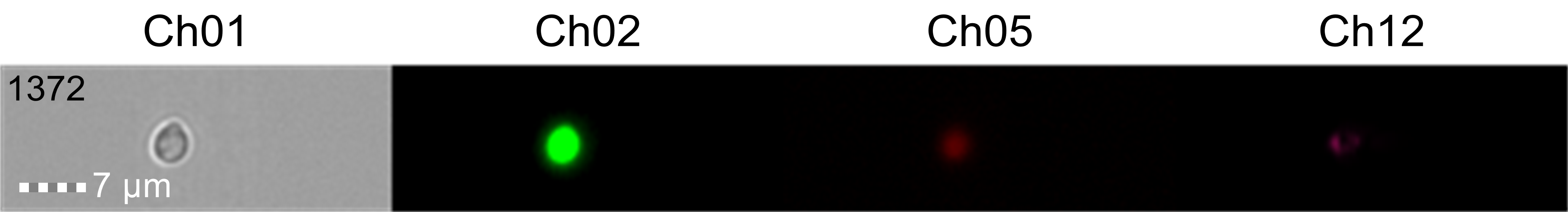

Supplement: Supplementary file 2 [file Data_Sheet_2.ZIP › Original composite images/Figure1/Dead 1327.png]

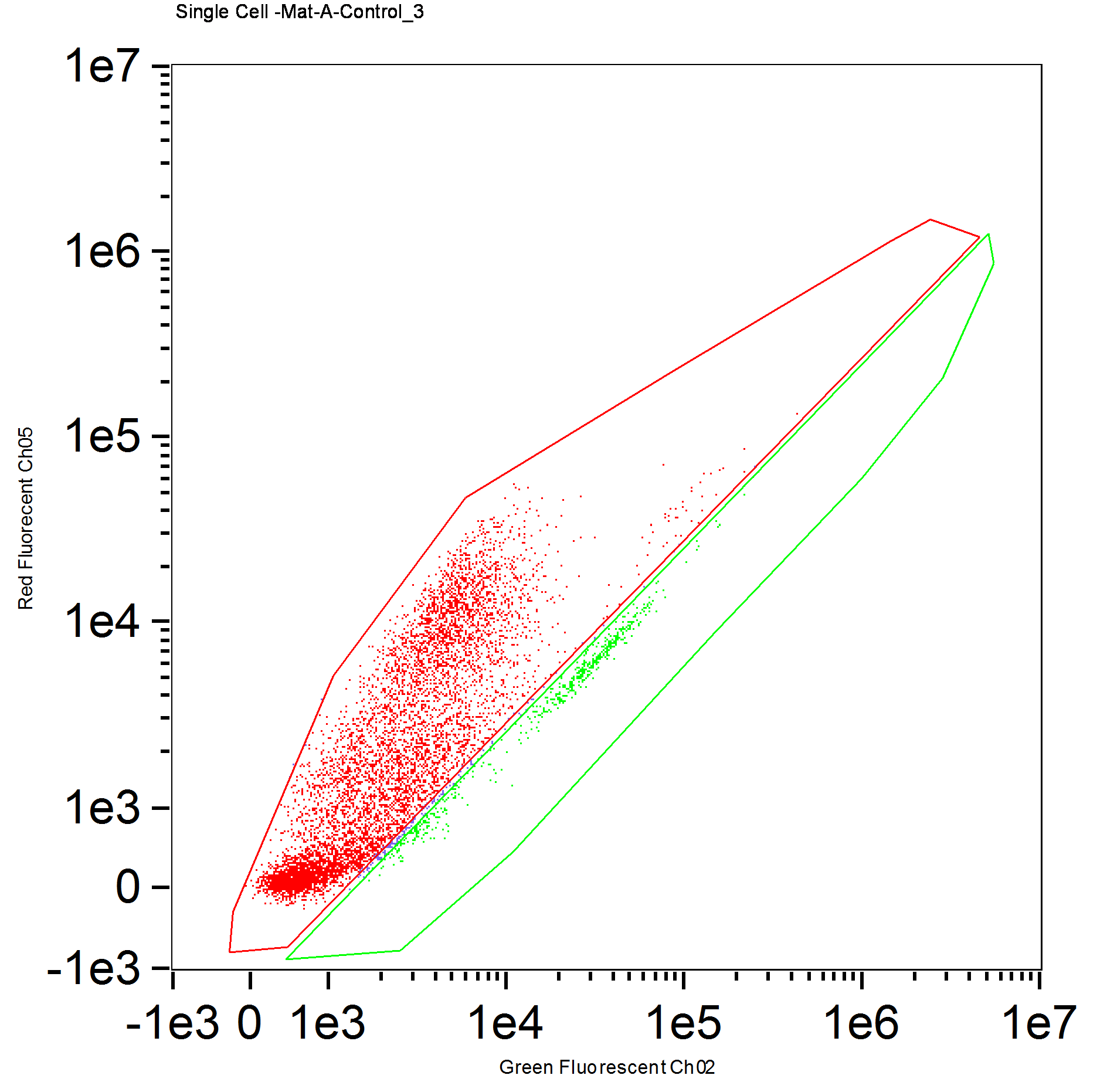

Supplement: Supplementary file 2 [file Data_Sheet_2.ZIP › Original composite images/Figure1/MatA-Live Control.png]

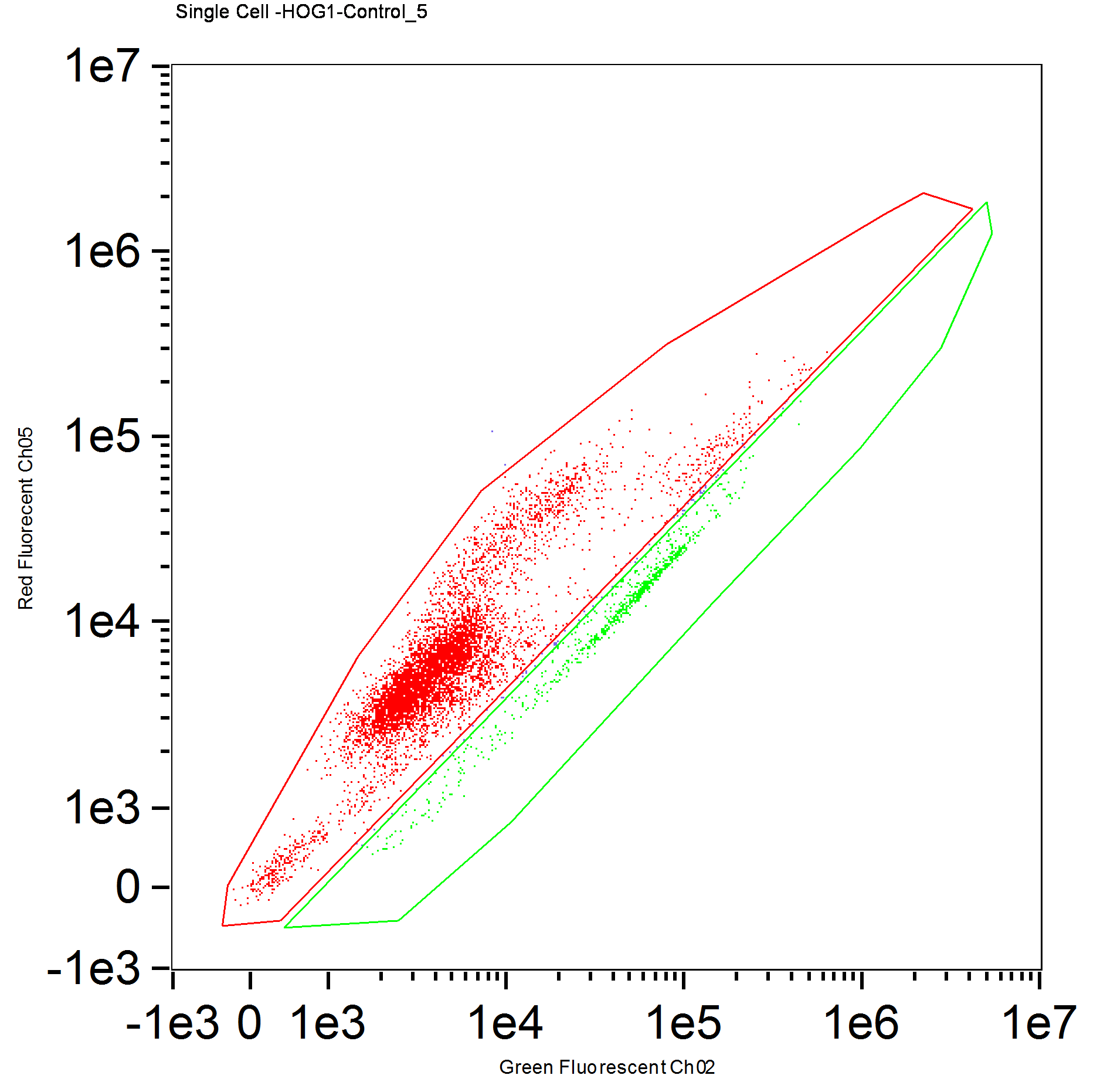

Supplement: Supplementary file 2 [file Data_Sheet_2.ZIP › Original composite images/Figure1/Mat-╬▒hog1╬öKanMX live control.png]

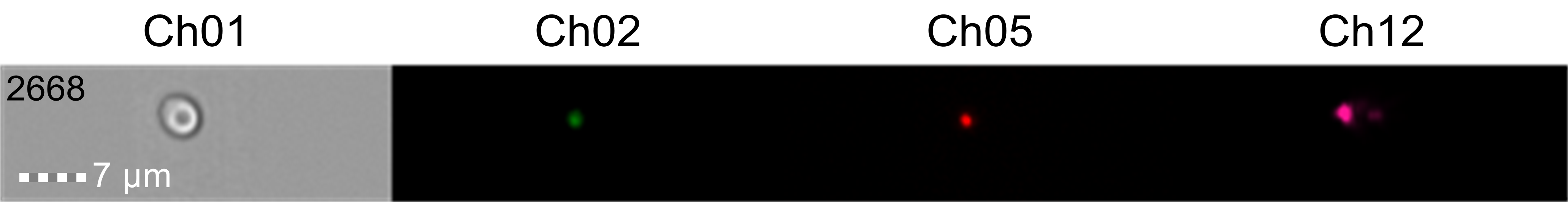

Supplement: Supplementary file 2 [file Data_Sheet_2.ZIP › Original composite images/Figure1/Live 2668.png]

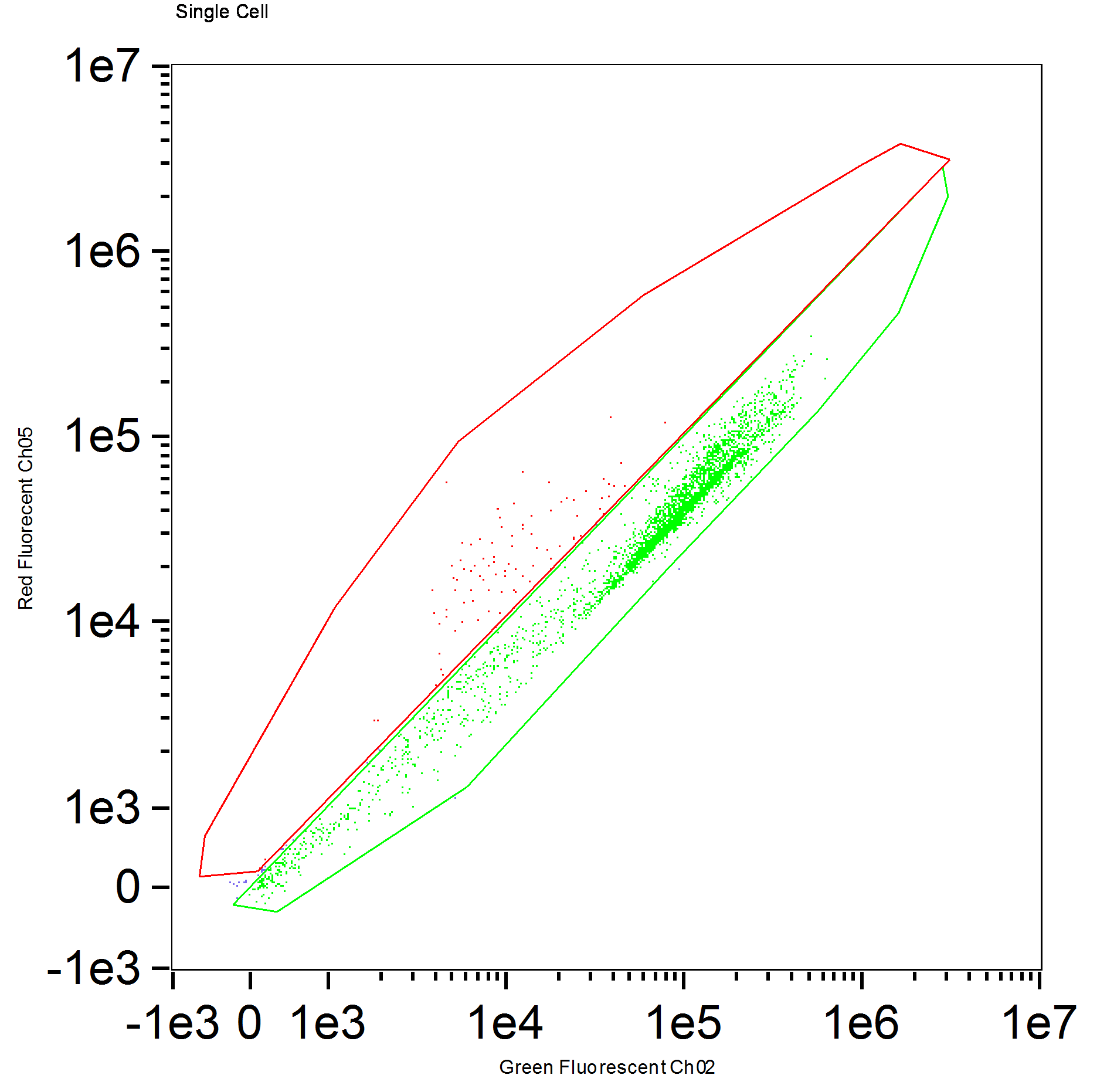

Supplement: Supplementary file 2 [file Data_Sheet_2.ZIP › Original composite images/Figure1/Mat-╬▒hog1╬öKanMX-Peptido Iztli-1.png]

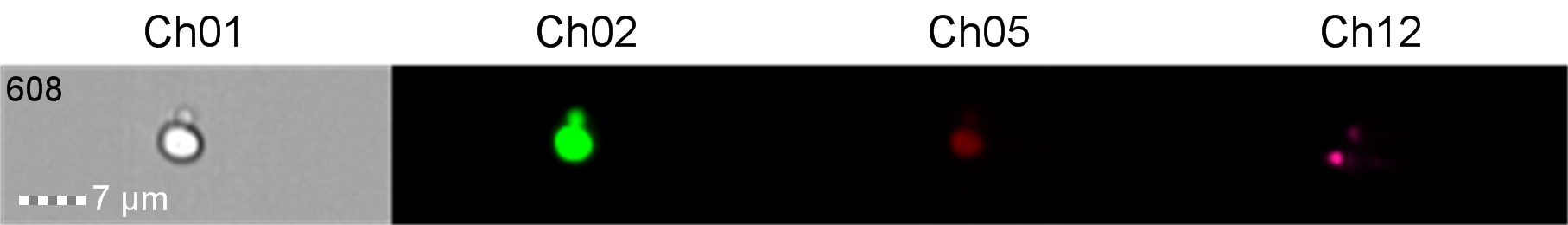

Supplement: Supplementary file 2 [file Data_Sheet_2.ZIP › Original composite images/Figure1/Dead 608.png]

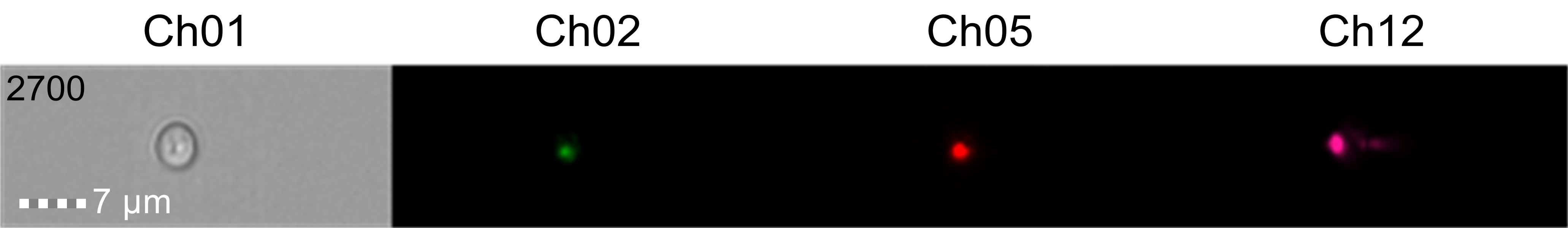

Supplement: Supplementary file 2 [file Data_Sheet_2.ZIP › Original composite images/Figure1/Live 2700.png]

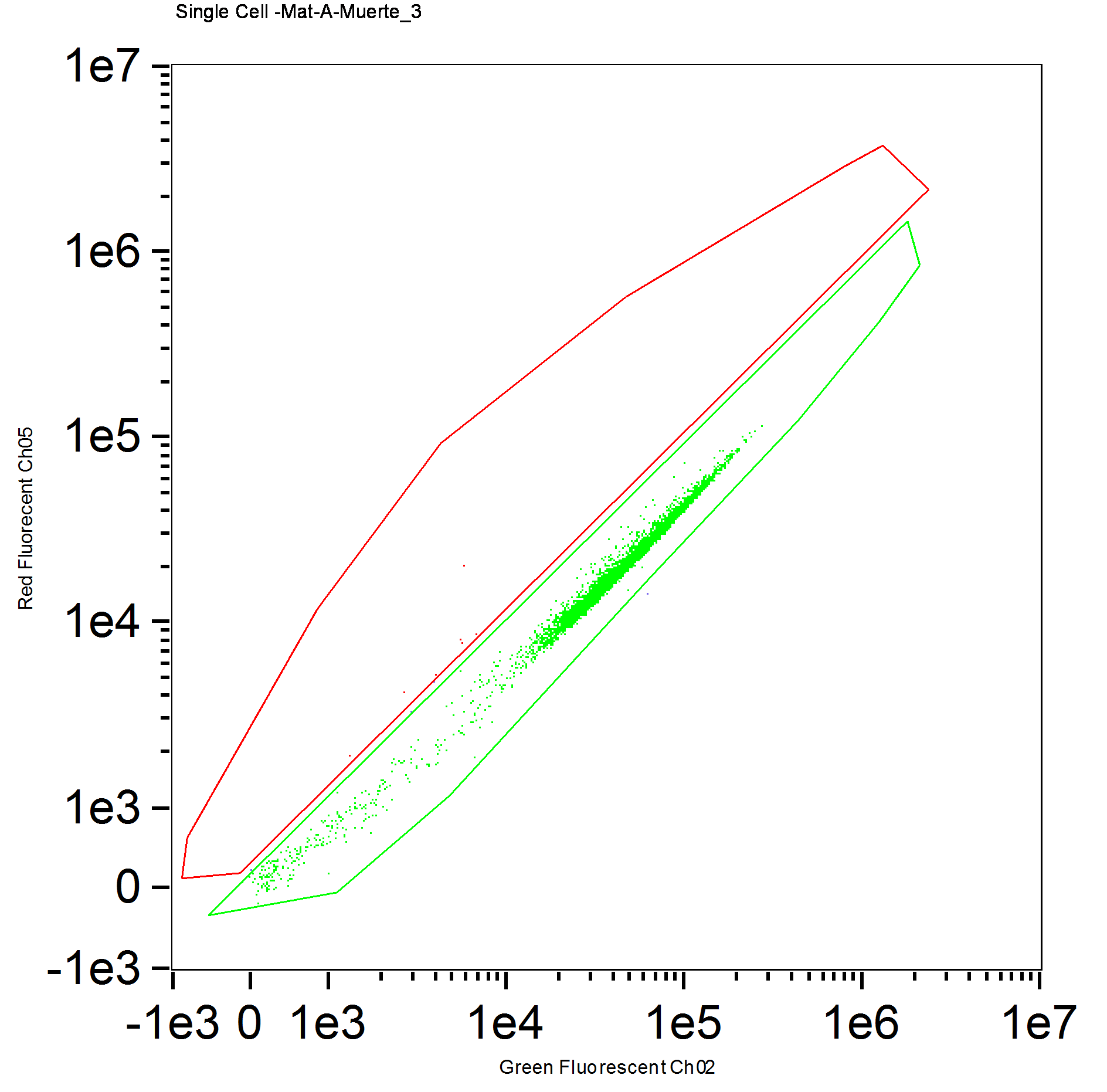

Supplement: Supplementary file 2 [file Data_Sheet_2.ZIP › Original composite images/Figure1/Mat-╬▒hog1╬öKanMX Dead control.png]

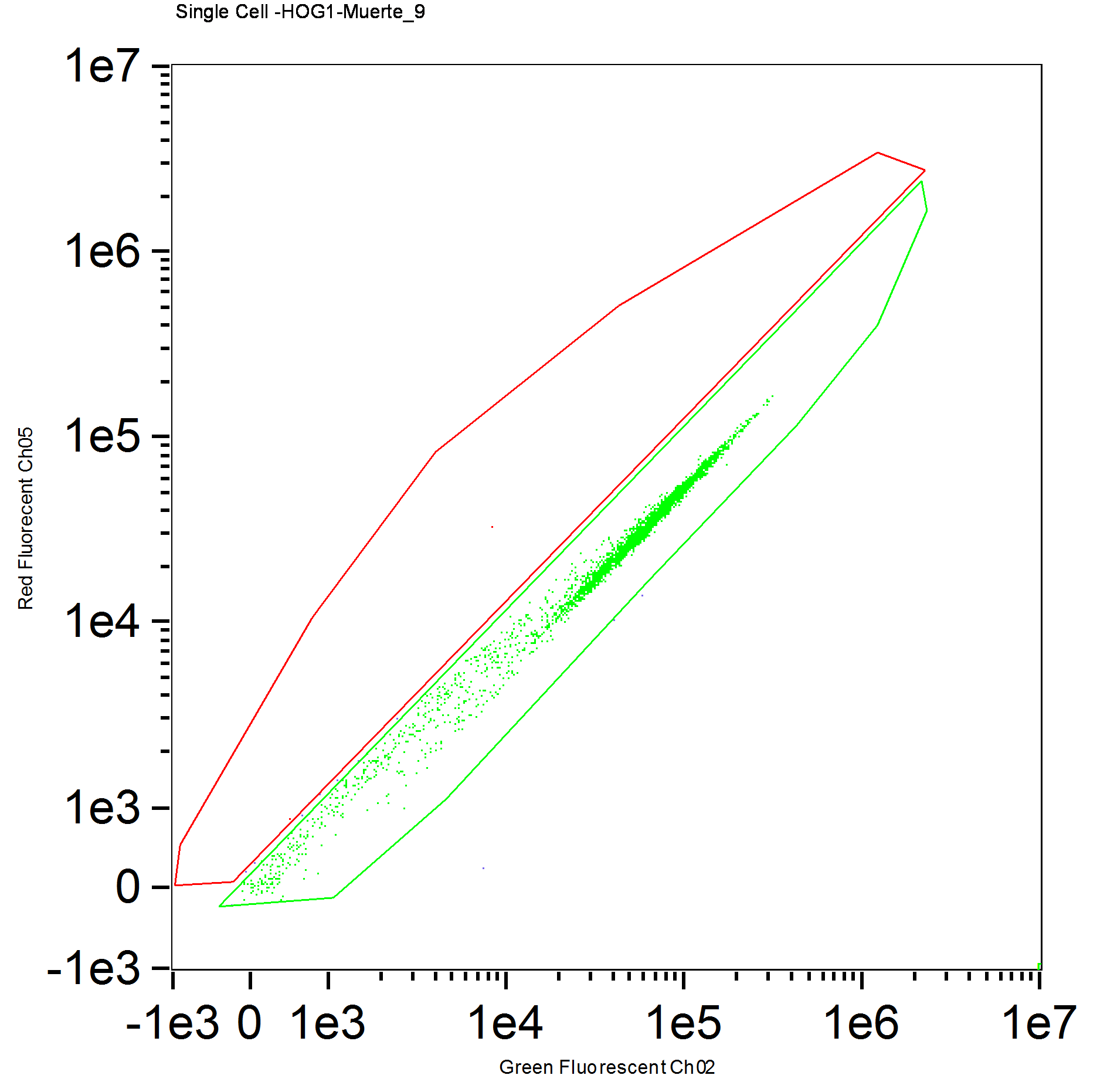

Supplement: Supplementary file 2 [file Data_Sheet_2.ZIP › Original composite images/Figure1/MatA-Dead control.png]

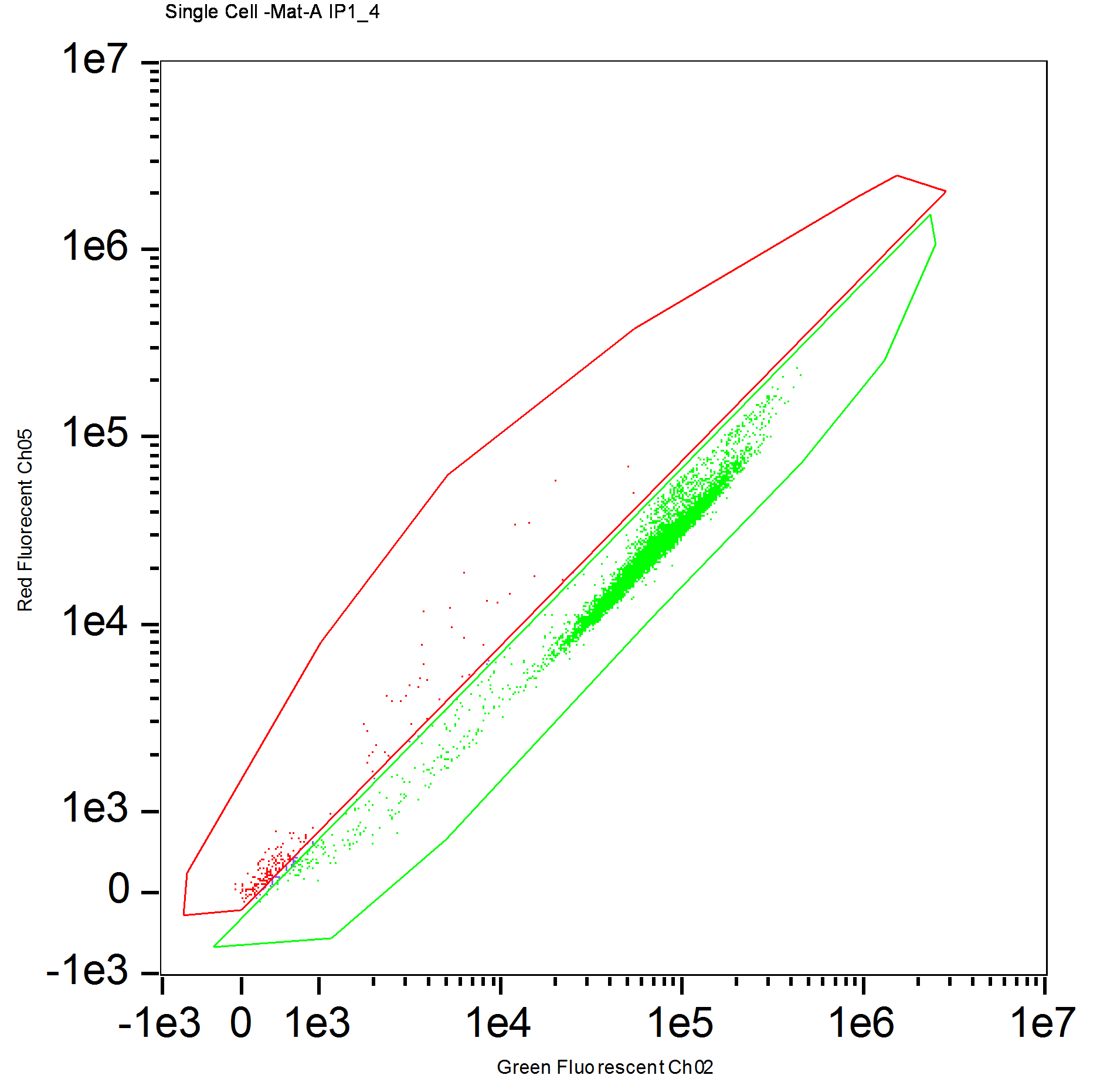

Supplement: Supplementary file 2 [file Data_Sheet_2.ZIP › Original composite images/Figure1/MatA-IP.png]

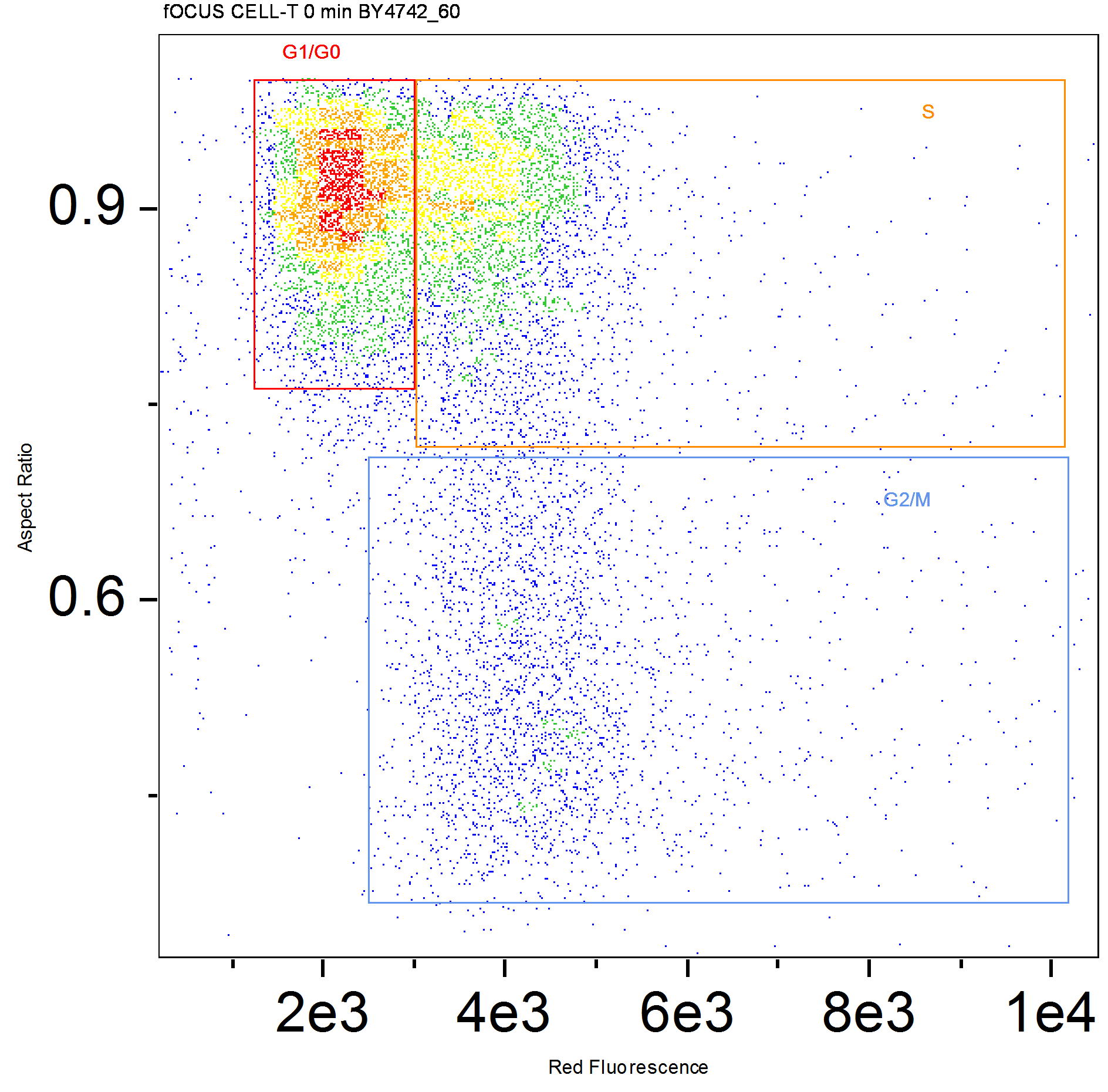

Supplement: Supplementary file 2 [file Data_Sheet_2.ZIP › Original composite images/Figure6/Cell Cycle Control.png]

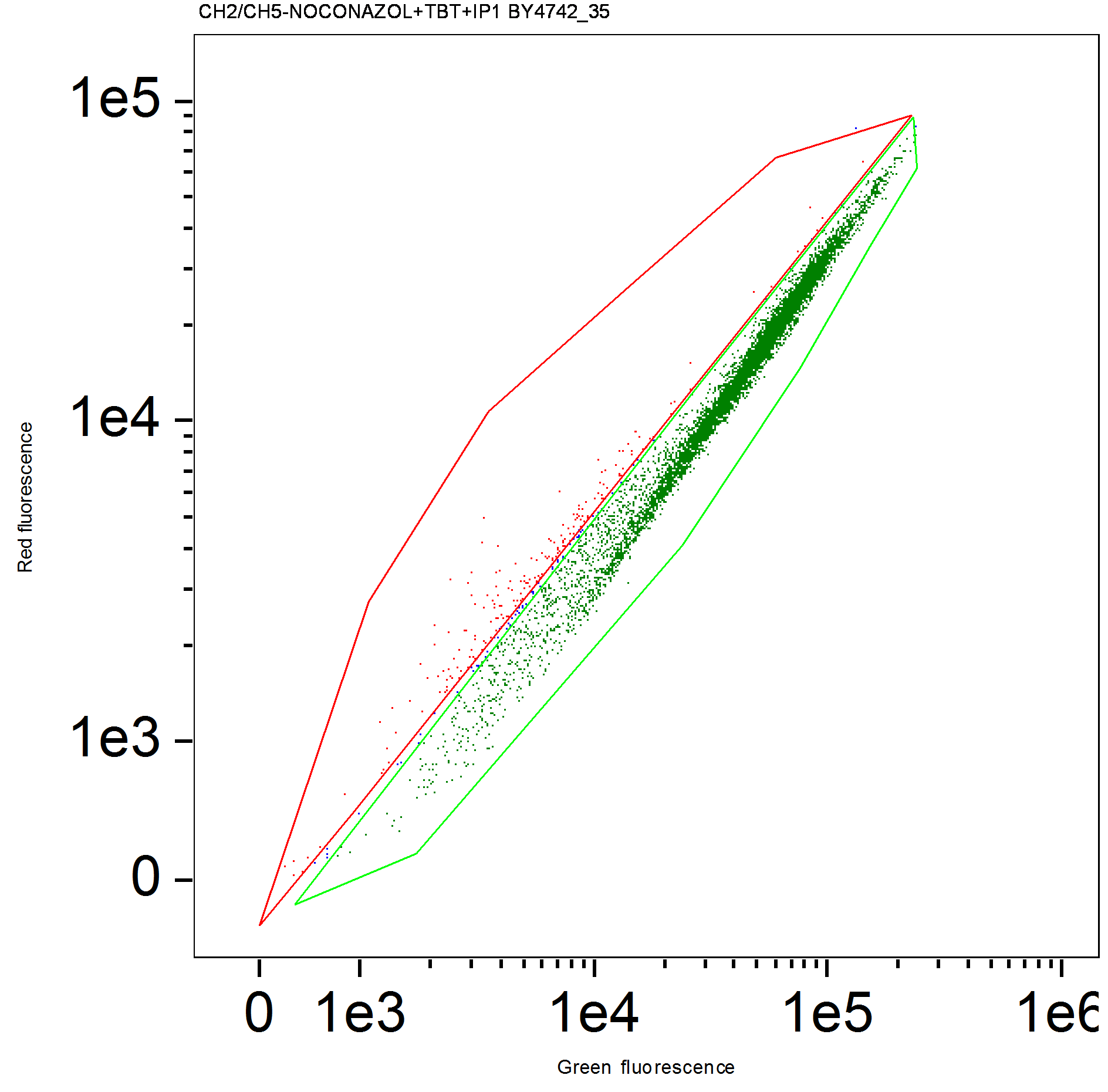

Supplement: Supplementary file 2 [file Data_Sheet_2.ZIP › Original composite images/Figure6/Viability Nocodazole+TBT+IP-1.png]

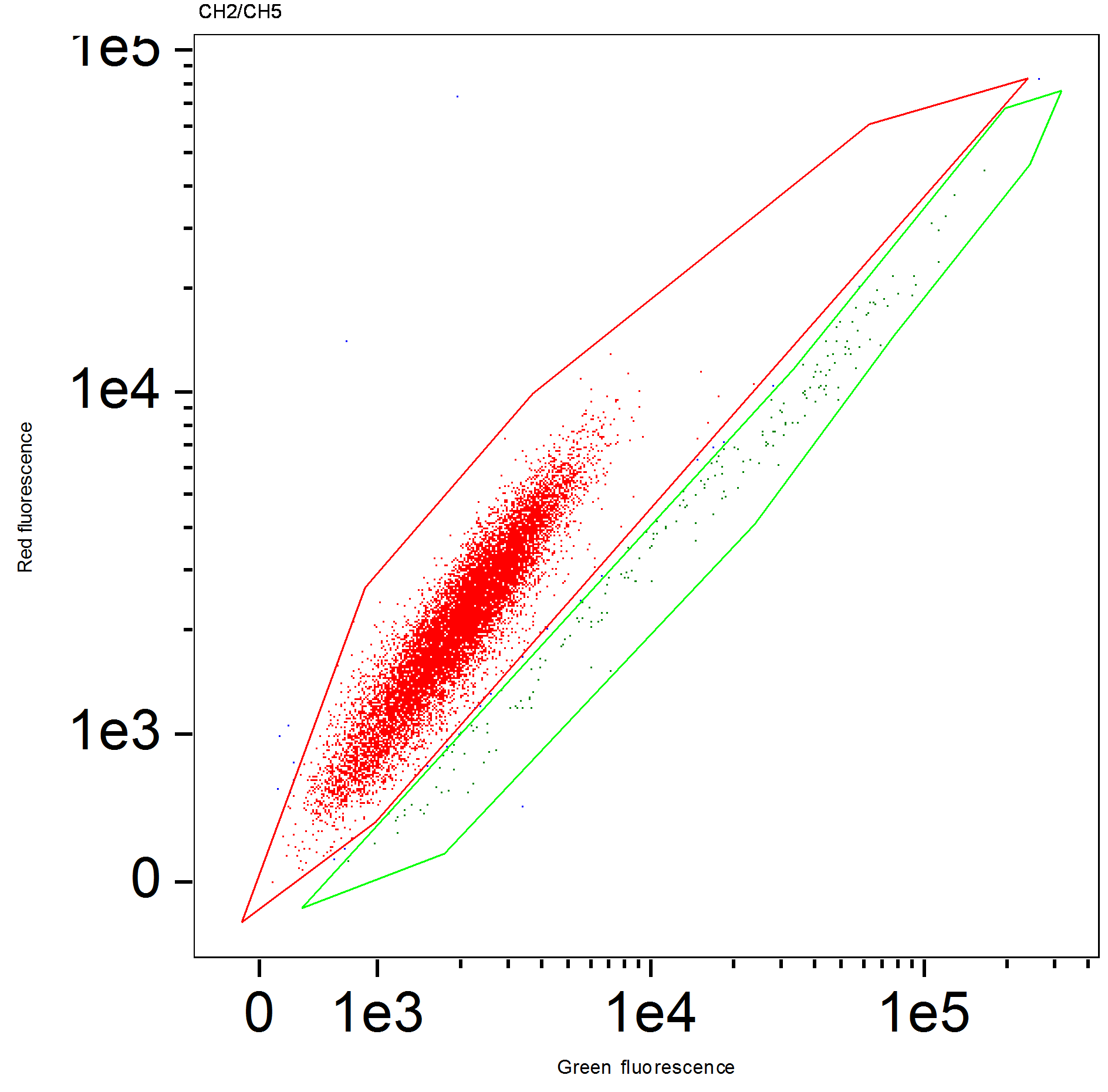

Supplement: Supplementary file 2 [file Data_Sheet_2.ZIP › Original composite images/Figure6/Viability Control.png]

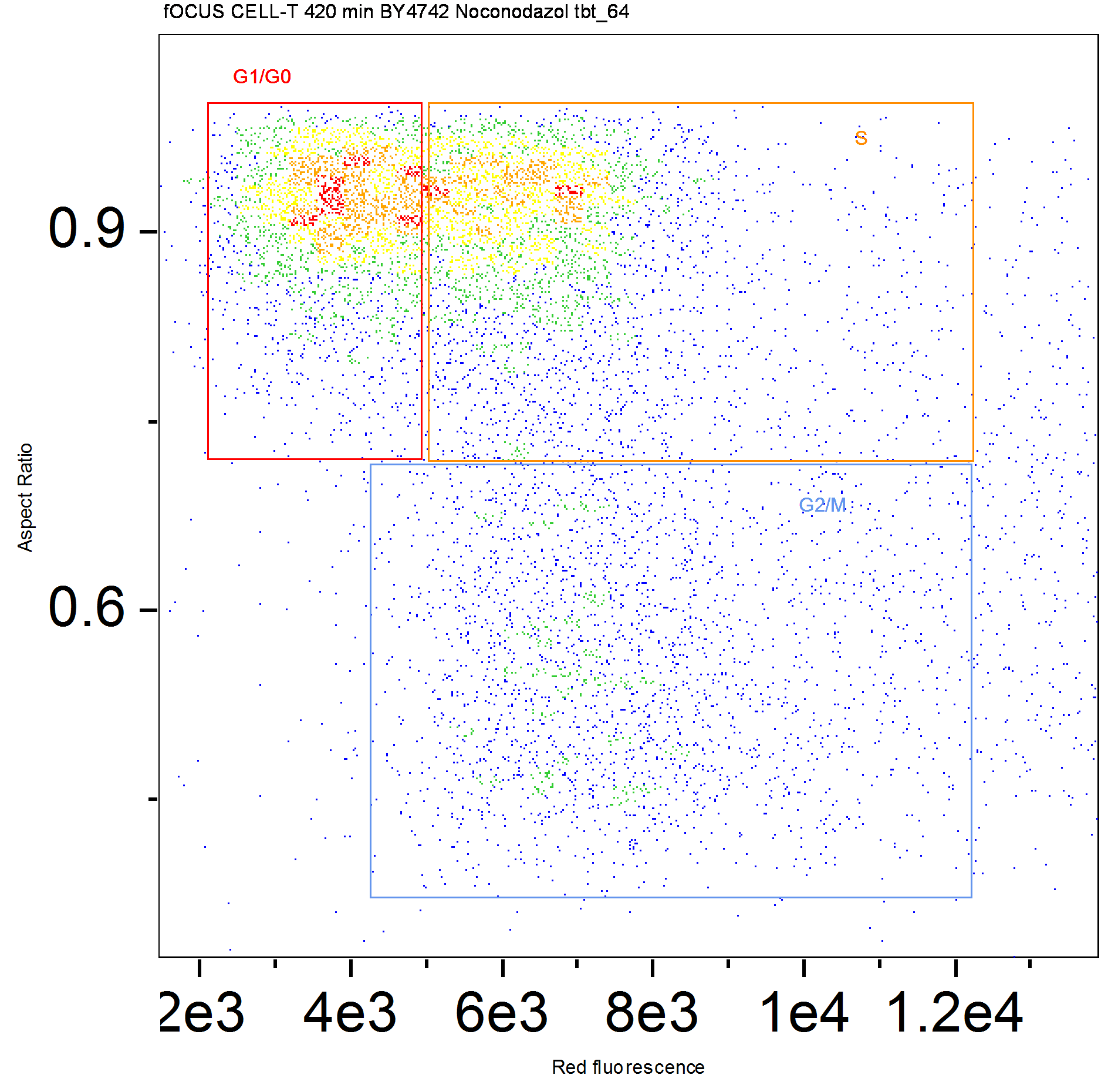

Supplement: Supplementary file 2 [file Data_Sheet_2.ZIP › Original composite images/Figure6/Cell Cycle Nocodazole+TBT+Without PI-1.png]

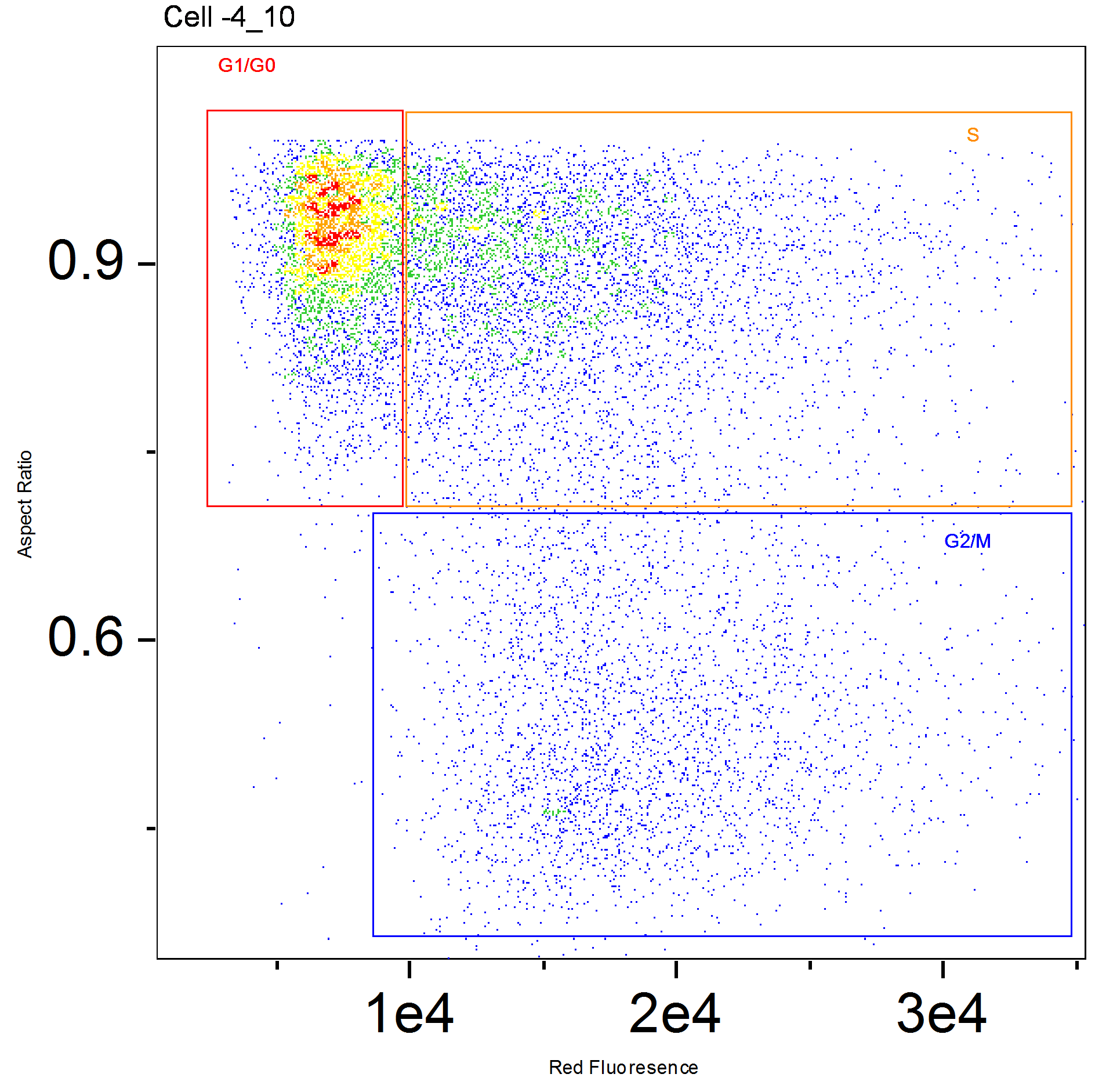

Supplement: Supplementary file 2 [file Data_Sheet_2.ZIP › Original composite images/Figure6/Cell Cycle Nocodazole+TBT.png]

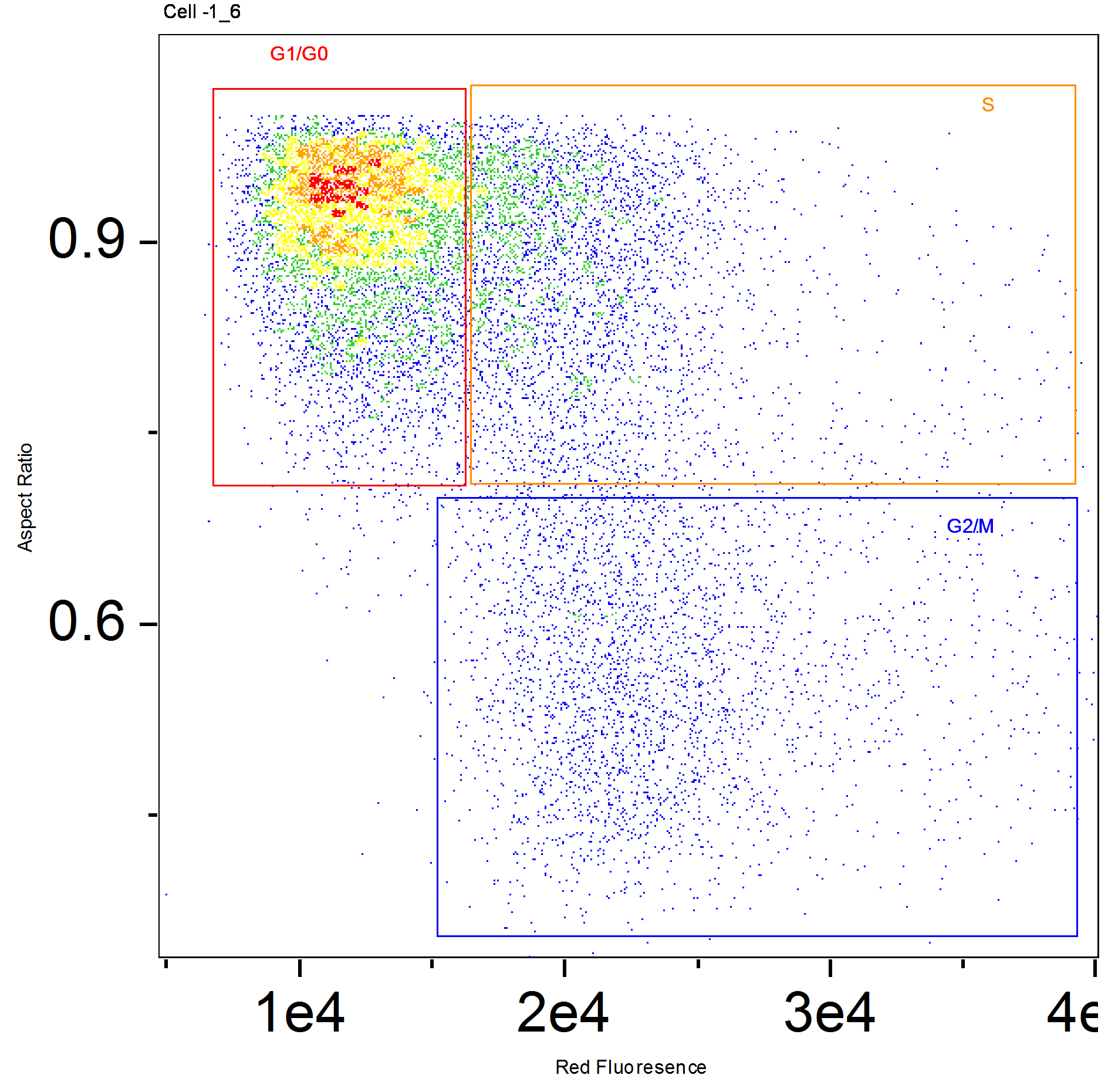

Supplement: Supplementary file 2 [file Data_Sheet_2.ZIP › Original composite images/Figure6/Cell Cycle Nocodazole+TBT+IP1.png]

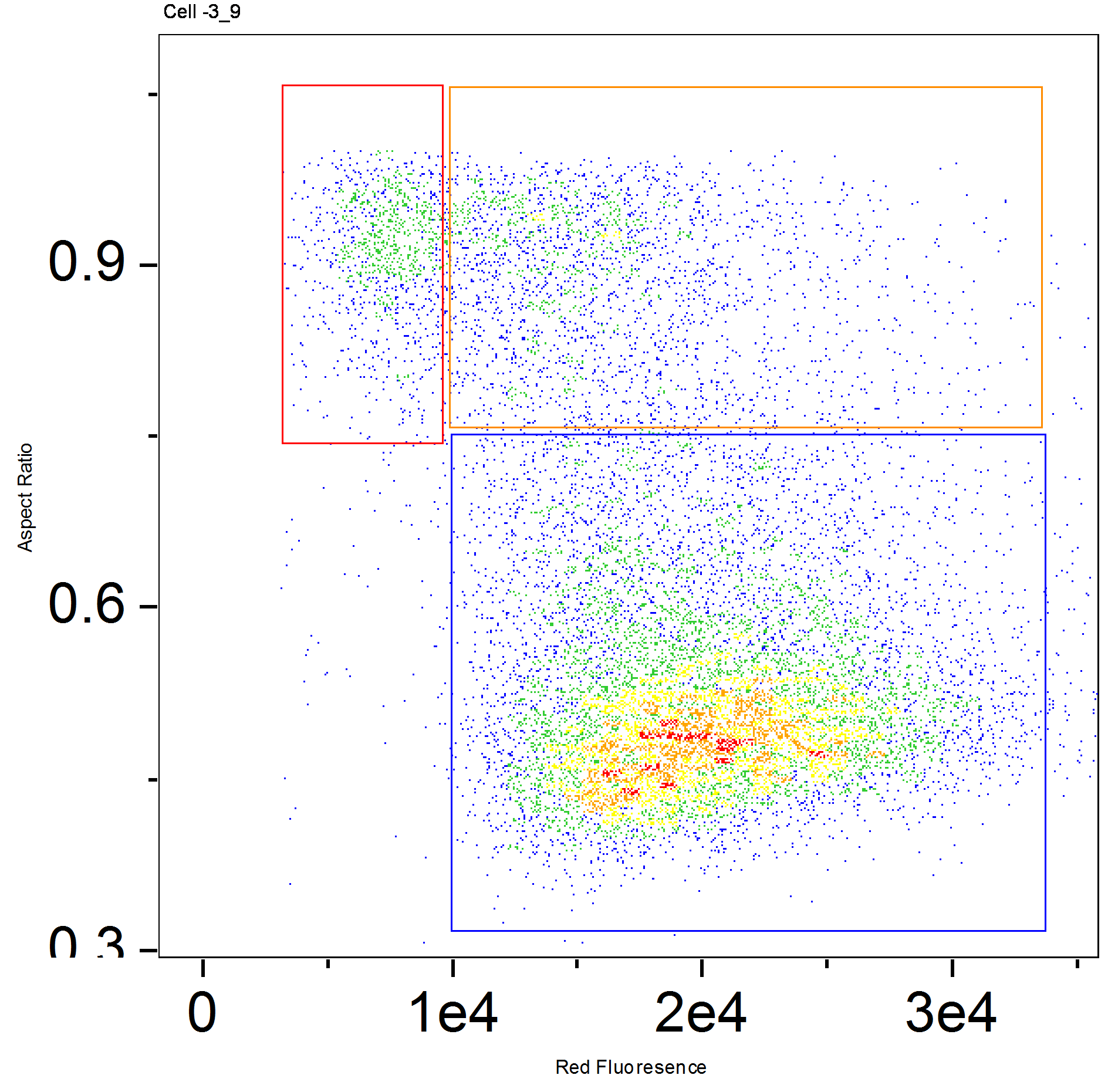

Supplement: Supplementary file 2 [file Data_Sheet_2.ZIP › Original composite images/Figure6/Cell Cycle Nocodazole.png]

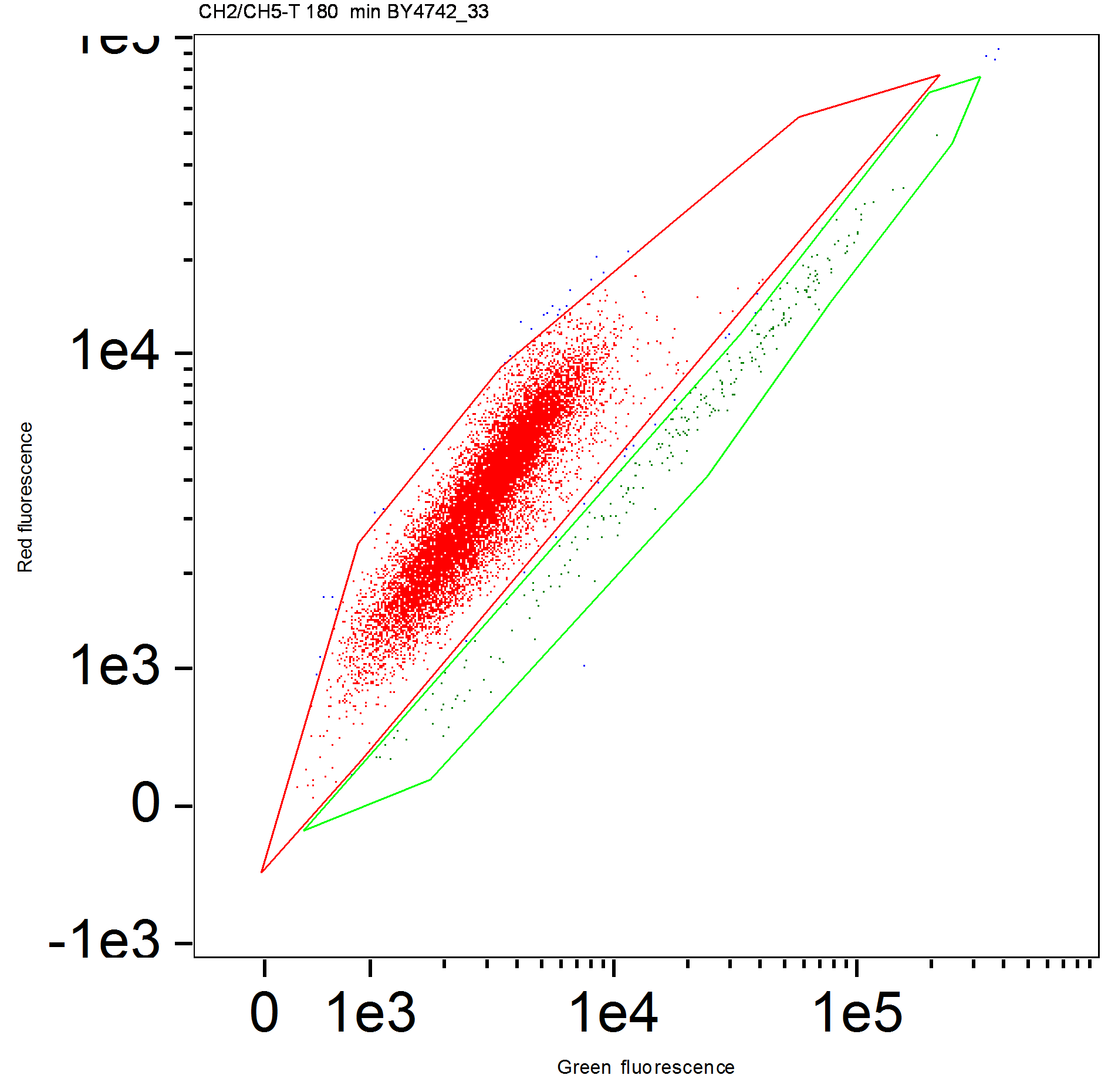

Supplement: Supplementary file 2 [file Data_Sheet_2.ZIP › Original composite images/Figure6/Viavility Nocodazole+TBT+Without IP-1.png]

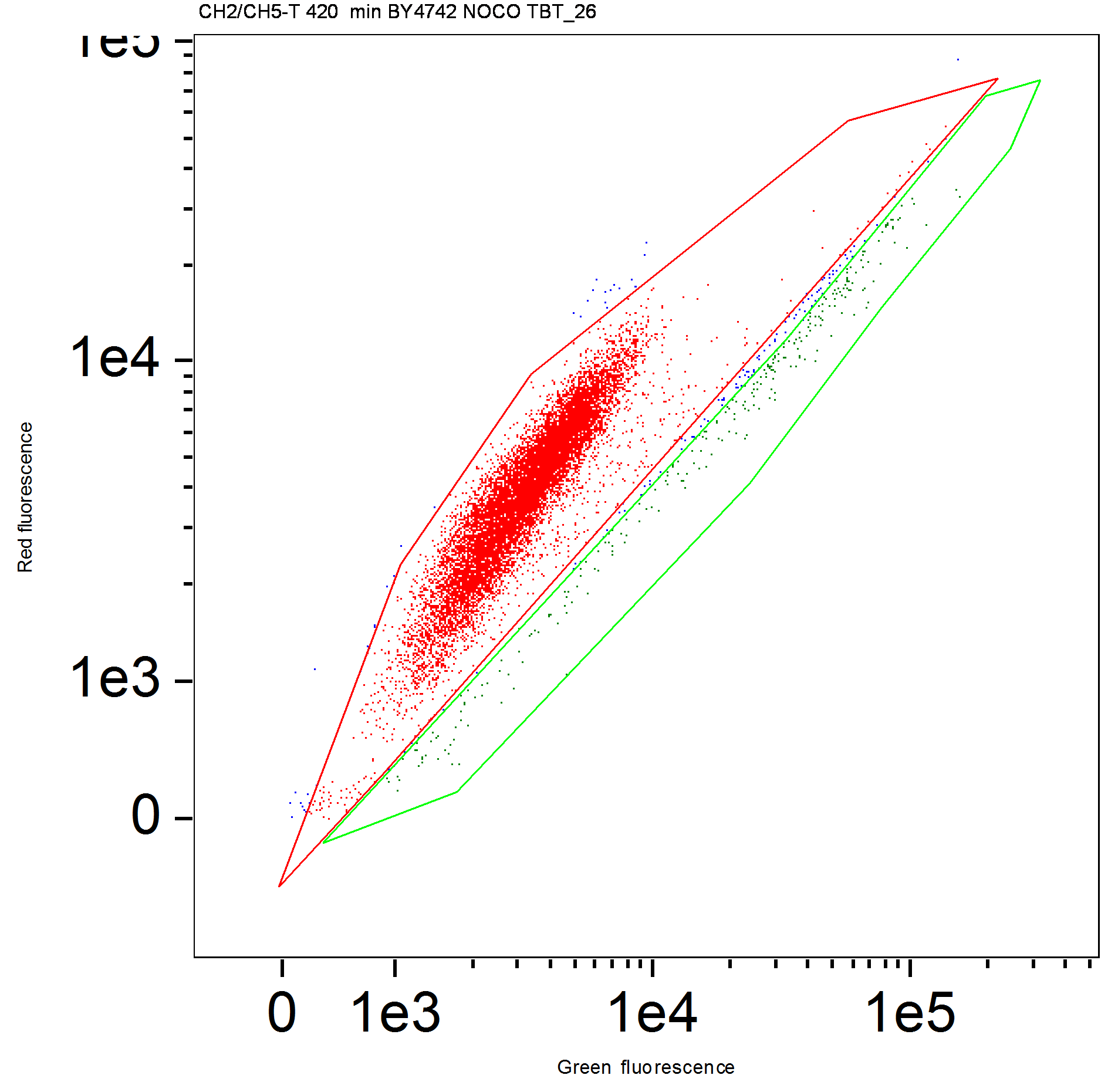

Supplement: Supplementary file 2 [file Data_Sheet_2.ZIP › Original composite images/Figure6/Viability Nocodazole+TBT.png]

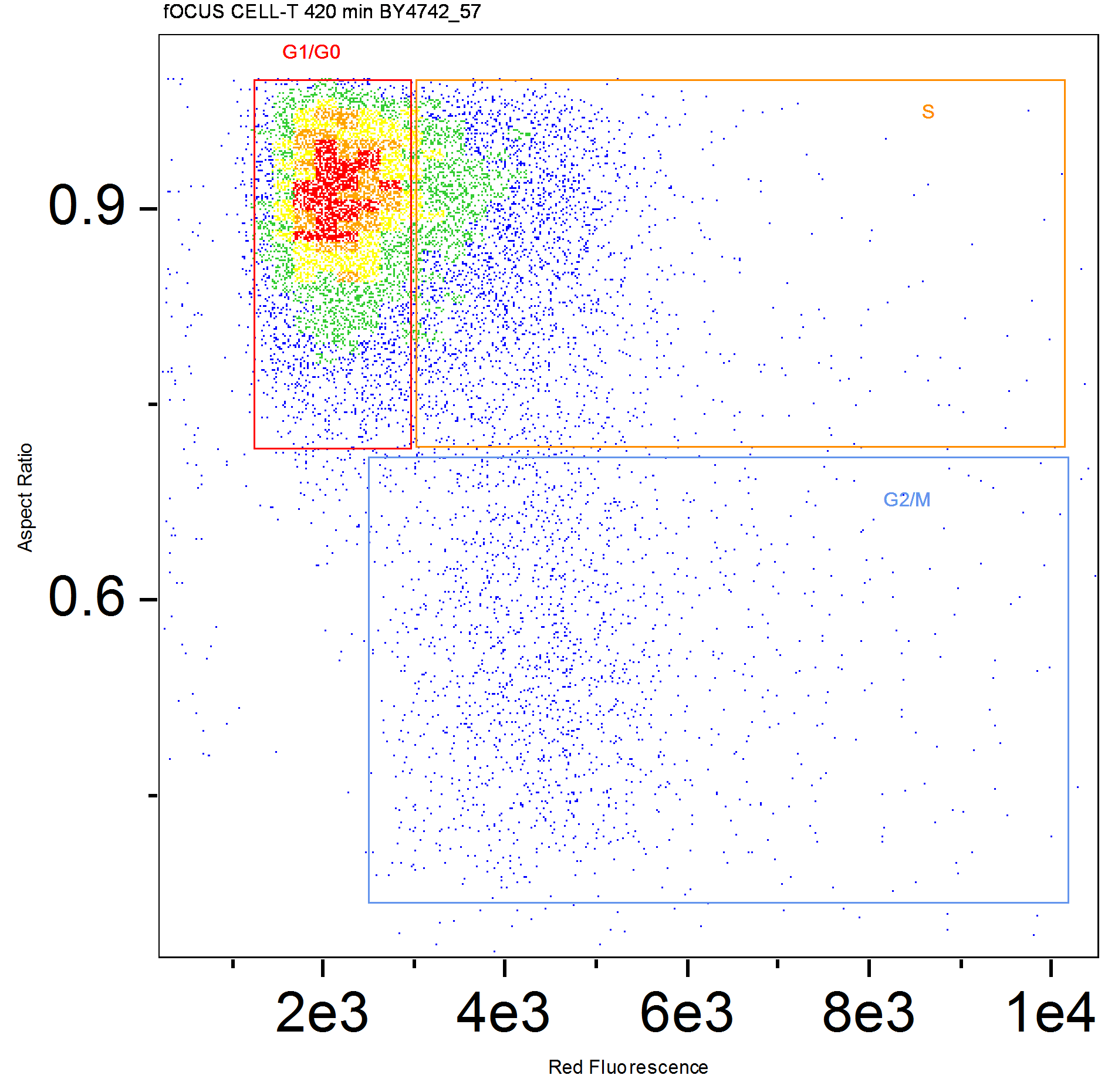

Supplement: Supplementary file 2 [file Data_Sheet_2.ZIP › Original composite images/Figure6/Cell Cycle Nocodazole+TBT+IP-1-CO-NH2.png]

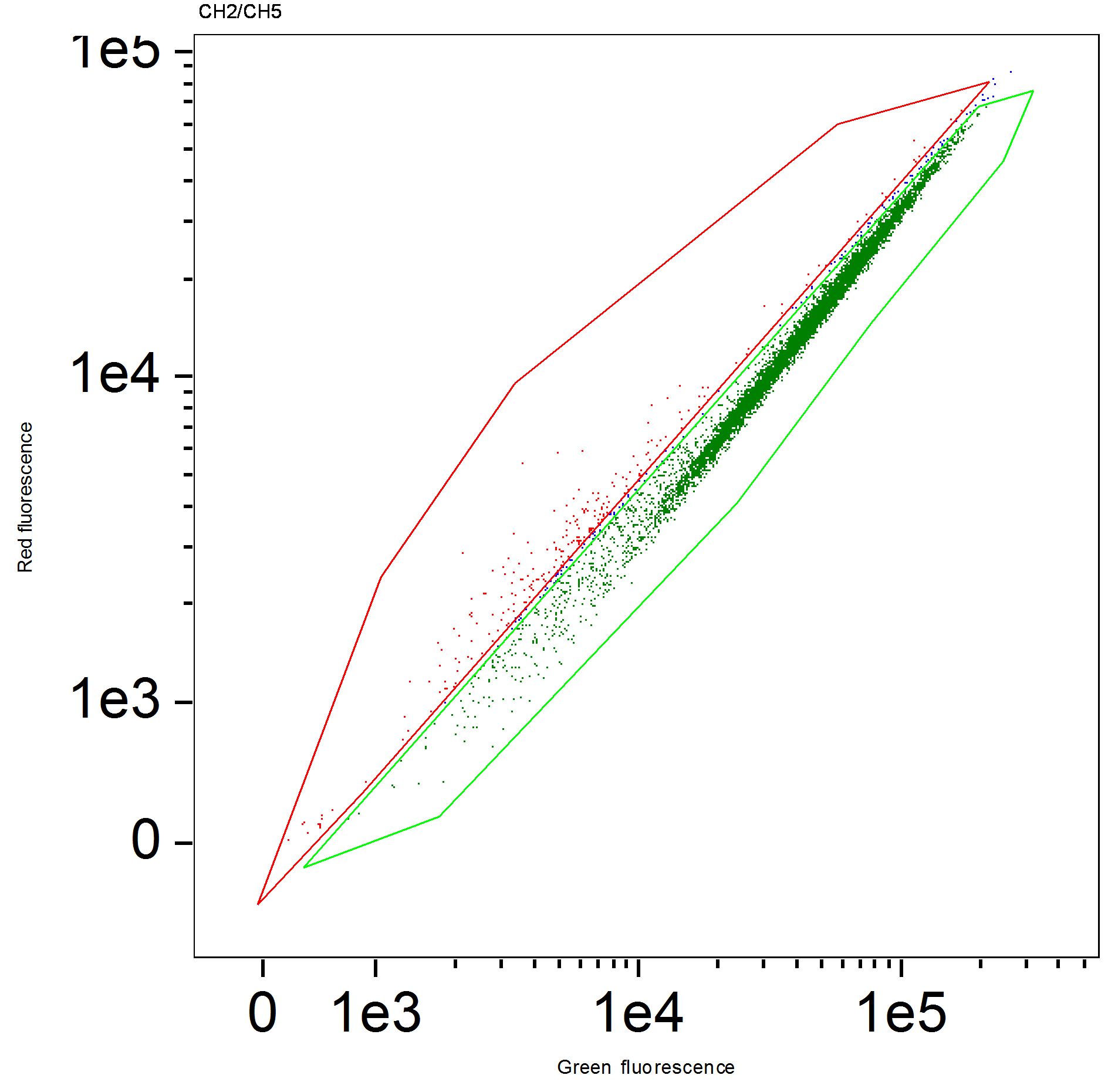

Supplement: Supplementary file 2 [file Data_Sheet_2.ZIP › Original composite images/Figure6/Viavility Nocodazole+TBT+IP-1-CO-NH2.png]

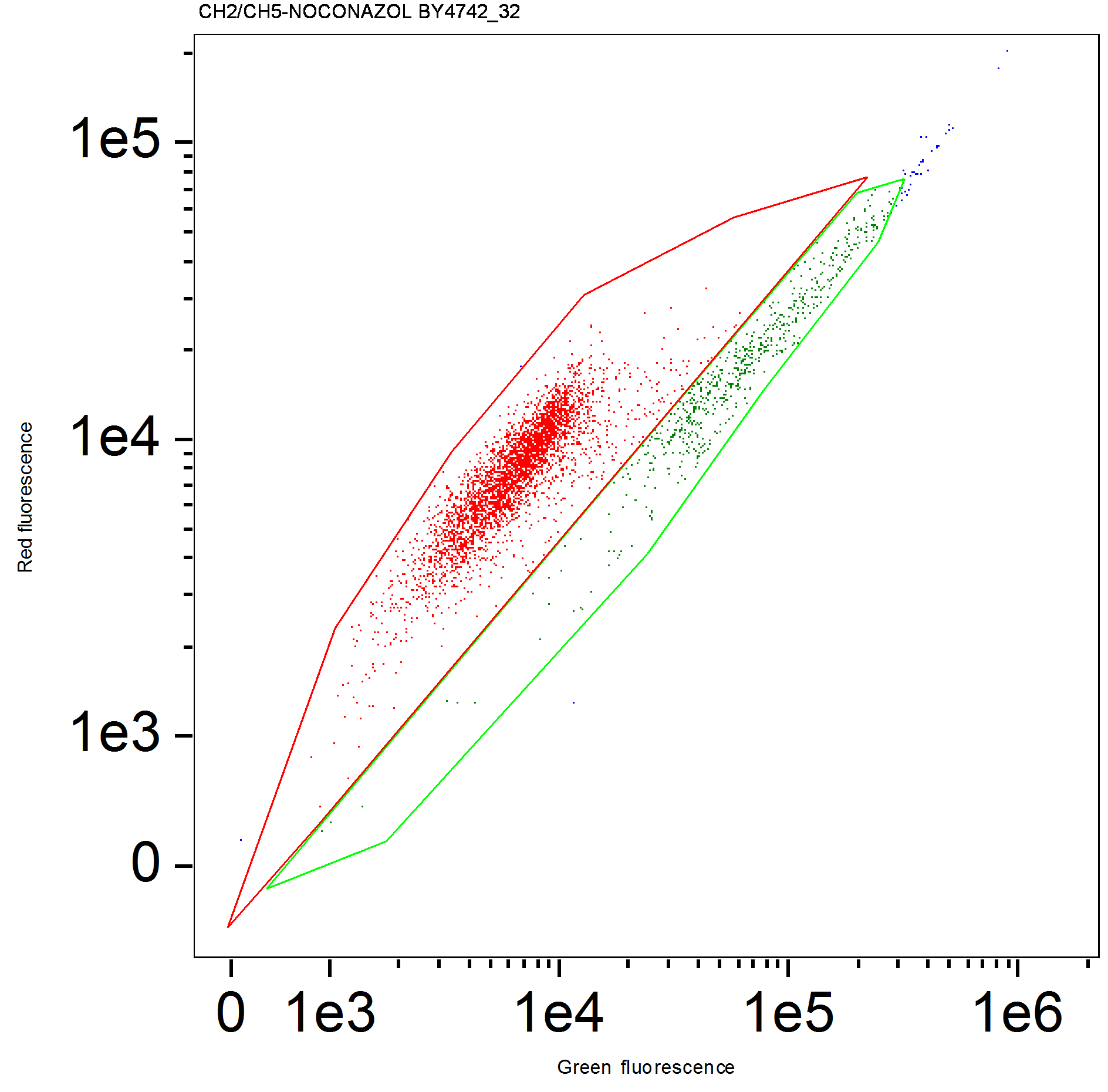

Supplement: Supplementary file 2 [file Data_Sheet_2.ZIP › Original composite images/Figure6/Viability Nocodazol.png]

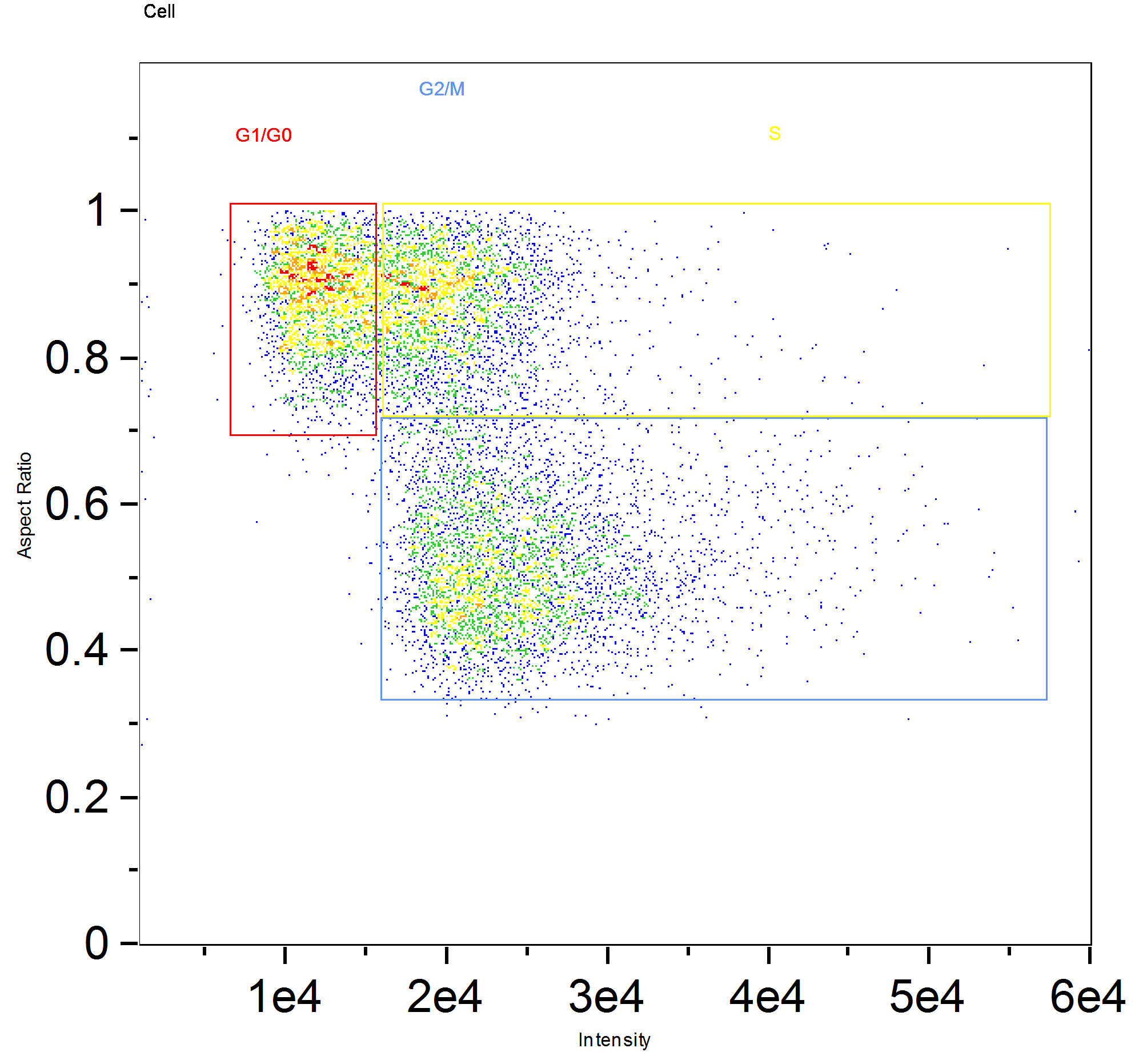

Supplement: Supplementary file 2 [file Data_Sheet_2.ZIP › Original composite images/FigureS8/Control HOD.png]

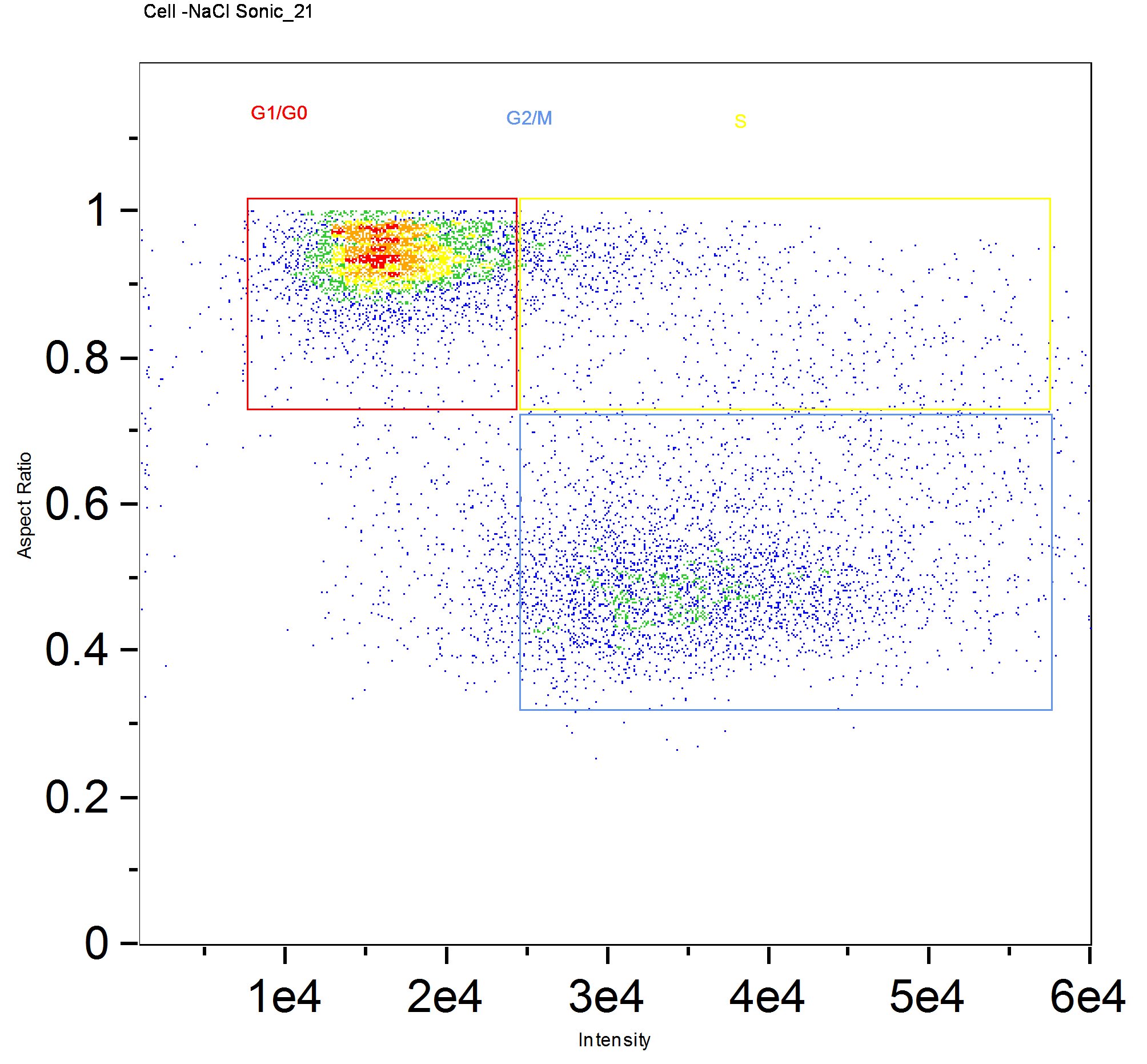

Supplement: Supplementary file 2 [file Data_Sheet_2.ZIP › Original composite images/FigureS8/NaCl HOG.png]
